# Supplementary material for: HIV incidence and associated risk factors in adolescent girls and young women in South Africa: A population-based cohort study
Source: PLoS One. 2022 Dec 21;17(12):e0279289. doi: 10.1371/journal.pone.0279289 (PMC9770356; doi:10.1371/journal.pone.0279289)
Supplement: S1 File — (PDF) [file pone.0279289.s001.pdf]

age group	completed secondary school	urban	family support	income category	uses contraception	#lifetime sex partners	pregnancy history	# partners in follow-up	condom use	circumcision	HIV+ partner	max of partner ages	transactional sex	weight	HIV +	time
20-24	No	Yes	Yes	R501 - R2,500 pm	Yes	.	Yes	one	Yes	No	No	26	No	17	No	1.9
20-24	Yes	Yes	No	R501 - R2,500 pm	No	.	No	none	.	.	.	.	.	11	No	1.8
15-19	No	Yes	Yes	R0 - R500 pm	No	.	Yes	one	No	No	No	23	No	9	No	1.9
15-19	No	Yes	Yes	R501 - R2,500 pm	No	.	No	none	.	.	.	.	.	10	No	1.6
15-19	No	No	Yes	R2,501 - R6,000 pm	No	.	No	none	.	.	.	.	.	102	Yes	0.7
15-19	Yes	Yes	Yes	R501 - R2,500 pm	Yes	.	Yes	2 or more	Yes	Yes	No	23	No	12	No	1.7
20-24	Yes	Yes	No	R501 - R2,500 pm	Yes	.	Yes	one	No	Yes	No	28	No	9	No	1.9
20-24	Yes	Yes	Yes	R0 - R500 pm	Yes	.	Yes	one	Yes	No	No	24	No	7	No	2.2
20-24	No	Yes	No	R0 - R500 pm	Yes	.	Yes	one	No	No	No	27	No	11	No	2.1
20-24	No	Yes	No	.	Yes	.	Yes	2 or more	No	No	No	25	No	9	No	2.1
20-24	Yes	No	Yes	R2,501 - R6,000 pm	No	.	No	none	.	.	.	.	.	18	No	1.5
15-19	Yes	Yes	No	R0 - R500 pm	No	.	Yes	one	No	No	No	24	Yes	9	No	1.8
15-19	No	Yes	No	R0 - R500 pm	No	.	Yes	one	Yes	No	No	24	No	8	Yes	1
15-19	No	Yes	No	R0 - R500 pm	No	.	No	one	No	No	No	23	No	12	No	1.7
15-19	No	Yes	Yes	R0 - R500 pm	Yes	.	Yes	one	No	No	No	28	No	10	Yes	1
20-24	Yes	Yes	No	R0 - R500 pm	Yes	.	No	one	No	No	No	29	No	19	No	1.9
20-24	Yes	Yes	Yes	R501 - R2,500 pm	No	.	Yes	none	.	.	.	.	.	12	Yes	1.2
20-24	Yes	Yes	No	R501 - R2,500 pm	Yes	.	No	one	No	No	No	26	No	30	No	2
15-19	Yes	No	Yes	R501 - R2,500 pm	No	.	No	none	.	.	.	.	.	21	No	1.2
15-19	Yes	Yes	No	R0 - R500 pm	No	.	No	one	No	No	No	26	No	21	No	1.6
20-24	No	Yes	No	R0 - R500 pm	No	.	No	one	No	No	No	30	No	18	No	2.3
20-24	Yes	Yes	No	R0 - R500 pm	No	.	Yes	one	No	No	No	29	No	16	No	1.8
20-24	Yes	Yes	Yes	greater than R6,000 pm	Yes	.	No	one	No	.	No	30	No	10	No	2.3
20-24	Yes	No	Yes	.	Yes	.	Yes	one	No	No	No	26	No	27	No	2
15-19	Yes	No	Yes	R2,501 - R6,000 pm	No	.	No	none	.	.	.	.	.	58	No	1.3
15-19	No	Yes	Yes	R501 - R2,500 pm	Yes	.	Yes	one	No	Yes	No	24	No	24	No	1.3
20-24	Yes	Yes	Yes	R2,501 - R6,000 pm	No	.	No	none	.	.	.	.	.	8	No	2
15-19	Yes	Yes	No	R501 - R2,500 pm	No	.	Yes	one	No	Yes	No	24	No	9	No	1.9
20-24	Yes	Yes	No	R0 - R500 pm	Yes	.	No	none	.	No	No	24	.	14	Yes	1.1
20-24	Yes	Yes	Yes	R0 - R500 pm	Yes	.	Yes	one	No	No	No	29	No	10	No	1.9
20-24	No	No	No	R0 - R500 pm	Yes	.	Yes	one	No	No	No	29	No	9	No	1.8
20-24	No	No	Yes	R501 - R2,500 pm	No	.	Yes	one	No	Yes	No	27	No	25	Yes	0.7
15-19	Yes	Yes	Yes	.	Yes	.	No	none	.	.	.	.	.	12	No	1.6
20-24	Yes	Yes	No	R501 - R2,500 pm	Yes	.	No	one	No	No	No	30	No	8	No	2
20-24	Yes	Yes	No	R0 - R500 pm	No	.	No	one	No	No	No	28	No	11	No	2
20-24	No	Yes	No	R0 - R500 pm	Yes	.	.	2 or more	No	.	No	32	No	14	No	2.2
20-24	No	Yes	Yes	R0 - R500 pm	No	.	No	none	.	.	.	.	.	17	No	1.8
20-24	Yes	Yes	No	R0 - R500 pm	No	.	No	one	No	No	No	29	No	11	No	2
20-24	Yes	Yes	No	R0 - R500 pm	No	.	Yes	2 or more	No	Yes	No	30	No	12	No	1.9
20-24	No	Yes	Yes	R0 - R500 pm	Yes	.	Yes	one	No	No	No	30	No	12	No	1.9
20-24	Yes	Yes	No	R0 - R500 pm	No	.	No	one	No	No	No	28	No	11	No	1.6
15-19	No	Yes	No	R0 - R500 pm	No	.	Yes	one	No	No	No	26	No	9	No	1.9

15-19	No	Yes	Yes	R501 - R2,500 pm	Yes	.	Yes	one	No	Yes	No	25	No	25	Yes	1
15-19	No	Yes	Yes	.	Yes	.	No	one	No	Yes	No	29	No	10	No	1.9
20-24	Yes	No	Yes	.	Yes	.	Yes	one	No	No	No	25	No	87	Yes	0.7
20-24	No	No	Yes	R0 - R500 pm	Yes	.	Yes	one	No	No	No	24	No	21	No	1.4
20-24	Yes	Yes	No	R0 - R500 pm	Yes	.	No	one	Yes	No	No	26	No	30	No	2
15-19	No	No	No	R501 - R2,500 pm	No	.	Yes	one	Yes	.	No	26	No	70	Yes	0.8
20-24	No	Yes	No	R0 - R500 pm	No	.	No	one	No	Yes	No	28	No	19	Yes	0.9
20-24	No	No	Yes	R2,501 - R6,000 pm	Yes	.	No	none	.	.	.	.	.	91	No	1.5
15-19	No	Yes	Yes	R501 - R2,500 pm	No	.	Yes	one	Yes	Yes	No	24	No	7	No	2.2
20-24	No	Yes	No	R0 - R500 pm	No	.	No	one	No	No	No	30	No	14	No	2.2
20-24	No	Yes	Yes	.	Yes	.	Yes	one	No	No	No	26	No	17	No	2.3
20-24	Yes	Yes	Yes	R501 - R2,500 pm	No	.	No	none	.	.	.	.	.	6	No	1.4
20-24	Yes	Yes	Yes	R501 - R2,500 pm	No	.	Yes	one	Yes	No	No	27	No	9	No	2.1
20-24	Yes	Yes	No	R501 - R2,500 pm	Yes	.	Yes	one	No	Yes	No	26	No	36	No	1.9
20-24	No	No	Yes	R501 - R2,500 pm	Yes	.	No	none	.	.	.	.	.	35	No	1.1
15-19	No	Yes	Yes	R2,501 - R6,000 pm	No	.	No	none	.	.	.	.	.	48	No	1.6
20-24	No	No	Yes	R2,501 - R6,000 pm	Yes	.	No	none	.	.	.	.	.	20	No	1.8
15-19	No	No	Yes	R501 - R2,500 pm	Yes	.	Yes	none	.	.	.	.	.	11	No	1.3
15-19	No	No	Yes	R2,501 - R6,000 pm	No	.	No	none	.	.	.	.	.	21	No	2.1
15-19	Yes	Yes	Yes	greater than R6,000 pm	Yes	.	No	none	.	.	.	.	.	30	No	1.4
15-19	No	Yes	Yes	greater than R6,000 pm	No	.	No	none	.	.	.	.	.	31	No	1.6
20-24	No	No	Yes	greater than R6,000 pm	Yes	.	No	none	.	.	.	.	.	59	No	1.7
15-19	No	Yes	Yes	R0 - R500 pm	Yes	.	Yes	none	.	.	.	.	.	27	No	1.8
20-24	Yes	No	Yes	R2,501 - R6,000 pm	Yes	.	No	none	.	.	.	.	.	23	No	1.1
15-19	No	No	Yes	R2,501 - R6,000 pm	No	.	No	none	.	.	.	.	.	42	No	1.2
20-24	Yes	No	Yes	greater than R6,000 pm	Yes	.	No	none	.	.	.	.	.	48	No	1.6
15-19	No	No	Yes	R501 - R2,500 pm	Yes	.	No	none	.	.	.	.	.	31	No	1.3
15-19	No	No	Yes	R501 - R2,500 pm	Yes	.	No	none	.	.	.	.	.	23	No	1.2
15-19	No	Yes	No	R2,501 - R6,000 pm	Yes	.	No	none	.	.	.	.	.	30	No	1.3
15-19	No	No	Yes	R2,501 - R6,000 pm	Yes	.	Yes	none	.	.	.	.	.	25	No	1.5
15-19	No	No	Yes	R2,501 - R6,000 pm	No	.	No	none	.	.	.	.	.	52	No	1.3
15-19	No	No	Yes	R501 - R2,500 pm	No	.	No	none	.	.	.	.	.	27	No	1.3
15-19	No	No	Yes	R501 - R2,500 pm	No	.	No	none	.	.	.	.	.	13	No	1.2
15-19	Yes	No	Yes	R2,501 - R6,000 pm	Yes	.	No	none	.	.	.	.	.	30	No	1.1
20-24	Yes	No	Yes	R501 - R2,500 pm	Yes	.	No	none	.	.	.	.	.	10	No	1.6
15-19	No	Yes	Yes	R2,501 - R6,000 pm	No	none	No	2 or more	Yes	Yes	No	20	No	10	No	1.7
20-24	Yes	Yes	Yes	R501 - R2,500 pm	No	none	No	one	No	Yes	No	27	No	21	No	1.8
15-19	Yes	Yes	Yes	R2,501 - R6,000 pm	No	none	No	one	No	Yes	No	22	No	10	No	1.7
15-19	No	Yes	Yes	R0 - R500 pm	No	none	No	one	No	No	No	28	No	9	No	2
15-19	Yes	Yes	Yes	R501 - R2,500 pm	No	none	No	one	Yes	No	No	25	No	10	No	2.1
20-24	No	Yes	Yes	R2,501 - R6,000 pm	No	none	No	none	.	.	.	.	.	20	No	1.1
15-19	No	No	Yes	.	No	none	No	one	No	.	No	18	No	24	No	1.4

20-24	Yes	No	No	R501 - R2,500 pm	No	none	No	one	No	No	No	27	No	6	No	1.2
15-19	No	No	Yes	R501 - R2,500 pm	No	none	No	one	No	Yes	No	18	No	21	No	1.2
15-19	No	Yes	Yes	R2,501 - R6,000 pm	No	none	No	none	.	.	.	.	.	19	No	1.7
20-24	Yes	No	Yes	.	No	none	No	one	Yes	No	No	27	No	23	No	1.7
15-19	Yes	Yes	No	R0 - R500 pm	No	none	No	one	No	Yes	No	22	No	11	No	1.6
15-19	Yes	No	Yes	R501 - R2,500 pm	No	none	No	none	.	.	.	.	.	7	No	1.3
15-19	No	No	Yes	greater than R6,000 pm	No	none	No	none	.	.	.	.	.	71	No	1.5
15-19	No	Yes	Yes	greater than R6,000 pm	No	none	No	one	No	Yes	No	18	No	18	No	1.2
20-24	No	Yes	No	R0 - R500 pm	No	none	No	one	No	Yes	No	26	No	13	No	2.2
15-19	Yes	Yes	Yes	R501 - R2,500 pm	No	none	No	one	No	Yes	No	21	No	26	No	1.9
15-19	Yes	No	Yes	R501 - R2,500 pm	No	none	No	none	.	.	.	.	.	17	No	1.8
15-19	No	Yes	Yes	R2,501 - R6,000 pm	No	none	No	none	.	.	.	.	.	34	No	1.4
15-19	No	Yes	Yes	R501 - R2,500 pm	No	none	No	none	.	.	.	.	.	19	No	1.6
15-19	Yes	Yes	No	R0 - R500 pm	No	none	No	one	Yes	Yes	No	26	No	16	No	1.7
15-19	No	Yes	Yes	R501 - R2,500 pm	No	none	No	none	.	.	.	.	.	30	No	1.3
15-19	No	Yes	Yes	.	No	none	No	one	No	Yes	No	23	No	50	No	2
15-19	No	Yes	Yes	R501 - R2,500 pm	No	none	No	none	.	.	.	.	.	24	No	1.8
15-19	No	Yes	Yes	R501 - R2,500 pm	No	none	No	none	.	.	.	.	.	30	No	1.2
15-19	No	Yes	No	R0 - R500 pm	No	none	No	one	No	Yes	No	24	No	39	No	2
15-19	No	No	Yes	R501 - R2,500 pm	No	none	Yes	none	.	.	.	.	.	27	No	1.7
15-19	No	Yes	Yes	R501 - R2,500 pm	No	none	No	none	.	.	.	.	.	24	No	1.3
15-19	No	No	Yes	greater than R6,000 pm	No	none	No	one	No	Yes	No	23	No	56	No	1.5
20-24	No	No	No	greater than R6,000 pm	No	none	No	none	.	.	.	.	.	40	No	1.5
15-19	Yes	No	Yes	R2,501 - R6,000 pm	No	none	No	none	.	.	.	.	.	106	No	1.3
15-19	Yes	Yes	Yes	.	No	none	No	one	No	Yes	No	26	No	22	No	1.7
15-19	No	No	Yes	R501 - R2,500 pm	No	none	No	none	.	.	.	.	.	44	No	1.2
15-19	No	Yes	Yes	R2,501 - R6,000 pm	No	none	No	one	Yes	.	Yes	21	No	30	Yes	0.9
15-19	No	Yes	Yes	R501 - R2,500 pm	No	none	No	none	.	.	.	.	.	22	No	2
20-24	No	No	Yes	R0 - R500 pm	No	none	No	none	.	.	.	.	.	76	No	1.2
15-19	No	Yes	No	R0 - R500 pm	No	none	No	one	Yes	Yes	No	20	No	13	No	2.1
15-19	No	Yes	No	R501 - R2,500 pm	No	none	No	one	No	No	No	22	No	26	No	1.1
15-19	No	No	Yes	R0 - R500 pm	No	none	No	one	No	Yes	No	27	No	47	No	1.3
15-19	No	No	Yes	R501 - R2,500 pm	No	none	No	none	.	.	.	.	.	47	No	1.4
15-19	No	Yes	No	R0 - R500 pm	No	none	No	one	Yes	No	No	15	No	9	No	1.6
15-19	No	Yes	Yes	R501 - R2,500 pm	No	none	No	none	.	.	.	.	.	22	No	1.7
20-24	Yes	Yes	Yes	greater than R6,000 pm	No	none	No	none	.	.	.	.	.	54	No	1.2
15-19	No	No	Yes	R501 - R2,500 pm	No	none	No	none	.	.	.	.	.	60	No	1.2
15-19	Yes	No	Yes	R2,501 - R6,000 pm	No	none	No	none	.	.	.	.	.	102	No	1.4
15-19	No	Yes	Yes	R501 - R2,500 pm	No	none	No	none	.	.	.	.	.	23	No	1.6
15-19	No	No	Yes	R2,501 - R6,000 pm	Yes	none	No	one	No	Yes	No	19	No	28	No	1.4
15-19	No	Yes	Yes	R501 - R2,500 pm	No	none	No	none	.	.	.	.	.	21	No	1.3

15-19	No	Yes	Yes	R501 - R2,500 pm	Yes	none	Yes	none	.	.	.	.	.	10	Yes	0.8
15-19	No	Yes	Yes	R501 - R2,500 pm	No	none	No	none	.	.	.	.	.	27	No	1.3
15-19	Yes	No	Yes	.	No	none	No	one	No	Yes	No	25	No	102	No	1.8
20-24	Yes	Yes	Yes	R2,501 - R6,000 pm	No	none	No	one	No	No	No	29	No	20	No	1.3
15-19	No	No	No	R0 - R500 pm	No	none	No	none	.	.	.	.	.	93	No	1.4
15-19	No	Yes	Yes	R2,501 - R6,000 pm	No	none	No	none	.	.	.	.	.	30	No	1.3
15-19	Yes	Yes	Yes	R0 - R500 pm	No	none	No	none	.	.	.	.	.	10	No	1.9
20-24	Yes	No	Yes	R0 - R500 pm	No	none	No	one	Yes	.	No	30	No	30	No	2.3
15-19	No	No	Yes	R501 - R2,500 pm	No	none	No	none	.	.	.	.	.	33	No	1.4
15-19	No	No	Yes	R2,501 - R6,000 pm	No	none	No	none	.	.	.	.	.	30	No	1.3
15-19	No	Yes	No	R0 - R500 pm	No	none	No	none	.	.	.	.	.	12	No	2.4
15-19	Yes	Yes	Yes	greater than R6,000 pm	No	none	No	none	.	.	.	.	.	12	No	1.4
20-24	Yes	Yes	No	R0 - R500 pm	No	none	No	one	No	Yes	No	23	No	14	Yes	0.9
20-24	Yes	Yes	Yes	R501 - R2,500 pm	No	none	No	2 or more	No	Yes	No	28	No	12	Yes	0.9
15-19	No	No	Yes	R2,501 - R6,000 pm	No	none	No	one	Yes	No	No	17	No	106	No	1.3
15-19	No	Yes	Yes	R501 - R2,500 pm	No	none	No	2 or more	Yes	No	No	26	No	11	No	1.6
20-24	Yes	No	Yes	.	No	none	No	one	No	No	No	29	No	36	No	1.8
15-19	Yes	No	Yes	.	No	none	No	none	.	.	.	.	.	60	No	1.4
15-19	No	No	Yes	R501 - R2,500 pm	No	none	.	one	Yes	Yes	No	18	No	18	No	2.1
15-19	No	Yes	Yes	R2,501 - R6,000 pm	No	none	No	one	Yes	Yes	No	19	No	19	No	1.2
20-24	Yes	No	Yes	R2,501 - R6,000 pm	No	none	No	none	.	.	.	.	.	87	No	1.5
15-19	No	Yes	No	R501 - R2,500 pm	No	none	No	none	.	.	.	.	.	31	No	1.2
15-19	No	Yes	Yes	R0 - R500 pm	No	none	No	one	Yes	.	No	22	No	49	No	1.5
15-19	No	Yes	Yes	.	No	none	No	none	.	.	.	.	.	18	No	2.1
15-19	No	No	Yes	R0 - R500 pm	No	none	No	one	Yes	.	No	21	No	65	No	1.3
20-24	Yes	Yes	No	.	Yes	none	No	one	No	Yes	Yes	28	No	10	No	1.6
15-19	No	Yes	No	R0 - R500 pm	No	none	No	one	Yes	No	No	17	No	10	No	2.5
15-19	No	No	Yes	R501 - R2,500 pm	No	none	No	none	.	.	.	.	.	72	No	1.4
15-19	No	Yes	No	greater than R6,000 pm	No	none	No	2 or more	.	Yes	No	16	No	12	No	1.9
15-19	No	No	Yes	R2,501 - R6,000 pm	No	none	No	none	.	.	.	.	.	79	No	1.5
15-19	No	Yes	Yes	R501 - R2,500 pm	No	none	No	one	Yes	Yes	No	21	No	15	No	2.1
15-19	No	Yes	No	R501 - R2,500 pm	No	none	.	one	No	Yes	No	28	No	10	No	2.4
15-19	No	Yes	Yes	R0 - R500 pm	No	none	No	none	.	.	.	.	.	12	No	2.2
15-19	No	Yes	Yes	greater than R6,000 pm	No	none	No	none	.	.	.	.	.	8	No	1.3
15-19	No	No	Yes	.	No	none	No	one	Yes	Yes	No	18	No	74	No	1.9
15-19	No	Yes	Yes	.	No	none	No	none	.	.	.	.	.	14	No	2.1
15-19	Yes	Yes	Yes	R2,501 - R6,000 pm	No	none	No	none	.	.	.	.	.	24	No	1.9
15-19	No	Yes	No	R501 - R2,500 pm	No	none	No	one	No	Yes	No	27	No	15	No	2.1
15-19	No	Yes	Yes	R501 - R2,500 pm	No	none	No	none	.	.	.	.	.	24	No	1.2
20-24	No	Yes	Yes	greater than R6,000 pm	No	none	No	2 or more	No	.	No	28	No	10	No	1.7
20-24	Yes	No	Yes	greater than R6,000 pm	No	none	No	none	.	.	.	.	.	71	No	1.2

15-19	No	Yes	Yes	R501 - R2,500 pm	No	none	No	none	.	.	.	.	.	27	No	1
15-19	No	Yes	Yes	R501 - R2,500 pm	No	none	Yes	one	Yes	No	No	23	No	10	No	2.1
20-24	Yes	Yes	Yes	R501 - R2,500 pm	No	none	No	none	.	.	.	.	.	11	No	1.9
15-19	Yes	Yes	Yes	R501 - R2,500 pm	No	none	No	none	.	.	.	.	.	20	No	1.4
20-24	Yes	No	Yes	R501 - R2,500 pm	No	none	No	none	.	.	.	.	.	43	No	1.4
15-19	Yes	No	Yes	R501 - R2,500 pm	No	none	Yes	one	No	No	No	23	Yes	106	No	1.5
15-19	No	Yes	Yes	R501 - R2,500 pm	No	none	No	none	.	.	.	.	.	9	No	1.9
15-19	No	Yes	Yes	R2,501 - R6,000 pm	No	none	No	none	.	.	.	.	.	27	No	2.3
15-19	No	Yes	Yes	greater than R6,000 pm	No	none	No	one	No	Yes	No	23	No	37	No	1.2
15-19	Yes	Yes	Yes	R2,501 - R6,000 pm	No	none	No	one	No	Yes	No	20	No	33	No	1.9
15-19	No	No	Yes	R501 - R2,500 pm	No	none	No	one	No	Yes	No	18	No	39	No	2.1
20-24	Yes	Yes	Yes	greater than R6,000 pm	No	none	No	none	.	.	.	.	.	24	No	1.6
15-19	No	No	Yes	R501 - R2,500 pm	No	none	No	one	Yes	Yes	No	24	No	26	No	1.3
20-24	Yes	Yes	Yes	greater than R6,000 pm	No	none	No	none	.	.	.	.	.	34	No	1.2
15-19	No	Yes	No	.	No	none	No	none	.	.	.	.	.	14	No	1.9
20-24	No	Yes	Yes	R501 - R2,500 pm	No	none	No	none	.	.	.	.	.	21	No	1.3
15-19	No	Yes	Yes	.	No	none	No	one	No	Yes	No	23	No	45	No	2.1
15-19	No	Yes	No	R501 - R2,500 pm	No	none	No	one	No	.	No	18	No	33	No	1.8
15-19	No	Yes	Yes	R2,501 - R6,000 pm	No	none	No	none	.	.	.	.	.	27	No	1.3
15-19	Yes	Yes	No	R0 - R500 pm	No	none	No	none	.	.	.	.	.	15	No	2.1
15-19	No	Yes	Yes	R501 - R2,500 pm	No	none	No	one	Yes	Yes	No	21	No	12	No	2.1
15-19	Yes	Yes	No	R0 - R500 pm	No	none	No	none	.	.	.	.	.	33	No	2
15-19	No	No	Yes	R501 - R2,500 pm	No	none	No	none	.	.	.	.	.	11	No	1.1
15-19	Yes	No	Yes	R2,501 - R6,000 pm	No	none	No	none	.	.	.	.	.	98	No	1.4
15-19	No	Yes	Yes	R501 - R2,500 pm	No	none	No	none	.	.	.	.	.	21	No	1.9
15-19	No	Yes	Yes	R501 - R2,500 pm	No	none	No	one	No	Yes	No	22	No	11	No	1.7
15-19	No	Yes	Yes	R0 - R500 pm	No	none	No	one	No	Yes	No	23	No	79	No	2.4
15-19	No	Yes	Yes	.	No	none	No	none	.	.	.	.	.	12	No	1.6
20-24	Yes	No	Yes	R501 - R2,500 pm	No	none	No	one	No	No	No	25	Yes	54	No	1.4
15-19	No	Yes	Yes	R2,501 - R6,000 pm	No	none	No	none	.	.	.	.	.	46	No	1.3
15-19	Yes	Yes	Yes	R501 - R2,500 pm	No	none	No	none	.	.	.	.	.	36	No	1.2
15-19	No	No	Yes	R2,501 - R6,000 pm	No	none	No	none	.	.	.	.	.	21	No	1.2
15-19	No	Yes	No	R2,501 - R6,000 pm	Yes	none	No	none	.	.	.	.	.	20	No	1.2
20-24	No	Yes	Yes	R2,501 - R6,000 pm	No	none	Yes	one	Yes	Yes	No	22	No	24	No	1.5
15-19	No	No	Yes	R501 - R2,500 pm	No	none	No	none	.	.	.	.	.	102	No	1.4
15-19	Yes	No	Yes	R501 - R2,500 pm	No	none	No	none	.	.	.	.	.	33	No	2
15-19	No	No	No	R501 - R2,500 pm	No	none	No	none	.	.	.	.	.	106	No	1.3
15-19	No	No	Yes	R2,501 - R6,000 pm	No	none	No	none	.	.	.	.	.	106	No	1.5
15-19	No	Yes	Yes	greater than R6,000 pm	No	none	No	one	No	Yes	No	21	No	34	No	1.8
15-19	No	No	Yes	R2,501 - R6,000 pm	No	none	No	none	.	.	.	.	.	15	No	1.7
20-24	Yes	No	Yes	R0 - R500 pm	No	none	No	one	No	Yes	No	27	No	28	No	1.8
20-24	No	Yes	Yes	.	Yes	none	Yes	none	.	.	.	.	.	9	No	1.9

15-19	Yes	Yes	Yes	R501 - R2,500 pm	No	none	No	2 or more	Yes	No	No	25	No	9	No	1.9
15-19	No	No	Yes	R501 - R2,500 pm	No	none	No	none	.	.	.	.	.	24	No	1.4
20-24	Yes	Yes	Yes	R501 - R2,500 pm	No	none	No	one	Yes	No	No	23	No	16	No	1.6
15-19	No	No	Yes	R0 - R500 pm	No	none	No	none	.	.	.	.	.	31	No	1.4
15-19	No	No	Yes	R501 - R2,500 pm	No	none	No	none	.	.	.	.	.	28	No	1.7
20-24	Yes	Yes	Yes	R2,501 - R6,000 pm	No	none	No	none	.	.	.	.	.	8	No	1.3
15-19	No	No	Yes	R501 - R2,500 pm	No	none	No	one	Yes	Yes	No	23	No	33	No	1.5
15-19	Yes	Yes	Yes	greater than R6,000 pm	No	none	No	none	.	.	.	.	.	19	Yes	0.6
15-19	No	Yes	Yes	R501 - R2,500 pm	No	none	No	none	.	.	.	.	.	16	No	1.2
20-24	Yes	Yes	No	R0 - R500 pm	No	none	No	one	Yes	Yes	No	26	No	7	No	1.9
15-19	No	Yes	No	R2,501 - R6,000 pm	No	none	.	none	.	.	.	.	.	9	No	1.8
15-19	Yes	No	Yes	R2,501 - R6,000 pm	No	none	No	one	Yes	Yes	No	21	No	55	No	1.6
15-19	Yes	Yes	Yes	R0 - R500 pm	No	none	No	one	Yes	Yes	No	23	No	11	No	1.2
15-19	Yes	No	Yes	R501 - R2,500 pm	No	none	No	one	Yes	No	No	26	No	60	No	1.7
15-19	Yes	No	Yes	R501 - R2,500 pm	No	none	No	one	No	No	No	29	No	27	Yes	1.1
15-19	No	Yes	Yes	R501 - R2,500 pm	No	none	No	none	.	.	.	.	.	9	No	1.3
20-24	Yes	Yes	Yes	.	No	none	No	none	.	.	.	.	.	20	Yes	0.8
15-19	Yes	Yes	Yes	R2,501 - R6,000 pm	No	none	No	none	.	.	.	.	.	14	No	1.8
15-19	Yes	No	Yes	greater than R6,000 pm	No	none	No	one	Yes	Yes	No	20	No	106	No	1.4
15-19	No	Yes	Yes	.	No	none	No	none	.	.	.	.	.	14	No	1.8
15-19	No	No	Yes	R501 - R2,500 pm	No	none	No	none	.	.	.	.	.	44	No	1.4
15-19	Yes	Yes	Yes	R501 - R2,500 pm	No	none	No	one	Yes	Yes	No	23	No	32	No	1.9
15-19	No	No	Yes	R501 - R2,500 pm	No	none	No	one	No	.	No	23	No	16	No	1.6
20-24	No	Yes	Yes	R0 - R500 pm	No	none	No	one	Yes	Yes	No	31	No	12	No	1.8
15-19	No	Yes	Yes	R2,501 - R6,000 pm	No	none	No	none	.	.	.	.	.	7	No	1.9
15-19	No	No	Yes	greater than R6,000 pm	No	none	No	none	.	.	.	.	.	50	No	1.7
15-19	No	Yes	Yes	R2,501 - R6,000 pm	No	none	No	none	.	.	.	.	.	30	No	1.9
15-19	No	Yes	Yes	R501 - R2,500 pm	No	none	No	one	Yes	Yes	No	20	No	20	No	1.7
15-19	No	No	Yes	R2,501 - R6,000 pm	No	none	No	none	.	.	.	.	.	59	No	1.5
20-24	Yes	Yes	Yes	R501 - R2,500 pm	No	none	No	one	No	Yes	No	24	No	30	No	1.3
20-24	Yes	No	Yes	R501 - R2,500 pm	No	none	Yes	none	.	.	.	.	.	16	No	1.8
20-24	Yes	No	No	R0 - R500 pm	No	none	No	one	No	.	No	23	Yes	31	No	1.8
15-19	No	Yes	No	R501 - R2,500 pm	No	none	No	none	.	.	.	.	.	44	No	1.8
20-24	No	Yes	No	R0 - R500 pm	No	none	No	one	No	No	No	38	No	8	No	2
15-19	Yes	Yes	Yes	R501 - R2,500 pm	No	none	No	one	No	Yes	No	24	No	16	No	1.7
15-19	No	Yes	No	R501 - R2,500 pm	No	none	No	one	Yes	Yes	No	20	Yes	19	No	1.8
15-19	No	No	Yes	R501 - R2,500 pm	No	none	No	none	.	.	.	.	.	106	No	1.7
15-19	No	No	Yes	.	No	none	No	one	No	Yes	No	24	No	64	No	1.4
15-19	No	Yes	Yes	R501 - R2,500 pm	No	none	No	none	.	.	.	.	.	7	No	1.2
15-19	No	No	Yes	R2,501 - R6,000 pm	No	none	No	none	.	.	.	.	.	35	No	1.7
15-19	No	Yes	Yes	R501 - R2,500 pm	No	none	No	none	.	.	.	.	.	39	No	1.2
20-24	Yes	No	Yes	R2,501 - R6,000 pm	No	none	No	one	No	No	No	18	No	23	No	1.2
15-19	Yes	Yes	Yes	R501 - R2,500 pm	No	none	No	none	.	.	.	.	.	16	No	1.9

15-19	No	Yes	Yes	R501 - R2,500 pm	No	none	No	none	.	.	.	.	.	21	No	1.2
15-19	No	No	Yes	R501 - R2,500 pm	No	none	No	none	.	.	.	.	.	106	No	1.9
15-19	No	Yes	Yes	R2,501 - R6,000 pm	No	none	No	none	.	.	.	.	.	12	No	1.4
15-19	Yes	Yes	No	R0 - R500 pm	No	none	No	one	Yes	Yes	No	28	No	11	No	2
15-19	No	No	Yes	R2,501 - R6,000 pm	No	none	No	none	.	.	.	.	.	36	No	1.8
15-19	Yes	Yes	Yes	R501 - R2,500 pm	No	none	No	2 or more	Yes	Yes	No	21	No	13	No	2.2
15-19	No	Yes	Yes	R501 - R2,500 pm	No	none	No	one	Yes	Yes	No	23	No	16	No	1.2
15-19	No	Yes	No	.	No	none	No	one	No	Yes	No	18	No	18	No	1.6
15-19	Yes	No	No	.	No	none	No	2 or more	No	Yes	No	20	No	30	No	1.7
15-19	No	Yes	Yes	R2,501 - R6,000 pm	No	none	No	one	No	.	No	21	No	8	No	1.6
15-19	No	Yes	Yes	R501 - R2,500 pm	No	none	No	none	.	.	.	.	.	22	No	2
15-19	No	No	Yes	R501 - R2,500 pm	No	none	No	none	.	.	.	.	.	39	No	1.4
20-24	Yes	Yes	Yes	.	No	none	No	2 or more	No	Yes	No	26	No	10	No	2.1
15-19	No	Yes	Yes	.	No	none	No	none	.	.	.	.	.	10	No	1.9
20-24	Yes	No	Yes	.	No	none	No	one	No	Yes	No	24	No	29	No	2.1
20-24	Yes	Yes	No	R501 - R2,500 pm	No	none	No	none	Yes	.	.	.	No	29	No	1.1
20-24	No	Yes	Yes	.	No	none	No	none	.	.	.	.	.	17	No	1.6
15-19	No	No	Yes	.	No	none	No	one	No	No	No	22	No	19	No	1.3
15-19	No	No	Yes	R0 - R500 pm	No	none	No	none	.	.	.	.	.	33	No	2
15-19	No	No	Yes	R501 - R2,500 pm	No	none	No	2 or more	No	Yes	No	26	No	29	No	1.8
15-19	No	No	Yes	R501 - R2,500 pm	No	none	No	one	No	.	No	20	No	28	No	1.4
20-24	No	Yes	No	R0 - R500 pm	No	none	No	one	No	Yes	No	24	No	14	No	1.9
15-19	No	Yes	Yes	R2,501 - R6,000 pm	No	none	No	none	.	.	.	.	.	67	No	2
15-19	No	No	Yes	greater than R6,000 pm	No	none	No	none	.	.	.	.	.	22	No	2.3
15-19	Yes	No	Yes	R0 - R500 pm	No	none	No	none	.	.	.	.	.	102	No	1.5
15-19	No	No	Yes	R2,501 - R6,000 pm	No	none	No	one	No	Yes	No	20	No	64	No	1.7
15-19	No	No	Yes	R501 - R2,500 pm	No	none	No	one	No	Yes	No	25	Yes	55	No	1.6
15-19	No	No	Yes	R2,501 - R6,000 pm	No	none	No	none	.	.	.	.	.	32	No	1.8
15-19	No	No	Yes	.	Yes	none	Yes	one	No	Yes	No	27	Yes	39	No	2
15-19	No	Yes	Yes	R2,501 - R6,000 pm	No	none	No	one	Yes	Yes	No	35	No	10	No	1.6
20-24	Yes	Yes	Yes	R501 - R2,500 pm	No	none	No	none	.	.	.	.	.	23	No	1.3
15-19	Yes	No	Yes	R501 - R2,500 pm	No	none	No	one	No	Yes	No	23	No	40	No	1.5
20-24	Yes	No	Yes	R501 - R2,500 pm	No	none	No	none	.	.	.	.	.	44	No	1.6
15-19	No	Yes	Yes	R501 - R2,500 pm	No	none	No	one	Yes	Yes	No	34	No	13	No	2.1
15-19	No	Yes	Yes	R501 - R2,500 pm	No	none	No	none	.	.	.	.	.	7	No	1.2
15-19	No	Yes	Yes	R2,501 - R6,000 pm	No	none	No	none	.	.	.	.	.	28	No	1.3
15-19	No	No	Yes	R2,501 - R6,000 pm	No	none	No	none	.	.	.	.	.	106	No	1.5
15-19	No	No	Yes	R2,501 - R6,000 pm	No	none	No	none	.	.	.	.	.	56	No	1.3
20-24	Yes	No	Yes	R501 - R2,500 pm	No	none	No	one	No	No	No	36	No	30	No	1.8
15-19	No	Yes	Yes	R2,501 - R6,000 pm	No	none	No	none	.	.	.	.	.	22	No	1.3
15-19	No	No	Yes	R2,501 - R6,000 pm	No	none	No	none	.	.	.	.	.	106	No	1.5
15-19	No	Yes	Yes	R501 - R2,500 pm	No	none	No	none	.	.	.	.	.	13	No	1.6
15-19	Yes	Yes	Yes	greater than R6,000 pm	No	none	No	none	.	.	.	.	.	20	No	1.5
20-24	Yes	Yes	Yes	R501 - R2,500 pm	No	none	No	one	Yes	No	No	24	No	15	No	1.6

15-19	No	Yes	No	R0 - R500 pm	No	none	No	none	.	.	.	.	.	9	No	1.6
15-19	No	Yes	No	R501 - R2,500 pm	No	none	Yes	one	No	No	No	25	Yes	17	Yes	1.2
20-24	Yes	No	Yes	R501 - R2,500 pm	No	none	No	one	No	No	No	30	No	61	No	1.5
15-19	No	Yes	Yes	R501 - R2,500 pm	No	none	No	none	.	.	.	.	.	32	No	1.9
15-19	No	No	Yes	.	No	none	No	none	.	.	.	.	.	50	No	1.5
15-19	No	Yes	Yes	R2,501 - R6,000 pm	No	none	No	none	.	.	.	.	.	47	No	1.5
15-19	No	Yes	Yes	R0 - R500 pm	No	none	No	none	.	.	.	.	.	13	No	1.7
15-19	No	Yes	Yes	R2,501 - R6,000 pm	No	none	No	none	.	.	.	.	.	17	No	1.6
15-19	No	No	Yes	R0 - R500 pm	No	none	No	none	.	.	.	.	.	101	No	2
15-19	No	Yes	Yes	greater than R6,000 pm	No	none	No	none	.	.	.	.	.	18	No	2.3
15-19	Yes	Yes	Yes	R501 - R2,500 pm	No	none	No	none	.	.	.	.	.	9	No	1.9
15-19	No	Yes	Yes	R2,501 - R6,000 pm	No	none	No	none	.	.	.	.	.	20	No	1.2
15-19	No	No	Yes	R2,501 - R6,000 pm	No	none	No	one	Yes	Yes	No	21	No	56	No	1.4
15-19	Yes	No	No	R0 - R500 pm	No	none	No	none	.	.	.	.	.	33	No	1.8
15-19	No	Yes	Yes	R501 - R2,500 pm	No	none	No	none	.	.	.	.	.	17	No	1.2
15-19	No	Yes	Yes	.	No	none	No	none	.	.	.	.	.	17	No	1.8
15-19	Yes	No	Yes	R0 - R500 pm	No	none	No	none	.	.	.	.	.	51	No	1.3
15-19	No	Yes	Yes	R501 - R2,500 pm	No	none	No	one	Yes	No	No	20	No	25	No	1.3
15-19	Yes	Yes	Yes	R501 - R2,500 pm	No	none	No	none	.	.	.	.	.	15	No	2.1
15-19	No	Yes	Yes	R501 - R2,500 pm	No	none	No	none	.	.	.	.	.	13	No	1.7
15-19	No	Yes	Yes	.	No	none	Yes	one	No	No	No	24	No	10	No	1.9
15-19	Yes	No	Yes	R501 - R2,500 pm	No	none	No	none	.	.	.	.	.	64	No	1.3
15-19	No	Yes	Yes	R2,501 - R6,000 pm	No	none	No	none	.	.	.	.	.	45	No	1.5
15-19	No	Yes	Yes	R2,501 - R6,000 pm	No	none	No	none	.	.	.	.	.	28	No	1.3
20-24	Yes	Yes	No	R0 - R500 pm	No	none	No	one	No	No	No	25	No	12	No	1.9
20-24	Yes	No	Yes	R501 - R2,500 pm	No	none	No	one	No	Yes	No	23	No	30	No	1.4
15-19	No	Yes	Yes	greater than R6,000 pm	No	none	No	one	No	No	No	20	No	29	No	1.9
15-19	No	Yes	Yes	R501 - R2,500 pm	No	none	No	none	.	.	.	.	.	18	No	1.4
15-19	No	Yes	Yes	.	No	none	No	2 or more	No	Yes	No	32	No	15	No	2.3
15-19	No	Yes	Yes	R2,501 - R6,000 pm	No	none	No	one	Yes	No	No	23	No	18	No	1.6
15-19	No	Yes	Yes	.	No	none	No	none	.	.	.	.	.	17	No	2
15-19	Yes	No	Yes	R501 - R2,500 pm	No	none	No	none	.	.	.	.	.	106	No	1.2
15-19	No	Yes	No	R0 - R500 pm	No	none	No	one	No	.	No	35	No	16	Yes	1.1
15-19	No	Yes	No	R0 - R500 pm	No	none	No	one	No	Yes	No	22	No	16	Yes	0.9
15-19	No	Yes	Yes	.	No	none	No	one	No	Yes	No	21	No	74	No	2
15-19	No	Yes	Yes	greater than R6,000 pm	No	none	No	one	No	Yes	No	17	Yes	10	No	1.6
20-24	Yes	Yes	Yes	R0 - R500 pm	No	none	No	none	.	.	.	.	.	10	No	1.9
15-19	No	No	Yes	R501 - R2,500 pm	No	none	No	none	.	.	.	.	.	35	Yes	0.9
15-19	Yes	Yes	Yes	R501 - R2,500 pm	No	none	No	one	Yes	Yes	No	26	No	12	No	1.2
15-19	No	No	Yes	R501 - R2,500 pm	No	none	No	none	.	.	.	.	.	7	No	1.2
15-19	Yes	No	Yes	R501 - R2,500 pm	No	none	No	one	Yes	Yes	No	25	No	44	No	1.3
15-19	No	No	Yes	.	No	none	No	none	.	.	.	.	.	8	No	2
15-19	Yes	No	No	R501 - R2,500 pm	No	none	Yes	none	.	.	.	.	.	26	No	1.8

15-19	Yes	Yes	Yes	R501 - R2,500 pm	No	none	No	none	.	.	.	.	.	12	No	1.6
15-19	No	No	Yes	R2,501 - R6,000 pm	No	none	No	none	.	.	.	.	.	67	No	1.8
15-19	Yes	Yes	No	R501 - R2,500 pm	No	none	No	one	No	.	No	18	No	13	No	1.9
20-24	Yes	No	No	R2,501 - R6,000 pm	No	none	No	none	.	.	.	.	.	16	No	1.2
20-24	Yes	No	Yes	R0 - R500 pm	No	none	No	one	No	Yes	No	27	No	61	No	1.3
15-19	No	Yes	Yes	R501 - R2,500 pm	No	none	No	none	.	.	.	.	.	29	No	1.9
20-24	No	No	No	R0 - R500 pm	No	none	No	one	No	.	No	22	No	31	No	1.8
15-19	No	Yes	Yes	R501 - R2,500 pm	No	none	No	one	No	Yes	No	20	No	29	No	1.5
15-19	No	Yes	No	R0 - R500 pm	No	none	No	none	.	.	.	.	.	33	No	2
15-19	No	No	Yes	R501 - R2,500 pm	No	none	No	none	.	.	.	.	.	17	No	1.8
15-19	No	Yes	Yes	R501 - R2,500 pm	No	none	No	one	Yes	Yes	No	23	No	12	No	1.7
15-19	No	Yes	No	R0 - R500 pm	No	none	No	none	.	.	.	.	.	10	No	1.6
15-19	No	No	Yes	R501 - R2,500 pm	No	none	No	one	No	Yes	No	22	No	99	No	2.1
15-19	No	No	Yes	R501 - R2,500 pm	No	none	No	one	No	No	No	22	Yes	48	No	1.3
15-19	No	No	Yes	R2,501 - R6,000 pm	No	none	No	one	Yes	Yes	No	25	No	31	No	1.2
15-19	No	Yes	Yes	R0 - R500 pm	No	none	No	none	.	.	.	.	.	12	No	1.9
15-19	No	Yes	Yes	R501 - R2,500 pm	No	none	No	none	.	.	.	.	.	16	No	1.2
20-24	Yes	Yes	No	R2,501 - R6,000 pm	No	none	No	one	No	Yes	No	25	No	17	No	1.8
20-24	Yes	Yes	No	R2,501 - R6,000 pm	No	none	No	one	No	No	No	39	No	12	No	2.1
15-19	Yes	Yes	No	R0 - R500 pm	No	none	No	one	No	Yes	No	20	No	21	No	1.6
15-19	Yes	Yes	Yes	R501 - R2,500 pm	No	none	No	2 or more	No	Yes	No	19	No	26	No	1.3
15-19	No	No	Yes	R501 - R2,500 pm	No	none	No	none	.	.	.	.	.	26	No	1.3
15-19	Yes	Yes	No	R0 - R500 pm	No	none	No	none	.	.	.	.	.	25	No	1.3
15-19	No	No	Yes	R2,501 - R6,000 pm	No	none	No	none	.	.	.	.	.	33	No	2
15-19	No	Yes	No	R0 - R500 pm	No	none	No	none	.	.	.	.	.	13	No	2.1
15-19	No	Yes	Yes	R0 - R500 pm	No	none	No	one	Yes	Yes	No	20	No	30	No	1.6
20-24	Yes	No	Yes	.	Yes	none	No	one	No	Yes	No	26	No	23	No	1.8
15-19	No	No	Yes	R0 - R500 pm	No	none	No	none	.	.	.	.	.	27	No	1.4
15-19	No	Yes	Yes	R501 - R2,500 pm	No	none	No	one	No	Yes	Yes	21	No	13	No	1.9
15-19	No	Yes	Yes	R501 - R2,500 pm	No	none	No	none	.	.	.	.	.	31	No	1.2
20-24	Yes	No	Yes	.	No	none	No	2 or more	No	.	No	27	No	8	No	2.1
15-19	No	No	Yes	R501 - R2,500 pm	No	none	No	none	.	.	.	.	.	90	No	1.9
15-19	No	Yes	Yes	R501 - R2,500 pm	No	none	No	one	No	Yes	No	18	No	21	No	1.7
15-19	Yes	Yes	Yes	R501 - R2,500 pm	No	none	No	none	.	.	.	.	.	33	No	1.9
15-19	No	No	Yes	R501 - R2,500 pm	No	none	No	none	.	.	.	.	.	24	No	1.8
15-19	No	Yes	Yes	R501 - R2,500 pm	No	none	No	none	.	.	.	.	.	11	No	1.9
20-24	Yes	No	No	R0 - R500 pm	No	none	No	one	No	Yes	No	26	No	26	No	1.8
15-19	Yes	Yes	No	R0 - R500 pm	No	none	No	one	No	Yes	No	26	No	11	No	2.3
15-19	No	Yes	Yes	R0 - R500 pm	No	none	No	none	.	.	.	.	.	14	No	1.3
20-24	No	Yes	No	R0 - R500 pm	No	none	No	one	No	Yes	No	24	No	10	No	1.7
15-19	Yes	Yes	Yes	R501 - R2,500 pm	No	none	No	none	.	.	.	.	.	9	No	2.2
15-19	No	No	Yes	R501 - R2,500 pm	No	none	No	none	.	.	.	.	.	46	No	1.5
15-19	No	Yes	Yes	R501 - R2,500 pm	No	none	No	none	.	.	.	.	.	33	No	1.8
20-24	Yes	No	Yes	R501 - R2,500 pm	No	none	No	one	Yes	Yes	No	26	No	57	No	2.3
15-19	Yes	Yes	Yes	R501 - R2,500 pm	No	none	No	none	.	.	.	.	.	16	No	1.3
15-19	Yes	No	Yes	R501 - R2,500 pm	No	none	No	none	.	.	.	.	.	55	No	1.3

20-24	No	Yes	Yes	R2,501 - R6,000 pm	No	none	No	none	.	.	.	.	.	45	No	1.1
15-19	No	Yes	Yes	R0 - R500 pm	No	none	No	none	.	.	.	.	.	10	No	2.1
15-19	No	Yes	Yes	.	No	none	No	none	.	.	.	.	.	26	No	1.9
15-19	No	No	Yes	R2,501 - R6,000 pm	No	none	No	none	.	.	.	.	.	31	No	1.7
15-19	No	No	Yes	R0 - R500 pm	No	none	No	one	No	Yes	No	20	No	94	No	2
15-19	No	No	Yes	R501 - R2,500 pm	No	none	No	one	Yes	No	No	22	No	55	No	1.5
15-19	No	Yes	Yes	R2,501 - R6,000 pm	No	none	No	none	.	.	.	.	.	57	No	1.9
15-19	No	Yes	Yes	R501 - R2,500 pm	No	none	No	one	Yes	Yes	No	24	No	27	No	1.3
15-19	No	No	Yes	R2,501 - R6,000 pm	No	none	No	none	.	.	.	.	.	102	No	1.7
15-19	No	No	Yes	R2,501 - R6,000 pm	No	none	No	none	.	.	.	.	.	67	No	1.5
15-19	No	Yes	Yes	.	No	none	No	none	.	.	.	.	.	38	No	1.3
15-19	Yes	No	Yes	.	No	none	Yes	none	.	.	.	.	.	23	No	1.3
20-24	No	Yes	No	R0 - R500 pm	No	none	No	one	No	Yes	No	25	No	18	No	1.9
15-19	Yes	Yes	Yes	.	No	none	No	none	.	.	.	.	.	22	No	2.4
15-19	Yes	Yes	No	R0 - R500 pm	No	none	No	none	.	.	.	.	.	10	No	1.9
15-19	No	No	Yes	R501 - R2,500 pm	No	none	No	none	.	.	.	.	.	16	No	1.2
15-19	No	Yes	Yes	R501 - R2,500 pm	No	none	No	one	No	No	No	25	No	14	No	2.1
15-19	No	Yes	Yes	R2,501 - R6,000 pm	No	none	No	none	.	.	.	.	.	20	No	2.2
15-19	No	Yes	Yes	R2,501 - R6,000 pm	No	none	No	none	.	.	.	.	.	10	No	1.7
20-24	Yes	Yes	No	R0 - R500 pm	No	none	No	one	Yes	Yes	No	35	No	30	No	1.7
20-24	Yes	Yes	Yes	.	No	none	No	none	.	.	.	.	.	12	No	2.2
15-19	No	Yes	No	.	No	none	No	one	No	Yes	No	22	No	14	No	2
15-19	No	No	Yes	R2,501 - R6,000 pm	No	none	No	none	.	.	.	.	.	84	No	1.8
20-24	No	Yes	No	R501 - R2,500 pm	No	none	No	one	No	.	No	25	No	16	No	1.2
20-24	Yes	Yes	Yes	.	No	none	No	one	No	Yes	No	23	No	13	No	1.8
15-19	No	Yes	Yes	R501 - R2,500 pm	No	none	No	one	Yes	Yes	No	20	No	45	No	1.3
15-19	No	Yes	Yes	R2,501 - R6,000 pm	No	none	No	none	.	.	.	.	.	17	No	1.3
15-19	Yes	Yes	Yes	greater than R6,000 pm	No	none	No	none	.	.	.	.	.	13	No	2.3
15-19	No	Yes	Yes	R501 - R2,500 pm	No	none	No	one	Yes	No	No	19	No	44	No	1.6
15-19	Yes	Yes	Yes	R2,501 - R6,000 pm	No	none	No	one	No	Yes	No	23	No	17	No	1.2
15-19	No	Yes	No	R501 - R2,500 pm	No	none	No	none	.	.	.	.	.	13	No	2.5
15-19	No	Yes	Yes	R0 - R500 pm	No	none	No	one	No	No	No	19	No	17	No	1.2
15-19	Yes	Yes	Yes	R501 - R2,500 pm	No	none	No	one	No	Yes	No	23	No	12	No	1.8
20-24	Yes	Yes	No	R501 - R2,500 pm	No	none	No	none	.	.	.	.	.	11	No	1.2
15-19	No	No	Yes	R501 - R2,500 pm	No	none	No	one	No	Yes	No	18	No	55	No	1.6
20-24	Yes	No	Yes	R0 - R500 pm	No	none	No	one	No	Yes	No	25	No	16	No	1.7
15-19	Yes	Yes	Yes	R0 - R500 pm	No	none	No	one	No	No	No	25	No	21	No	1.8
15-19	No	No	Yes	R0 - R500 pm	No	none	No	none	.	.	.	.	.	24	No	1.3
20-24	Yes	Yes	Yes	R501 - R2,500 pm	No	none	No	none	.	.	.	.	.	9	No	1.5
15-19	No	Yes	Yes	greater than R6,000 pm	No	none	No	none	.	.	.	.	.	12	No	1.9
15-19	No	Yes	Yes	.	No	none	No	one	No	Yes	No	21	No	19	No	1.9
15-19	Yes	No	Yes	R2,501 - R6,000 pm	No	none	No	one	No	Yes	No	22	No	7	No	1.1
15-19	No	Yes	Yes	R501 - R2,500 pm	No	none	No	none	.	.	.	.	.	38	No	1.3
15-19	Yes	Yes	Yes	R501 - R2,500 pm	No	none	No	none	.	.	.	.	.	12	No	1.6

15-19	Yes	Yes	Yes	R501 - R2,500 pm	No	none	No	none	.	.	.	.	.	10	No	2
20-24	Yes	Yes	Yes	R0 - R500 pm	No	none	No	one	No	Yes	No	26	No	14	No	1.9
15-19	Yes	No	Yes	R0 - R500 pm	No	none	No	one	Yes	Yes	No	25	No	30	No	2.3
15-19	Yes	No	Yes	R0 - R500 pm	No	none	No	none	.	.	.	.	.	18	No	1.4
15-19	No	Yes	Yes	R501 - R2,500 pm	No	none	No	one	No	Yes	No	18	No	7	No	1.2
15-19	No	Yes	Yes	R0 - R500 pm	No	none	No	one	Yes	Yes	No	21	No	39	No	1.8
20-24	Yes	Yes	Yes	.	No	none	No	one	Yes	Yes	No	25	No	12	No	1.9
15-19	No	Yes	Yes	R2,501 - R6,000 pm	No	none	No	none	.	.	.	.	.	49	No	2
15-19	No	Yes	Yes	R0 - R500 pm	No	none	No	one	No	Yes	No	21	No	29	No	1.2
15-19	No	No	Yes	greater than R6,000 pm	No	none	No	none	.	.	.	.	.	53	No	1.2
15-19	No	Yes	Yes	R501 - R2,500 pm	No	none	No	none	.	.	.	.	.	54	No	1.2
15-19	No	No	Yes	R2,501 - R6,000 pm	No	none	No	none	.	.	.	.	.	61	No	1.4
20-24	No	Yes	Yes	.	No	none	No	none	.	.	.	.	.	29	No	1.9
15-19	No	Yes	Yes	R501 - R2,500 pm	No	none	No	none	.	.	.	.	.	13	No	2.1
15-19	Yes	No	Yes	R0 - R500 pm	No	none	No	none	.	.	.	.	.	30	No	1.8
15-19	Yes	Yes	Yes	R2,501 - R6,000 pm	No	none	No	one	No	Yes	No	23	No	11	No	2
15-19	No	Yes	Yes	R501 - R2,500 pm	No	none	No	none	.	.	.	.	.	43	No	1.3
20-24	Yes	No	Yes	R501 - R2,500 pm	No	none	No	one	Yes	Yes	No	26	No	26	No	2
15-19	No	No	Yes	R501 - R2,500 pm	No	none	No	none	.	.	.	.	.	55	No	2.1
15-19	No	Yes	No	R0 - R500 pm	No	none	No	none	.	.	.	.	.	16	No	1.7
15-19	No	Yes	Yes	R501 - R2,500 pm	No	none	No	none	.	.	.	.	.	15	No	1.6
15-19	No	Yes	Yes	R2,501 - R6,000 pm	No	none	No	none	.	.	.	.	.	31	No	1.9
15-19	Yes	Yes	Yes	R501 - R2,500 pm	No	none	No	none	.	.	.	.	.	12	No	1.5
15-19	No	No	Yes	R501 - R2,500 pm	No	none	No	none	.	.	.	.	.	90	No	1.5
15-19	Yes	Yes	Yes	R501 - R2,500 pm	No	none	No	one	No	.	No	21	No	21	No	1
15-19	Yes	Yes	Yes	greater than R6,000 pm	No	none	No	none	.	.	.	.	.	10	No	1.3
20-24	Yes	Yes	No	R501 - R2,500 pm	No	none	No	one	No	Yes	No	25	No	11	No	1.9
15-19	No	No	Yes	R2,501 - R6,000 pm	No	none	No	none	.	.	.	.	.	47	No	1.7
15-19	No	Yes	Yes	R0 - R500 pm	No	none	No	one	No	Yes	No	19	No	13	No	1.6
15-19	No	No	Yes	R0 - R500 pm	No	none	No	one	Yes	.	No	18	No	47	No	1.5
15-19	No	Yes	Yes	R501 - R2,500 pm	No	none	No	one	Yes	No	No	19	No	15	No	1.9
15-19	No	Yes	Yes	R501 - R2,500 pm	No	none	No	one	No	No	No	28	No	16	Yes	0.9
15-19	No	No	Yes	R2,501 - R6,000 pm	No	none	No	none	.	.	.	.	.	80	No	1.5
15-19	Yes	Yes	Yes	R501 - R2,500 pm	No	none	No	none	.	.	.	.	.	13	No	1.9
15-19	Yes	Yes	Yes	R2,501 - R6,000 pm	No	none	No	one	No	Yes	No	24	No	23	No	1.5
15-19	Yes	No	Yes	R2,501 - R6,000 pm	No	none	No	none	.	.	.	.	.	18	No	1.2
20-24	Yes	Yes	Yes	R501 - R2,500 pm	No	none	No	none	.	.	.	.	.	10	No	1.5
15-19	Yes	Yes	Yes	R0 - R500 pm	No	none	No	one	Yes	Yes	Yes	22	No	12	No	1.6
15-19	No	Yes	Yes	R2,501 - R6,000 pm	No	none	No	none	.	.	.	.	.	9	No	1.8
15-19	No	No	Yes	R501 - R2,500 pm	No	none	No	one	No	Yes	No	25	No	77	No	1.5
20-24	No	Yes	No	.	Yes	none	.	none	.	.	.	.	.	16	No	1.8
15-19	Yes	Yes	Yes	.	No	none	No	one	Yes	No	No	27	No	15	No	1.7
15-19	No	No	Yes	R501 - R2,500 pm	No	none	No	none	.	.	.	.	.	21	No	1.2
20-24	Yes	No	Yes	R2,501 - R6,000 pm	No	none	No	none	.	.	.	.	.	91	No	1.3

15-19	No	No	Yes	R501 - R2,500 pm	No	none	No	none	.	.	.	.	.	106	No	1.3
15-19	No	Yes	Yes	R2,501 - R6,000 pm	No	none	No	none	.	.	.	.	.	32	No	1.4
20-24	Yes	No	Yes	R501 - R2,500 pm	Yes	none	No	one	No	No	No	28	No	26	No	1.8
15-19	No	Yes	Yes	R2,501 - R6,000 pm	No	none	No	none	.	.	.	.	.	49	No	1.3
15-19	Yes	No	Yes	R501 - R2,500 pm	No	none	No	one	No	Yes	No	29	No	91	No	2.1
15-19	Yes	No	No	R0 - R500 pm	No	none	.	none	.	.	.	.	.	33	No	1.8
15-19	No	No	Yes	R501 - R2,500 pm	No	none	No	none	.	.	.	.	.	12	No	1.3
15-19	No	Yes	No	R0 - R500 pm	No	none	No	one	No	No	No	23	No	9	No	1.8
20-24	Yes	Yes	Yes	R2,501 - R6,000 pm	No	none	No	none	.	.	.	.	.	12	No	2.2
15-19	No	Yes	Yes	R0 - R500 pm	No	none	No	none	.	.	.	.	.	20	No	1.7
20-24	Yes	No	Yes	greater than R6,000 pm	No	none	No	none	.	.	.	.	.	87	No	1.4
15-19	No	Yes	Yes	R501 - R2,500 pm	No	none	No	2 or more	No	No	No	28	No	18	No	2.6
15-19	No	Yes	Yes	R0 - R500 pm	No	none	No	one	No	.	No	20	No	27	No	1.2
15-19	Yes	Yes	Yes	R501 - R2,500 pm	No	none	No	one	Yes	Yes	No	25	No	14	No	1.6
15-19	Yes	Yes	Yes	R0 - R500 pm	No	none	No	none	.	.	.	.	.	9	No	1.5
15-19	No	Yes	Yes	greater than R6,000 pm	No	none	No	none	.	.	.	.	.	11	No	2
15-19	No	No	Yes	greater than R6,000 pm	No	none	No	one	No	No	No	19	No	102	No	1.3
20-24	Yes	Yes	Yes	R0 - R500 pm	No	none	No	none	.	.	.	.	.	46	No	1.3
20-24	Yes	Yes	No	R0 - R500 pm	No	none	No	2 or more	No	Yes	No	22	No	30	No	2.1
15-19	No	No	Yes	R0 - R500 pm	No	none	No	one	Yes	Yes	No	20	No	37	Yes	1.2
15-19	Yes	No	Yes	R2,501 - R6,000 pm	No	none	No	none	.	.	.	.	.	100	No	1.5
15-19	No	Yes	Yes	R501 - R2,500 pm	No	none	No	none	.	.	.	.	.	12	No	2.1
15-19	No	Yes	Yes	R501 - R2,500 pm	No	none	No	none	.	.	.	.	.	11	No	1.2
15-19	No	Yes	Yes	R2,501 - R6,000 pm	No	none	No	one	No	No	No	19	No	25	No	1.3
15-19	No	Yes	No	R0 - R500 pm	No	none	No	none	.	.	.	.	.	10	No	1.9
20-24	No	No	Yes	R501 - R2,500 pm	No	none	No	one	No	Yes	No	17	No	25	Yes	0.9
15-19	No	Yes	Yes	greater than R6,000 pm	No	none	No	none	.	.	.	.	.	31	No	1.2
15-19	No	No	Yes	R2,501 - R6,000 pm	No	none	No	none	.	.	.	.	.	14	No	1.3
20-24	No	No	Yes	R2,501 - R6,000 pm	No	none	No	none	.	.	.	.	.	71	No	1.2
15-19	No	Yes	Yes	R501 - R2,500 pm	No	none	No	one	No	Yes	No	21	No	90	No	2
15-19	No	No	Yes	R2,501 - R6,000 pm	No	none	No	none	.	.	.	.	.	32	No	1.8
15-19	No	No	Yes	R2,501 - R6,000 pm	No	none	No	none	.	.	.	.	.	106	No	1.9
15-19	No	Yes	Yes	R501 - R2,500 pm	No	none	No	one	No	Yes	No	19	No	15	Yes	0.8
15-19	Yes	Yes	Yes	R0 - R500 pm	No	none	No	one	No	Yes	No	33	No	12	No	1.6
15-19	No	No	Yes	R2,501 - R6,000 pm	No	none	No	one	Yes	Yes	No	20	Yes	34	No	2.3
15-19	No	Yes	Yes	.	No	none	No	none	.	.	.	.	.	11	No	2
15-19	No	Yes	Yes	R501 - R2,500 pm	No	none	No	one	No	Yes	No	21	No	34	No	1.6
20-24	No	No	Yes	R501 - R2,500 pm	No	none	No	none	.	.	.	.	.	27	No	1.4
15-19	No	Yes	Yes	R501 - R2,500 pm	No	none	No	one	No	Yes	No	20	No	27	No	1.3
15-19	No	Yes	Yes	R501 - R2,500 pm	No	none	No	none	.	.	.	.	.	24	No	1.7
15-19	No	Yes	Yes	.	No	none	No	none	.	.	.	.	.	65	No	1.3
15-19	No	No	Yes	R501 - R2,500 pm	No	none	No	one	Yes	Yes	No	17	No	28	Yes	0.9

15-19	Yes	Yes	Yes	R501 - R2,500 pm	No	none	No	one	No	Yes	No	25	No	12	No	1.8
15-19	No	No	Yes	R501 - R2,500 pm	No	none	No	one	Yes	Yes	No	24	Yes	35	No	2.3
15-19	No	Yes	Yes	R501 - R2,500 pm	No	none	No	none	.	.	.	.	.	16	No	1.3
15-19	No	Yes	Yes	R2,501 - R6,000 pm	No	none	No	none	.	.	.	.	.	18	No	1.9
15-19	Yes	Yes	No	R501 - R2,500 pm	No	none	No	one	No	No	No	23	No	11	No	1.9
20-24	Yes	No	Yes	R501 - R2,500 pm	No	none	No	none	.	.	.	.	.	102	No	1.8
15-19	Yes	Yes	Yes	R2,501 - R6,000 pm	No	none	No	none	.	.	.	.	.	26	No	1
15-19	Yes	No	Yes	R0 - R500 pm	Yes	none	No	one	Yes	Yes	No	23	No	26	No	1.8
20-24	Yes	Yes	Yes	.	No	none	No	one	No	Yes	No	23	No	10	No	2
15-19	No	Yes	Yes	R501 - R2,500 pm	No	none	No	one	Yes	.	No	20	No	12	No	1.3
20-24	Yes	Yes	Yes	R501 - R2,500 pm	No	none	No	none	.	.	.	.	.	22	No	1.3
15-19	No	Yes	No	.	No	none	No	none	.	.	.	.	.	12	No	1.6
15-19	No	Yes	Yes	R2,501 - R6,000 pm	No	none	No	one	Yes	Yes	No	19	No	12	No	2.2
15-19	No	Yes	No	R501 - R2,500 pm	No	none	No	none	.	.	.	.	.	47	No	1.2
15-19	No	Yes	Yes	R2,501 - R6,000 pm	No	none	No	2 or more	No	No	No	35	Yes	22	No	1.6
15-19	No	Yes	Yes	R0 - R500 pm	No	none	.	one	No	No	No	23	No	35	No	2.1
20-24	No	No	Yes	R501 - R2,500 pm	No	none	Yes	one	Yes	Yes	No	29	No	40	No	2.3
15-19	No	No	Yes	R0 - R500 pm	No	none	No	none	.	.	.	.	.	66	No	1.4
20-24	Yes	Yes	Yes	R0 - R500 pm	No	none	No	one	Yes	.	No	29	No	12	No	1.6
15-19	No	Yes	Yes	R501 - R2,500 pm	No	none	No	2 or more	No	Yes	No	19	No	11	No	2
15-19	No	Yes	Yes	greater than R6,000 pm	No	none	No	none	.	.	.	.	.	17	No	1.3
20-24	Yes	Yes	Yes	R0 - R500 pm	No	none	No	none	.	.	.	.	.	14	No	1.3
15-19	Yes	Yes	Yes	.	No	none	No	one	Yes	Yes	No	21	No	21	No	2.2
15-19	Yes	No	Yes	R501 - R2,500 pm	No	none	No	one	No	Yes	No	25	No	102	No	1.5
15-19	No	Yes	No	R2,501 - R6,000 pm	Yes	none	No	none	.	.	.	.	.	13	No	1.3
15-19	No	Yes	Yes	R0 - R500 pm	No	none	No	none	.	.	.	.	.	36	No	1.5
15-19	No	Yes	Yes	R0 - R500 pm	No	none	No	one	No	Yes	No	27	No	15	No	2.4
20-24	Yes	No	Yes	greater than R6,000 pm	No	none	No	none	.	.	.	.	.	87	No	1.4
15-19	Yes	Yes	Yes	R2,501 - R6,000 pm	No	none	No	none	.	.	.	.	.	10	No	2.1
15-19	Yes	Yes	Yes	R0 - R500 pm	No	none	No	none	.	.	.	.	.	35	No	1.3
15-19	No	No	Yes	R2,501 - R6,000 pm	No	none	No	one	No	No	No	28	No	21	No	1.4
15-19	No	No	Yes	R2,501 - R6,000 pm	No	none	No	none	.	.	.	.	.	21	No	1.1
15-19	No	No	Yes	R501 - R2,500 pm	No	none	No	none	.	.	.	.	.	21	No	1.1
15-19	No	No	Yes	R2,501 - R6,000 pm	No	none	No	none	.	.	.	.	.	31	No	1.2
15-19	Yes	Yes	Yes	R2,501 - R6,000 pm	Yes	none	No	none	.	.	.	.	.	33	No	1.3
15-19	No	Yes	Yes	greater than R6,000 pm	Yes	none	No	none	.	.	.	.	.	41	No	1.7
15-19	Yes	Yes	Yes	R2,501 - R6,000 pm	No	none	No	one	No	Yes	No	23	No	24	No	1.9
15-19	No	Yes	Yes	R2,501 - R6,000 pm	No	none	No	none	.	.	.	.	.	52	No	1.7
15-19	No	Yes	Yes	R2,501 - R6,000 pm	No	none	No	none	.	.	.	.	.	24	No	1.6
15-19	No	Yes	Yes	greater than R6,000 pm	No	none	No	none	.	.	.	.	.	48	No	1.3
15-19	No	Yes	Yes	R2,501 - R6,000 pm	No	none	No	none	.	.	.	.	.	17	No	1.6
15-19	No	Yes	Yes	R501 - R2,500 pm	No	none	No	one	No	No	No	20	No	37	No	1.3

15-19	No	Yes	Yes	R2,501 - R6,000 pm	No	none	No	none	.	.	.	.	.	24	No	1.3
15-19	No	Yes	Yes	R2,501 - R6,000 pm	No	none	No	none	.	.	.	.	.	29	No	1.7
15-19	Yes	Yes	Yes	R501 - R2,500 pm	Yes	none	No	one	Yes	Yes	No	23	No	27	No	1.7
15-19	No	Yes	Yes	R2,501 - R6,000 pm	No	none	No	none	.	.	.	.	.	19	No	1.7
15-19	Yes	Yes	Yes	R501 - R2,500 pm	No	none	No	none	.	.	.	.	.	53	No	1.9
20-24	No	Yes	Yes	R2,501 - R6,000 pm	No	none	No	none	.	.	.	.	.	58	No	1.1
15-19	No	Yes	Yes	R2,501 - R6,000 pm	No	none	No	none	.	.	.	.	.	32	No	1.4
15-19	No	Yes	Yes	R501 - R2,500 pm	No	none	No	none	.	.	.	.	.	25	No	1.2
20-24	Yes	Yes	Yes	R501 - R2,500 pm	No	none	No	one	Yes	Yes	No	26	No	29	No	1.2
15-19	No	Yes	Yes	R501 - R2,500 pm	No	none	No	none	.	.	.	.	.	25	No	1.3
15-19	Yes	Yes	Yes	greater than R6,000 pm	No	none	No	one	No	Yes	No	29	No	27	No	1.4
15-19	No	Yes	Yes	R2,501 - R6,000 pm	No	none	No	one	No	No	No	22	No	9	No	1.5
15-19	No	Yes	No	R501 - R2,500 pm	No	none	No	none	.	.	.	.	.	9	No	1.4
15-19	Yes	Yes	Yes	greater than R6,000 pm	No	none	No	none	.	.	.	.	.	17	No	1.3
15-19	No	Yes	Yes	R2,501 - R6,000 pm	No	none	No	none	.	.	.	.	.	10	No	1.6
15-19	No	Yes	Yes	greater than R6,000 pm	No	none	No	none	.	.	.	.	.	45	No	2
15-19	No	Yes	Yes	R501 - R2,500 pm	No	none	No	none	.	.	.	.	.	19	No	1.7
15-19	No	Yes	Yes	R2,501 - R6,000 pm	No	none	No	none	.	.	.	.	.	23	No	1.2
15-19	Yes	Yes	Yes	R501 - R2,500 pm	No	none	No	none	.	.	.	.	.	10	No	1.5
15-19	No	Yes	Yes	R501 - R2,500 pm	No	none	No	one	No	Yes	No	19	No	48	No	1.4
15-19	No	Yes	Yes	greater than R6,000 pm	No	none	No	one	No	Yes	No	23	No	29	No	1.5
15-19	No	Yes	Yes	R2,501 - R6,000 pm	No	none	No	one	No	Yes	No	18	No	20	No	1.4
15-19	No	No	No	R501 - R2,500 pm	No	none	No	one	Yes	Yes	No	19	No	19	No	1.8
15-19	No	No	Yes	R501 - R2,500 pm	No	none	No	none	.	.	.	.	.	10	No	2.1
15-19	No	No	Yes	R501 - R2,500 pm	No	none	No	one	No	Yes	No	19	No	20	No	2.1
15-19	No	No	Yes	greater than R6,000 pm	No	none	No	none	.	.	.	.	.	28	No	2.1
15-19	No	No	Yes	greater than R6,000 pm	No	none	No	none	.	.	.	.	.	9	No	1.8
15-19	Yes	No	Yes	R501 - R2,500 pm	No	none	No	none	.	.	.	.	.	9	No	1.8
15-19	No	No	No	R501 - R2,500 pm	No	none	No	one	No	Yes	No	25	No	9	No	1.8
15-19	No	No	Yes	R501 - R2,500 pm	No	none	No	none	.	.	.	.	.	47	No	1.8
15-19	No	No	Yes	R501 - R2,500 pm	No	none	No	none	.	.	.	.	.	17	No	1.8
15-19	No	No	Yes	R501 - R2,500 pm	No	none	No	none	.	.	.	.	.	40	No	1.8
15-19	Yes	No	Yes	R2,501 - R6,000 pm	No	none	No	one	No	Yes	No	29	No	13	No	2
20-24	Yes	No	Yes	R2,501 - R6,000 pm	No	none	No	one	No	Yes	No	36	No	42	No	1.8
15-19	No	No	Yes	greater than R6,000 pm	No	none	No	one	No	.	No	25	No	36	No	1.8
20-24	Yes	No	Yes	R2,501 - R6,000 pm	No	none	No	none	.	.	.	.	.	40	No	1.8
15-19	No	No	Yes	R501 - R2,500 pm	No	none	No	none	.	.	.	.	.	9	No	1.8
15-19	Yes	No	Yes	R501 - R2,500 pm	No	none	No	none	.	.	.	.	.	27	No	1.7
20-24	No	No	Yes	R0 - R500 pm	No	none	No	none	.	.	.	.	.	50	No	1.8

15-19	No	No	Yes	greater than R6,000 pm	No	none	No	none	.	.	.	.	.	24	No	1.9
15-19	No	No	Yes	R501 - R2,500 pm	No	none	No	one	No	Yes	No	19	No	37	No	1.5
15-19	No	No	Yes	R2,501 - R6,000 pm	No	none	No	none	.	.	.	.	.	37	Yes	0.6
15-19	Yes	No	Yes	R2,501 - R6,000 pm	No	none	No	none	.	.	.	.	.	10	No	1.2
15-19	No	No	Yes	R2,501 - R6,000 pm	No	none	No	none	.	.	.	.	.	18	No	1.3
15-19	No	No	Yes	R501 - R2,500 pm	No	none	No	none	.	.	.	.	.	31	No	1.3
15-19	No	No	Yes	R2,501 - R6,000 pm	No	none	No	none	.	.	.	.	.	22	No	1.3
15-19	Yes	No	Yes	R501 - R2,500 pm	No	none	No	none	.	.	.	.	.	44	No	1.3
15-19	Yes	No	Yes	greater than R6,000 pm	No	none	No	one	No	Yes	No	30	No	11	No	1.5
15-19	No	No	No	R2,501 - R6,000 pm	Yes	none	No	none	.	.	.	.	.	46	No	1.7
15-19	No	No	Yes	R2,501 - R6,000 pm	No	none	No	none	.	.	.	.	.	53	No	1.7
20-24	No	No	Yes	R501 - R2,500 pm	No	none	No	one	No	Yes	No	25	No	39	No	1.7
15-19	No	No	Yes	R501 - R2,500 pm	No	none	No	none	.	.	.	.	.	53	No	1.7
15-19	No	No	Yes	R2,501 - R6,000 pm	No	none	No	one	Yes	.	No	19	No	24	Yes	0.9
15-19	No	No	Yes	R501 - R2,500 pm	No	none	No	none	.	.	.	.	.	25	No	1.7
20-24	Yes	No	No	R501 - R2,500 pm	No	none	No	one	No	Yes	No	26	No	60	No	2.1
20-24	No	No	Yes	R2,501 - R6,000 pm	No	none	No	none	.	.	.	.	.	38	No	1.2
15-19	No	No	Yes	R501 - R2,500 pm	No	none	No	one	No	No	No	17	No	34	No	1.1
15-19	No	No	Yes	R2,501 - R6,000 pm	No	none	No	none	.	.	.	.	.	53	No	1.2
15-19	No	No	Yes	R2,501 - R6,000 pm	No	none	No	none	.	.	.	.	.	53	No	1.1
15-19	No	No	Yes	R501 - R2,500 pm	No	none	No	none	.	.	.	.	.	37	No	1.5
15-19	No	No	Yes	R501 - R2,500 pm	No	none	No	none	.	.	.	.	.	25	No	1.7
20-24	No	No	Yes	R501 - R2,500 pm	Yes	none	No	none	.	.	.	.	.	27	No	1.4
15-19	Yes	No	Yes	R501 - R2,500 pm	Yes	none	No	one	No	Yes	No	25	No	12	No	1.5
15-19	No	No	Yes	R2,501 - R6,000 pm	No	none	No	none	.	.	.	.	.	41	No	1.8
15-19	No	No	Yes	R2,501 - R6,000 pm	No	none	No	none	.	.	.	.	.	16	No	1.7
15-19	Yes	No	Yes	R501 - R2,500 pm	No	none	No	none	.	.	.	.	.	16	No	2.1
15-19	No	No	Yes	R501 - R2,500 pm	No	none	No	none	.	.	.	.	.	14	No	1.7
15-19	Yes	No	Yes	R0 - R500 pm	No	none	No	none	.	.	.	.	.	40	No	1.7
15-19	No	No	Yes	R0 - R500 pm	No	none	No	none	.	.	.	.	.	26	No	1.7
15-19	No	No	Yes	R0 - R500 pm	No	none	No	none	.	.	.	.	.	24	No	1.6
15-19	No	No	Yes	R501 - R2,500 pm	No	none	No	one	No	Yes	No	25	No	11	No	1.5
15-19	Yes	No	Yes	R2,501 - R6,000 pm	No	none	No	one	No	Yes	No	24	No	53	No	1.2
15-19	No	No	Yes	R501 - R2,500 pm	No	none	No	none	.	.	.	.	.	32	No	1.7
20-24	No	No	Yes	R501 - R2,500 pm	No	none	No	one	No	Yes	No	20	No	30	No	2.1
15-19	No	No	Yes	R501 - R2,500 pm	No	none	No	none	.	.	.	.	.	29	No	2.1
20-24	Yes	No	Yes	greater than R6,000 pm	No	none	No	none	.	.	.	.	.	51	No	1.4
15-19	No	No	Yes	R501 - R2,500 pm	No	none	No	none	.	.	.	.	.	16	No	1.4
20-24	No	No	Yes	R2,501 - R6,000 pm	No	none	No	none	.	.	.	.	.	43	No	1.5
15-19	No	No	Yes	R501 - R2,500 pm	No	none	No	none	.	.	.	.	.	23	No	1.6
15-19	No	No	Yes	R2,501 - R6,000 pm	No	none	No	none	.	.	.	.	.	38	No	1.6
15-19	No	No	Yes	R2,501 - R6,000 pm	Yes	none	No	none	.	.	.	.	.	42	No	1.8
15-19	No	No	Yes	R2,501 - R6,000 pm	No	none	No	none	.	.	.	.	.	17	No	1.8

15-19	No	No	Yes	greater than R6,000 pm	No	none	No	none	.	.	.	.	.	26	No	1.6
20-24	Yes	No	Yes	R2,501 - R6,000 pm	No	none	No	none	.	.	.	.	.	15	No	1.4
15-19	Yes	No	Yes	R2,501 - R6,000 pm	No	none	No	none	.	.	.	.	.	37	No	1.4
15-19	No	No	Yes	R501 - R2,500 pm	No	none	No	none	.	.	.	.	.	17	Yes	0.7
15-19	No	Yes	Yes	R501 - R2,500 pm	No	none	No	one	Yes	Yes	No	18	Yes	15	No	1.3
15-19	Yes	Yes	Yes	R2,501 - R6,000 pm	Yes	none	No	none	.	.	.	.	.	52	No	1.3
20-24	No	Yes	Yes	R2,501 - R6,000 pm	No	none	No	one	Yes	Yes	No	24	No	17	No	1.3
15-19	No	Yes	Yes	greater than R6,000 pm	No	none	No	none	.	.	.	.	.	53	No	1.3
15-19	No	Yes	Yes	R2,501 - R6,000 pm	No	none	No	none	.	.	.	.	.	27	No	1.3
20-24	Yes	Yes	Yes	R2,501 - R6,000 pm	No	none	No	none	.	.	.	.	.	28	No	1.3
20-24	Yes	Yes	Yes	R2,501 - R6,000 pm	No	none	Yes	one	No	Yes	No	25	No	45	No	1.3
15-19	No	Yes	Yes	R2,501 - R6,000 pm	No	none	No	none	.	.	.	.	.	52	No	1.2
15-19	No	Yes	Yes	R501 - R2,500 pm	No	none	No	none	.	.	.	.	.	20	No	1.5
15-19	No	Yes	Yes	R2,501 - R6,000 pm	No	none	No	one	No	.	No	20	Yes	19	No	1.5
15-19	No	Yes	Yes	R501 - R2,500 pm	No	none	No	none	.	.	.	.	.	21	No	1.3
15-19	No	Yes	No	R501 - R2,500 pm	No	none	No	none	.	.	.	.	.	33	No	1.3
15-19	No	Yes	Yes	R501 - R2,500 pm	No	none	No	none	.	.	.	.	.	42	No	1.3
15-19	No	Yes	Yes	R501 - R2,500 pm	No	none	No	none	.	.	.	.	.	42	Yes	0.7
15-19	No	Yes	No	R2,501 - R6,000 pm	No	none	No	none	.	.	.	.	.	33	Yes	0.7
15-19	No	Yes	Yes	R501 - R2,500 pm	Yes	none	No	none	.	.	.	.	.	11	No	1.3
15-19	No	Yes	Yes	R2,501 - R6,000 pm	No	none	No	none	.	.	.	.	.	23	No	1.4
15-19	Yes	Yes	Yes	R2,501 - R6,000 pm	No	none	No	one	No	Yes	No	23	No	21	No	1.3
15-19	No	Yes	Yes	R2,501 - R6,000 pm	No	none	No	none	.	.	.	.	.	15	No	1.4
20-24	Yes	Yes	No	R501 - R2,500 pm	No	none	No	none	.	.	.	.	.	38	No	1.3
15-19	No	Yes	Yes	R0 - R500 pm	No	none	No	none	.	.	.	.	.	23	No	1.3
15-19	No	Yes	Yes	R501 - R2,500 pm	No	none	No	none	.	.	.	.	.	23	No	1.3
15-19	No	Yes	Yes	R501 - R2,500 pm	No	none	No	none	.	.	.	.	.	33	No	1.3
15-19	No	Yes	Yes	R501 - R2,500 pm	No	none	No	none	.	.	.	.	.	23	No	1.8
15-19	No	Yes	Yes	R2,501 - R6,000 pm	No	none	No	none	.	.	.	.	.	22	No	1.3
20-24	Yes	Yes	Yes	R501 - R2,500 pm	No	none	No	one	No	Yes	No	26	No	48	No	1.3
20-24	Yes	Yes	No	R501 - R2,500 pm	No	none	No	none	.	.	.	.	.	48	No	1.6
15-19	No	Yes	Yes	R501 - R2,500 pm	No	none	No	none	.	.	.	.	.	9	No	1.3
15-19	No	Yes	Yes	greater than R6,000 pm	No	none	No	none	.	.	.	.	.	9	No	1.2
15-19	Yes	Yes	Yes	greater than R6,000 pm	No	none	No	one	Yes	Yes	No	19	No	24	No	1.3
15-19	No	Yes	Yes	R501 - R2,500 pm	No	none	No	none	.	.	.	.	.	17	No	1.3
15-19	No	Yes	Yes	R501 - R2,500 pm	No	none	No	one	Yes	.	No	24	No	9	No	1.2
20-24	Yes	Yes	Yes	R501 - R2,500 pm	No	none	No	one	No	Yes	No	23	Yes	34	No	1.4
15-19	No	Yes	Yes	R2,501 - R6,000 pm	No	none	No	none	.	.	.	.	.	46	No	1.4
15-19	No	Yes	Yes	R2,501 - R6,000 pm	No	none	No	none	.	.	.	.	.	46	No	1.4
15-19	No	Yes	Yes	R2,501 - R6,000 pm	No	none	No	one	No	.	No	19	No	32	No	1.3
15-19	Yes	Yes	Yes	R2,501 - R6,000 pm	No	none	No	none	.	.	.	.	.	46	No	1.4
15-19	No	Yes	Yes	R2,501 - R6,000 pm	No	none	No	none	.	.	.	.	.	18	No	1.3

15-19	No	Yes	Yes	R2,501 - R6,000 pm	No	none	No	none	.	.	.	.	.	28	No	1.4
15-19	No	Yes	Yes	R501 - R2,500 pm	No	none	No	none	.	.	.	.	.	20	No	1.4
15-19	No	Yes	Yes	R501 - R2,500 pm	No	none	No	none	.	.	.	.	.	37	No	1.4
15-19	Yes	Yes	Yes	R2,501 - R6,000 pm	No	none	No	one	Yes	Yes	No	20	No	16	Yes	0.7
15-19	Yes	Yes	Yes	R2,501 - R6,000 pm	No	none	No	one	Yes	Yes	No	21	No	15	No	1.8
15-19	Yes	Yes	Yes	R2,501 - R6,000 pm	No	none	No	one	No	Yes	No	20	No	52	No	2
15-19	No	Yes	Yes	greater than R6,000 pm	No	none	No	none	.	.	.	.	.	27	No	1.4
15-19	No	Yes	Yes	R2,501 - R6,000 pm	No	none	No	none	.	.	.	.	.	29	No	1.4
15-19	No	Yes	Yes	R2,501 - R6,000 pm	No	none	No	none	.	.	.	.	.	28	No	1.4
15-19	No	Yes	Yes	R501 - R2,500 pm	No	none	No	one	No	Yes	No	21	No	28	No	1.5
15-19	No	Yes	Yes	R501 - R2,500 pm	No	none	No	none	.	.	.	.	.	9	No	1.5
15-19	No	Yes	Yes	R2,501 - R6,000 pm	No	none	No	none	.	.	.	.	.	31	No	1.3
15-19	No	Yes	Yes	R501 - R2,500 pm	No	none	No	none	.	.	.	.	.	10	No	1.3
15-19	No	Yes	Yes	R501 - R2,500 pm	No	none	No	none	.	.	.	.	.	10	No	1.3
15-19	No	Yes	Yes	R501 - R2,500 pm	No	none	No	none	.	.	.	.	.	10	No	1.6
20-24	Yes	Yes	Yes	R2,501 - R6,000 pm	No	none	No	none	.	.	.	.	.	12	No	1.3
20-24	Yes	Yes	Yes	R501 - R2,500 pm	No	none	No	one	Yes	Yes	No	22	No	12	No	2
15-19	No	Yes	Yes	R501 - R2,500 pm	No	none	No	one	No	Yes	No	21	No	30	No	1.6
15-19	No	No	Yes	R501 - R2,500 pm	No	none	No	none	.	.	.	.	.	24	No	1.5
15-19	No	No	Yes	R501 - R2,500 pm	No	none	No	none	.	.	.	.	.	38	No	2
15-19	No	No	Yes	R501 - R2,500 pm	No	none	No	one	No	Yes	No	28	No	48	No	1.5
15-19	Yes	No	Yes	greater than R6,000 pm	No	none	No	none	.	.	.	.	.	47	No	1.5
15-19	No	No	Yes	R501 - R2,500 pm	No	none	No	none	.	.	.	.	.	25	No	1.5
20-24	No	No	Yes	R2,501 - R6,000 pm	No	none	No	none	.	.	.	.	.	46	No	1.2
15-19	No	No	Yes	R2,501 - R6,000 pm	No	none	No	none	.	.	.	.	.	52	No	1.1
15-19	Yes	No	Yes	R501 - R2,500 pm	No	none	No	none	.	.	.	.	.	26	No	1.6
20-24	Yes	No	Yes	R501 - R2,500 pm	No	none	No	none	.	.	.	.	.	29	No	1.7
15-19	No	No	Yes	R501 - R2,500 pm	No	none	No	none	.	.	.	.	.	26	No	1.5
15-19	No	No	Yes	R501 - R2,500 pm	No	none	No	none	.	.	.	.	.	39	No	1.5
15-19	No	No	Yes	R501 - R2,500 pm	No	none	No	none	.	.	.	.	.	13	No	1.2
15-19	No	Yes	Yes	R501 - R2,500 pm	No	none	No	none	.	.	.	.	.	10	No	1.6
15-19	No	Yes	Yes	R501 - R2,500 pm	No	none	No	none	.	.	.	.	.	19	No	1.6
15-19	No	Yes	Yes	R2,501 - R6,000 pm	No	none	No	one	No	.	No	20	Yes	10	No	1.7
15-19	No	Yes	Yes	R501 - R2,500 pm	No	none	No	none	.	.	.	.	.	32	No	1.4
15-19	No	Yes	Yes	R2,501 - R6,000 pm	No	none	No	one	No	No	No	19	Yes	11	No	1.5
15-19	No	Yes	Yes	R501 - R2,500 pm	No	none	Yes	one	Yes	No	No	22	No	11	No	1.4
15-19	No	Yes	Yes	R2,501 - R6,000 pm	No	none	No	none	.	.	.	.	.	32	No	1.4
15-19	No	Yes	Yes	greater than R6,000 pm	No	none	No	none	.	.	.	.	.	50	No	1.1
15-19	No	Yes	Yes	R2,501 - R6,000 pm	No	none	No	none	.	.	.	.	.	53	No	1.4
15-19	No	Yes	Yes	R501 - R2,500 pm	No	none	No	none	.	.	.	.	.	9	No	1.3
15-19	Yes	Yes	Yes	R2,501 - R6,000 pm	No	none	No	one	Yes	Yes	No	24	No	26	No	1.3
15-19	No	Yes	Yes	R2,501 - R6,000 pm	No	none	No	one	Yes	Yes	No	25	No	52	No	1.4
20-24	Yes	Yes	Yes	R501 - R2,500 pm	No	none	No	one	No	Yes	No	23	No	12	No	1.4

15-19	Yes	Yes	Yes	R2,501 - R6,000 pm	No	none	No	none	.	.	.	.	.	32	No	1.4
15-19	No	Yes	Yes	R2,501 - R6,000 pm	No	none	No	none	.	.	.	.	.	43	No	1.4
15-19	Yes	No	Yes	greater than R6,000 pm	No	none	No	none	.	.	.	.	.	30	No	1.3
20-24	No	No	Yes	R501 - R2,500 pm	Yes	none	No	one	No	.	No	21	No	34	Yes	0.6
15-19	No	No	Yes	R501 - R2,500 pm	No	none	No	one	No	Yes	No	20	No	20	No	1.4
20-24	Yes	Yes	Yes	R2,501 - R6,000 pm	No	none	No	none	.	.	.	.	.	37	No	1.4
15-19	No	Yes	Yes	R2,501 - R6,000 pm	No	none	No	none	.	.	.	.	.	28	No	1.4
15-19	No	Yes	Yes	R0 - R500 pm	No	none	No	none	.	.	.	.	.	40	No	1.4
15-19	Yes	Yes	Yes	R501 - R2,500 pm	No	none	No	none	.	.	.	.	.	14	No	1.4
15-19	No	Yes	Yes	R2,501 - R6,000 pm	No	none	No	none	.	.	.	.	.	14	No	1.4
15-19	No	Yes	No	R501 - R2,500 pm	No	none	No	one	No	Yes	No	17	No	28	No	1.4
15-19	No	Yes	Yes	R2,501 - R6,000 pm	Yes	none	No	one	Yes	.	No	19	No	41	No	1.2
20-24	Yes	Yes	Yes	R501 - R2,500 pm	No	none	No	one	No	Yes	No	19	No	56	No	1.3
15-19	Yes	Yes	Yes	R501 - R2,500 pm	No	none	No	none	.	.	.	.	.	21	No	1.1
15-19	No	No	Yes	R2,501 - R6,000 pm	No	none	No	none	.	.	.	.	.	52	No	1.4
15-19	No	No	Yes	R501 - R2,500 pm	Yes	none	No	one	No	No	No	22	No	53	No	1.3
15-19	No	No	Yes	R501 - R2,500 pm	No	none	No	none	.	.	.	.	.	11	No	1.5
15-19	No	No	Yes	R2,501 - R6,000 pm	No	none	No	none	.	.	.	.	.	44	No	1.2
15-19	No	No	Yes	R2,501 - R6,000 pm	No	none	No	none	.	.	.	.	.	32	No	1.3
15-19	No	No	Yes	greater than R6,000 pm	No	none	No	none	.	.	.	.	.	33	No	1.2
20-24	Yes	No	Yes	R501 - R2,500 pm	No	none	No	none	.	.	.	.	.	33	No	1.3
15-19	No	No	Yes	R2,501 - R6,000 pm	No	none	No	none	.	.	.	.	.	12	No	1.9
20-24	Yes	No	Yes	R501 - R2,500 pm	No	none	No	none	.	.	.	.	.	59	No	1.7
15-19	No	No	Yes	R2,501 - R6,000 pm	No	none	No	none	.	.	.	.	.	41	No	1.3
15-19	No	No	Yes	R2,501 - R6,000 pm	No	none	No	one	No	Yes	No	19	No	53	No	1.2
15-19	No	No	Yes	R2,501 - R6,000 pm	No	none	No	none	.	.	.	.	.	14	No	1.2
15-19	No	No	Yes	R501 - R2,500 pm	No	none	No	one	No	Yes	No	22	No	29	No	1.2
15-19	No	No	Yes	R501 - R2,500 pm	No	none	No	none	.	.	.	.	.	28	No	1.2
15-19	No	No	Yes	R2,501 - R6,000 pm	No	none	No	one	No	Yes	No	21	No	52	No	1.7
15-19	Yes	No	Yes	R2,501 - R6,000 pm	No	none	No	one	No	No	No	21	No	50	No	1.7
15-19	No	No	Yes	R501 - R2,500 pm	No	none	No	none	.	.	.	.	.	24	No	1.7
20-24	No	No	Yes	R2,501 - R6,000 pm	No	none	No	none	.	.	.	.	.	57	No	1.7
20-24	Yes	No	Yes	R501 - R2,500 pm	No	none	No	none	.	.	.	.	.	60	No	1.6
20-24	Yes	No	Yes	R2,501 - R6,000 pm	No	none	No	none	.	.	.	.	.	59	No	1.7
15-19	No	Yes	Yes	R501 - R2,500 pm	No	none	No	none	.	.	.	.	.	23	No	1.1
15-19	No	Yes	Yes	R2,501 - R6,000 pm	No	none	No	one	Yes	Yes	No	19	No	52	No	1.1
15-19	No	Yes	No	R501 - R2,500 pm	No	none	No	none	.	.	.	.	.	17	No	1.3
15-19	No	Yes	No	greater than R6,000 pm	No	none	No	one	No	Yes	No	24	No	26	No	1.3
15-19	Yes	Yes	Yes	greater than R6,000 pm	No	none	No	one	No	Yes	No	24	No	10	No	1.3
15-19	No	Yes	Yes	R501 - R2,500 pm	No	none	No	none	.	.	.	.	.	9	No	1.3
15-19	No	Yes	Yes	R501 - R2,500 pm	No	none	No	none	.	.	.	.	.	21	No	1.4
15-19	No	Yes	Yes	R2,501 - R6,000 pm	No	none	No	none	.	.	.	.	.	32	No	1.4

20-24	Yes	Yes	Yes	R501 - R2,500 pm	No	none	No	one	No	Yes	No	21	No	22	No	1.4
15-19	Yes	Yes	Yes	R2,501 - R6,000 pm	No	none	No	none	.	.	.	.	.	45	No	1.3
15-19	No	Yes	Yes	R2,501 - R6,000 pm	No	none	No	none	.	.	.	.	.	14	No	1.3
15-19	No	Yes	Yes	R2,501 - R6,000 pm	No	none	No	none	.	.	.	.	.	29	No	1.1
15-19	Yes	Yes	Yes	R2,501 - R6,000 pm	No	none	Yes	one	No	Yes	No	23	No	29	No	1.3
15-19	Yes	Yes	Yes	R2,501 - R6,000 pm	No	none	No	none	.	.	.	.	.	29	No	1.3
15-19	Yes	Yes	Yes	R501 - R2,500 pm	No	none	No	one	Yes	Yes	No	20	No	42	No	1.3
20-24	No	Yes	Yes	R501 - R2,500 pm	No	none	No	one	Yes	Yes	No	26	No	23	No	1.3
15-19	No	Yes	Yes	R2,501 - R6,000 pm	No	none	No	none	.	.	.	.	.	32	No	1.3
15-19	No	Yes	Yes	R2,501 - R6,000 pm	No	none	No	none	.	.	.	.	.	11	No	1.3
15-19	No	Yes	Yes	R501 - R2,500 pm	No	none	No	one	No	Yes	No	23	No	22	No	1.4
20-24	Yes	Yes	Yes	R2,501 - R6,000 pm	No	none	No	one	No	.	No	24	No	46	No	1.3
15-19	No	Yes	Yes	R2,501 - R6,000 pm	No	none	No	none	.	.	.	.	.	30	No	1.3
15-19	Yes	Yes	Yes	R501 - R2,500 pm	No	none	No	none	.	.	.	.	.	42	No	1.2
15-19	No	Yes	Yes	R501 - R2,500 pm	No	none	No	one	No	Yes	No	23	No	22	No	1.2
15-19	Yes	Yes	Yes	greater than R6,000 pm	No	none	No	one	No	Yes	No	20	No	38	No	1.4
15-19	No	Yes	Yes	R2,501 - R6,000 pm	No	none	No	none	.	.	.	.	.	15	No	1.1
15-19	No	Yes	Yes	R501 - R2,500 pm	No	none	No	none	.	.	.	.	.	9	No	1.3
15-19	No	Yes	Yes	R2,501 - R6,000 pm	No	none	No	none	.	.	.	.	.	33	No	1.1
20-24	Yes	Yes	Yes	R2,501 - R6,000 pm	No	none	No	one	No	Yes	No	24	No	10	No	1.2
15-19	No	Yes	Yes	R2,501 - R6,000 pm	No	none	No	one	Yes	Yes	No	20	No	15	No	1.3
15-19	No	Yes	Yes	greater than R6,000 pm	Yes	none	No	one	No	Yes	No	19	No	9	No	1.2
15-19	No	Yes	Yes	greater than R6,000 pm	No	none	No	one	Yes	Yes	No	21	No	17	No	1.7
15-19	No	Yes	Yes	greater than R6,000 pm	No	none	No	none	.	.	.	.	.	49	No	1.2
15-19	No	Yes	Yes	R501 - R2,500 pm	No	none	No	one	Yes	.	No	19	No	22	No	1.8
15-19	No	Yes	Yes	R501 - R2,500 pm	No	none	No	none	.	.	.	.	.	11	No	1.5
15-19	Yes	Yes	Yes	R501 - R2,500 pm	No	none	No	none	.	.	.	.	.	11	No	1.8
15-19	No	Yes	Yes	greater than R6,000 pm	No	none	No	none	.	.	.	.	.	28	No	1.4
15-19	Yes	Yes	Yes	R501 - R2,500 pm	Yes	none	No	2 or more	No	Yes	No	24	No	28	No	1.4
15-19	Yes	Yes	Yes	greater than R6,000 pm	No	none	No	one	No	No	No	19	No	21	No	1.2
15-19	No	Yes	Yes	R501 - R2,500 pm	No	none	No	one	No	Yes	No	25	No	30	No	1.3
15-19	No	Yes	Yes	R501 - R2,500 pm	No	none	No	none	.	.	.	.	.	43	No	1.5
15-19	Yes	Yes	Yes	greater than R6,000 pm	No	none	No	none	.	.	.	.	.	31	No	1.5
15-19	No	Yes	Yes	R0 - R500 pm	No	none	No	none	.	.	.	.	.	19	No	1.5
15-19	No	Yes	Yes	R501 - R2,500 pm	No	none	No	none	.	.	.	.	.	10	No	1.5
15-19	No	Yes	Yes	R501 - R2,500 pm	No	none	No	none	.	.	.	.	.	53	No	1.8
15-19	No	Yes	Yes	R501 - R2,500 pm	No	none	No	none	.	.	.	.	.	28	No	1.8
15-19	No	Yes	Yes	R501 - R2,500 pm	No	none	No	one	Yes	Yes	No	20	No	11	No	1.8
15-19	Yes	No	Yes	R2,501 - R6,000 pm	No	none	No	one	Yes	Yes	No	20	No	19	No	1.4

15-19	No	No	Yes	R501 - R2,500 pm	No	none	No	one	No	Yes	No	20	No	20	No	1.2
15-19	Yes	No	Yes	R2,501 - R6,000 pm	No	none	No	none	.	.	.	.	.	29	No	1.3
15-19	No	No	Yes	R501 - R2,500 pm	No	none	No	none	.	.	.	.	.	10	No	1.1
15-19	No	No	Yes	R501 - R2,500 pm	No	none	No	one	Yes	Yes	No	29	No	41	Yes	0.5
20-24	No	No	Yes	R501 - R2,500 pm	No	none	No	one	No	Yes	No	21	No	14	No	1.1
15-19	No	No	Yes	R501 - R2,500 pm	No	none	Yes	one	No	Yes	No	23	No	31	No	1.1
15-19	No	No	Yes	R501 - R2,500 pm	No	none	No	one	Yes	.	No	21	No	30	No	1.2
15-19	No	No	Yes	R501 - R2,500 pm	No	none	No	none	.	.	.	.	.	11	No	1.1
15-19	No	No	Yes	R2,501 - R6,000 pm	Yes	none	No	none	.	.	.	.	.	52	No	1.7
15-19	Yes	No	Yes	R501 - R2,500 pm	No	none	No	one	No	Yes	No	26	No	22	No	1.7
15-19	Yes	No	Yes	R501 - R2,500 pm	No	none	No	none	.	.	.	.	.	43	No	1.7
20-24	Yes	No	Yes	R2,501 - R6,000 pm	No	none	No	one	No	Yes	No	26	No	16	No	1.4
15-19	No	No	Yes	R2,501 - R6,000 pm	No	none	No	none	.	.	.	.	.	14	No	1.1
15-19	No	No	Yes	R501 - R2,500 pm	No	none	No	one	Yes	Yes	No	20	No	29	No	1.3
15-19	No	No	Yes	R2,501 - R6,000 pm	No	none	No	none	.	.	.	.	.	15	No	1.1
20-24	Yes	No	Yes	R501 - R2,500 pm	No	none	No	none	.	.	.	.	.	25	No	1.2
15-19	No	No	Yes	R2,501 - R6,000 pm	No	none	No	none	.	.	.	.	.	22	No	1.3
15-19	No	No	Yes	R501 - R2,500 pm	No	none	No	none	.	.	.	.	.	22	No	1.3
15-19	No	No	Yes	R0 - R500 pm	No	none	No	none	.	.	.	.	.	21	No	1.2
15-19	No	Yes	Yes	R501 - R2,500 pm	No	none	No	none	.	.	.	.	.	15	No	1.2
15-19	Yes	Yes	Yes	R501 - R2,500 pm	No	none	No	none	.	.	.	.	.	16	No	1
20-24	Yes	Yes	Yes	R501 - R2,500 pm	No	none	No	none	.	.	.	.	.	25	No	1.1
15-19	No	Yes	Yes	R0 - R500 pm	No	none	No	none	.	.	.	.	.	16	No	1
15-19	No	Yes	Yes	greater than R6,000 pm	No	none	No	none	.	.	.	.	.	23	No	1
20-24	Yes	Yes	Yes	R501 - R2,500 pm	No	none	No	none	.	.	.	.	.	18	No	1.2
15-19	No	Yes	Yes	greater than R6,000 pm	No	none	No	none	.	.	.	.	.	15	No	1.2
15-19	No	No	Yes	R2,501 - R6,000 pm	No	none	No	none	.	.	.	.	.	21	No	1.2
15-19	No	No	Yes	R2,501 - R6,000 pm	No	none	No	none	.	.	.	.	.	39	No	1.2
15-19	No	No	Yes	R2,501 - R6,000 pm	No	none	No	one	Yes	.	No	18	No	52	No	1.6
15-19	Yes	No	Yes	greater than R6,000 pm	No	none	No	one	No	No	No	21	No	15	No	1.9
15-19	Yes	No	Yes	R2,501 - R6,000 pm	No	none	No	one	Yes	No	Yes	26	No	29	Yes	0.8
15-19	No	No	Yes	R0 - R500 pm	No	none	No	none	.	.	.	.	.	30	No	1.7
15-19	No	No	Yes	R2,501 - R6,000 pm	Yes	none	No	none	.	.	.	.	.	56	No	1.3
15-19	No	No	Yes	R0 - R500 pm	No	none	No	none	.	.	.	.	.	47	No	1.3
15-19	No	No	Yes	R501 - R2,500 pm	No	none	No	one	No	Yes	No	21	No	30	No	1.3
20-24	No	No	Yes	R501 - R2,500 pm	No	none	No	none	.	.	.	.	.	44	No	1.8
15-19	No	No	Yes	R2,501 - R6,000 pm	No	none	No	none	.	.	.	.	.	53	No	1.6
15-19	No	No	Yes	R2,501 - R6,000 pm	No	none	No	none	.	.	.	.	.	52	No	1.6
20-24	No	No	Yes	R501 - R2,500 pm	No	none	No	none	.	.	.	.	.	60	No	1.4
15-19	No	No	Yes	R501 - R2,500 pm	No	none	No	none	.	.	.	.	.	53	No	1.4
15-19	No	No	Yes	R501 - R2,500 pm	No	none	No	none	.	.	.	.	.	24	No	1.4
15-19	Yes	No	Yes	R2,501 - R6,000 pm	No	none	No	none	.	.	.	.	.	14	No	1.5
20-24	Yes	No	No	R2,501 - R6,000 pm	No	none	No	none	.	.	.	.	.	14	No	1.4

15-19	No	No	Yes	greater than R6,000 pm	No	none	No	none	.	.	.	.	.	41	No	1.4
15-19	No	No	Yes	R2,501 - R6,000 pm	No	none	No	one	No	Yes	No	27	Yes	26	No	1.6
15-19	No	No	Yes	R2,501 - R6,000 pm	No	none	No	none	.	.	.	.	.	53	No	1.6
15-19	Yes	No	Yes	R501 - R2,500 pm	No	none	No	none	.	.	.	.	.	45	No	1.5
15-19	Yes	No	Yes	R2,501 - R6,000 pm	No	none	No	one	Yes	No	No	22	No	22	No	1.2
15-19	Yes	No	Yes	R501 - R2,500 pm	No	none	No	none	.	.	.	.	.	34	No	1.2
15-19	No	No	Yes	R501 - R2,500 pm	No	none	No	none	.	.	.	.	.	26	No	1.2
15-19	No	No	Yes	R501 - R2,500 pm	No	none	No	none	.	.	.	.	.	53	No	1.2
15-19	No	No	Yes	R501 - R2,500 pm	No	none	No	none	.	.	.	.	.	26	No	1.3
15-19	No	Yes	Yes	R501 - R2,500 pm	No	none	No	none	.	.	.	.	.	26	No	1
15-19	Yes	Yes	Yes	R2,501 - R6,000 pm	No	none	No	none	.	.	.	.	.	17	No	1
15-19	No	Yes	Yes	R2,501 - R6,000 pm	No	none	No	none	.	.	.	.	.	33	No	1.1
15-19	No	Yes	Yes	greater than R6,000 pm	No	none	No	2 or more	.	.	No	18	No	25	No	1.1
15-19	No	Yes	Yes	R2,501 - R6,000 pm	No	none	No	none	.	.	.	.	.	50	No	1.1
15-19	No	Yes	Yes	R501 - R2,500 pm	No	none	No	none	.	.	.	.	.	35	No	1.1
15-19	No	Yes	Yes	R2,501 - R6,000 pm	No	none	No	2 or more	No	Yes	No	25	Yes	33	No	1.8
15-19	No	Yes	Yes	R501 - R2,500 pm	No	none	No	none	.	.	.	.	.	8	No	1.7
15-19	No	Yes	Yes	R501 - R2,500 pm	No	none	No	none	.	.	.	.	.	17	No	1.7
15-19	Yes	Yes	Yes	R2,501 - R6,000 pm	No	none	No	none	.	.	.	.	.	25	No	1.3
15-19	No	Yes	Yes	R501 - R2,500 pm	No	none	No	one	No	.	No	22	No	9	Yes	0.6
15-19	No	Yes	Yes	R501 - R2,500 pm	No	none	No	none	.	.	.	.	.	9	No	1.3
15-19	No	Yes	Yes	R2,501 - R6,000 pm	No	none	No	one	No	Yes	No	22	No	25	No	1.3
15-19	Yes	Yes	Yes	R501 - R2,500 pm	No	none	No	one	Yes	Yes	No	24	No	18	No	1.3
15-19	No	Yes	Yes	R2,501 - R6,000 pm	No	none	No	one	No	.	No	22	No	19	No	1.4
15-19	No	Yes	No	greater than R6,000 pm	No	none	No	none	.	.	.	.	.	27	No	1.3
15-19	No	Yes	No	R501 - R2,500 pm	No	none	No	one	Yes	.	No	25	No	25	No	1.1
15-19	No	Yes	Yes	R501 - R2,500 pm	No	none	No	none	.	.	.	.	.	26	No	1.1
15-19	No	Yes	No	R501 - R2,500 pm	No	none	No	none	.	.	.	.	.	26	No	1.2
20-24	No	Yes	Yes	R2,501 - R6,000 pm	No	none	No	none	.	.	.	.	.	33	No	1.4
15-19	Yes	Yes	No	greater than R6,000 pm	No	none	No	none	.	.	.	.	.	9	No	1.4
20-24	No	Yes	Yes	R501 - R2,500 pm	Yes	none	No	none	.	.	.	.	.	11	No	1.6
15-19	No	Yes	Yes	R501 - R2,500 pm	No	none	No	one	No	Yes	No	18	No	29	No	1.6
15-19	No	Yes	Yes	R2,501 - R6,000 pm	No	none	No	none	.	.	.	.	.	52	No	1.4
15-19	No	Yes	Yes	greater than R6,000 pm	No	none	No	none	.	.	.	.	.	28	No	1.5
15-19	No	Yes	Yes	R501 - R2,500 pm	No	none	No	none	.	.	.	.	.	27	No	1.2
15-19	No	Yes	Yes	greater than R6,000 pm	No	none	No	none	.	.	.	.	.	32	No	1.6
15-19	No	Yes	Yes	R2,501 - R6,000 pm	No	none	No	none	.	.	.	.	.	17	No	1.5
15-19	Yes	Yes	Yes	greater than R6,000 pm	Yes	none	No	one	No	Yes	No	18	No	9	No	1.2
20-24	Yes	No	Yes	R501 - R2,500 pm	No	none	No	none	.	.	.	.	.	31	No	1.4

15-19	No	No	Yes	R501 - R2,500 pm	No	none	No	one	No	Yes	No	18	No	28	No	1.4
15-19	No	No	Yes	greater than R6,000 pm	No	none	No	one	Yes	Yes	No	21	No	35	No	1.8
15-19	No	No	No	R2,501 - R6,000 pm	No	none	No	one	No	.	No	31	No	53	Yes	0.9
15-19	No	No	Yes	R2,501 - R6,000 pm	No	none	No	none	.	.	.	.	.	19	No	1.4
15-19	No	Yes	Yes	R501 - R2,500 pm	No	none	No	none	.	.	.	.	.	23	No	1.8
15-19	No	Yes	Yes	R501 - R2,500 pm	No	none	No	none	.	.	.	.	.	34	No	1.8
15-19	No	Yes	Yes	greater than R6,000 pm	No	none	No	none	.	.	.	.	.	43	No	1.8
15-19	No	Yes	Yes	R501 - R2,500 pm	No	none	No	none	.	.	.	.	.	11	No	1.8
15-19	No	Yes	Yes	R501 - R2,500 pm	No	none	No	none	.	.	.	.	.	19	No	1.2
15-19	No	Yes	Yes	R501 - R2,500 pm	No	none	No	one	No	Yes	No	22	No	20	No	1.2
15-19	No	Yes	Yes	R2,501 - R6,000 pm	No	none	No	none	.	.	.	.	.	52	No	1.3
20-24	Yes	Yes	Yes	greater than R6,000 pm	No	none	No	none	.	.	.	.	.	22	No	1.7
15-19	No	Yes	Yes	R2,501 - R6,000 pm	No	none	No	none	.	.	.	.	.	16	No	1.1
15-19	No	Yes	Yes	R501 - R2,500 pm	Yes	none	No	none	.	.	.	.	.	15	No	1.1
20-24	Yes	Yes	Yes	R0 - R500 pm	No	none	No	none	.	.	.	.	.	19	No	1.4
15-19	No	Yes	Yes	R2,501 - R6,000 pm	No	none	No	none	.	.	.	.	.	27	No	1.3
15-19	No	Yes	Yes	R2,501 - R6,000 pm	No	none	No	none	.	.	.	.	.	53	No	1.3
15-19	No	Yes	Yes	R501 - R2,500 pm	No	none	No	none	.	.	.	.	.	36	No	1.3
15-19	No	Yes	Yes	greater than R6,000 pm	No	none	No	none	.	.	.	.	.	19	No	1.2
15-19	No	Yes	Yes	R2,501 - R6,000 pm	No	none	No	none	.	.	.	.	.	35	No	1.4
15-19	Yes	Yes	Yes	R501 - R2,500 pm	No	none	No	none	.	.	.	.	.	9	No	1.1
15-19	Yes	Yes	Yes	R2,501 - R6,000 pm	No	none	No	none	.	.	.	.	.	25	No	1.2
15-19	No	Yes	Yes	R501 - R2,500 pm	No	none	No	one	No	Yes	No	18	No	16	No	1.2
15-19	No	Yes	Yes	R2,501 - R6,000 pm	No	none	No	one	No	Yes	No	25	No	15	No	1.3
20-24	Yes	Yes	Yes	R2,501 - R6,000 pm	No	none	No	one	No	Yes	No	25	No	10	No	1.3
15-19	No	Yes	Yes	R501 - R2,500 pm	No	none	No	one	No	Yes	No	18	No	40	No	1.4
15-19	No	Yes	Yes	R2,501 - R6,000 pm	No	none	No	none	.	.	.	.	.	53	No	1.4
15-19	No	Yes	No	R501 - R2,500 pm	No	none	No	none	.	.	.	.	.	14	No	1.4
20-24	Yes	Yes	Yes	R2,501 - R6,000 pm	No	none	No	none	.	.	.	.	.	45	No	1.4
20-24	Yes	Yes	Yes	R2,501 - R6,000 pm	No	none	No	one	No	No	No	30	No	16	No	1.4
15-19	No	Yes	Yes	R2,501 - R6,000 pm	No	none	No	none	.	.	.	.	.	40	No	1.5
15-19	No	Yes	Yes	R501 - R2,500 pm	No	none	No	none	.	.	.	.	.	21	No	1.4
20-24	Yes	Yes	Yes	R501 - R2,500 pm	No	none	No	none	.	.	.	.	.	12	No	1.2
15-19	No	Yes	Yes	R2,501 - R6,000 pm	No	none	No	one	No	Yes	No	17	No	31	No	1.2
15-19	No	Yes	No	R501 - R2,500 pm	No	none	No	none	.	.	.	.	.	12	No	1.2
20-24	No	Yes	Yes	R501 - R2,500 pm	Yes	none	No	none	.	.	.	.	.	46	No	1.2
15-19	Yes	Yes	Yes	greater than R6,000 pm	No	none	No	one	Yes	Yes	No	19	No	56	No	1.1
15-19	No	Yes	Yes	R2,501 - R6,000 pm	No	none	No	none	.	.	.	.	.	10	No	1.7
15-19	No	Yes	Yes	R501 - R2,500 pm	No	none	No	one	No	Yes	No	21	No	19	No	1.7
15-19	No	Yes	Yes	R2,501 - R6,000 pm	No	none	No	none	.	.	.	.	.	27	No	1.4
15-19	No	Yes	Yes	R2,501 - R6,000 pm	No	none	No	none	.	.	.	.	.	10	No	1.4

15-19	Yes	Yes	Yes	R501 - R2,500 pm	No	none	No	one	Yes	Yes	No	23	No	10	Yes	0.7
15-19	Yes	Yes	Yes	R2,501 - R6,000 pm	No	none	No	none	.	.	.	.	.	26	No	1.7
15-19	Yes	Yes	Yes	greater than R6,000 pm	No	none	No	none	.	.	.	.	.	18	No	1.7
15-19	No	Yes	Yes	R501 - R2,500 pm	No	none	No	none	.	.	.	.	.	9	No	1.4
20-24	Yes	Yes	Yes	greater than R6,000 pm	No	none	No	none	.	.	.	.	.	10	No	1.4
15-19	No	Yes	Yes	R501 - R2,500 pm	No	none	No	none	.	.	.	.	.	9	No	1.5
15-19	Yes	Yes	Yes	R2,501 - R6,000 pm	No	none	No	none	.	.	.	.	.	38	No	1.5
15-19	No	Yes	No	R501 - R2,500 pm	No	none	No	none	.	.	.	.	.	9	No	1.5
15-19	No	Yes	No	greater than R6,000 pm	Yes	none	No	one	Yes	Yes	No	18	No	30	No	1.7
15-19	No	Yes	No	R501 - R2,500 pm	No	none	No	none	.	.	.	.	.	20	No	1.4
15-19	Yes	Yes	Yes	R501 - R2,500 pm	No	none	No	one	No	Yes	No	26	No	10	No	1.5
15-19	No	Yes	Yes	greater than R6,000 pm	No	none	No	none	.	.	.	.	.	52	No	1.3
15-19	No	Yes	Yes	R2,501 - R6,000 pm	No	none	No	one	No	.	No	18	No	42	No	1.4
15-19	No	Yes	Yes	R2,501 - R6,000 pm	No	none	No	none	.	.	.	.	.	11	No	1.5
15-19	No	Yes	Yes	R501 - R2,500 pm	No	none	No	none	.	.	.	.	.	43	No	1.5
15-19	No	Yes	Yes	R2,501 - R6,000 pm	No	none	No	none	.	.	.	.	.	32	No	1.9
20-24	Yes	Yes	Yes	R2,501 - R6,000 pm	No	none	Yes	none	.	.	.	.	.	38	No	1.5
20-24	Yes	No	Yes	R501 - R2,500 pm	No	none	No	none	.	.	.	.	.	32	No	2
15-19	No	No	Yes	R2,501 - R6,000 pm	No	none	No	one	No	No	No	19	No	27	No	1.7
15-19	No	No	Yes	greater than R6,000 pm	No	none	No	none	.	.	.	.	.	27	No	1.7
15-19	No	No	Yes	R501 - R2,500 pm	No	none	No	one	No	No	No	16	No	19	No	1.7
15-19	No	No	Yes	R501 - R2,500 pm	No	none	No	one	No	Yes	No	23	No	45	No	1.6
15-19	No	Yes	No	R501 - R2,500 pm	No	none	No	none	.	.	.	.	.	22	No	1.8
15-19	Yes	Yes	Yes	R2,501 - R6,000 pm	No	none	No	one	No	No	No	24	No	16	No	1.1
15-19	No	Yes	Yes	R501 - R2,500 pm	No	none	No	none	.	.	.	.	.	24	No	1.1
20-24	Yes	Yes	Yes	R2,501 - R6,000 pm	No	none	No	none	.	.	.	.	.	18	No	1
20-24	No	Yes	Yes	R501 - R2,500 pm	No	none	No	none	.	.	.	.	.	17	No	1.1
15-19	No	Yes	Yes	R2,501 - R6,000 pm	No	none	No	none	.	.	.	.	.	16	No	1.1
15-19	No	Yes	Yes	greater than R6,000 pm	No	none	No	none	.	.	.	.	.	33	No	1.3
20-24	No	Yes	Yes	R0 - R500 pm	No	none	No	one	No	No	No	27	No	20	No	1.4
15-19	No	Yes	Yes	R2,501 - R6,000 pm	No	none	No	one	No	Yes	No	19	No	15	No	1.3
20-24	No	Yes	No	R501 - R2,500 pm	No	none	No	none	.	.	.	.	.	35	No	1.4
15-19	Yes	Yes	Yes	R501 - R2,500 pm	No	none	No	2 or more	No	Yes	No	23	Yes	41	No	1.3
15-19	No	Yes	Yes	R2,501 - R6,000 pm	No	none	No	none	.	.	.	.	.	11	No	1.3
20-24	Yes	Yes	Yes	greater than R6,000 pm	No	none	No	none	.	.	.	.	.	12	No	1.3
20-24	Yes	Yes	Yes	R2,501 - R6,000 pm	No	none	No	none	.	.	.	.	.	44	No	1.2
15-19	Yes	Yes	Yes	R2,501 - R6,000 pm	No	none	No	none	.	.	.	.	.	37	No	1.3
15-19	No	Yes	Yes	R2,501 - R6,000 pm	No	none	No	none	.	.	.	.	.	53	No	1
15-19	No	Yes	Yes	R501 - R2,500 pm	No	none	No	2 or more	Yes	Yes	No	17	No	39	No	1.4

15-19	No	Yes	Yes	greater than R6,000 pm	No	none	No	none	.	.	.	.	.	52	No	1.4
15-19	No	Yes	Yes	R2,501 - R6,000 pm	No	none	No	none	.	.	.	.	.	9	No	1.1
15-19	Yes	Yes	Yes	R0 - R500 pm	Yes	none	No	one	Yes	Yes	No	21	No	30	No	1.1
15-19	No	Yes	Yes	R501 - R2,500 pm	No	none	No	none	.	.	.	.	.	25	No	1.1
15-19	Yes	Yes	Yes	R2,501 - R6,000 pm	No	none	.	none	.	.	.	.	.	24	No	1.1
15-19	No	Yes	Yes	R501 - R2,500 pm	No	none	No	none	.	.	.	.	.	25	No	1.1
15-19	No	Yes	Yes	greater than R6,000 pm	No	none	No	none	.	.	.	.	.	24	No	1.1
20-24	Yes	Yes	Yes	R501 - R2,500 pm	No	none	No	one	No	Yes	No	23	No	42	No	1.3
20-24	No	Yes	Yes	R2,501 - R6,000 pm	Yes	none	No	none	.	.	.	.	.	17	No	1
15-19	No	Yes	Yes	R501 - R2,500 pm	No	none	No	none	.	.	.	.	.	50	No	1.1
15-19	No	Yes	Yes	R501 - R2,500 pm	No	none	No	none	.	.	.	.	.	19	No	1.4
15-19	No	No	Yes	R2,501 - R6,000 pm	No	none	No	none	.	.	.	.	.	9	No	1.1
15-19	No	No	Yes	R2,501 - R6,000 pm	No	none	No	none	.	.	.	.	.	9	No	1.1
15-19	No	No	Yes	R2,501 - R6,000 pm	No	none	No	none	.	.	.	.	.	9	No	1.1
15-19	No	No	Yes	R2,501 - R6,000 pm	No	none	No	none	.	.	.	.	.	18	No	1.1
15-19	No	No	Yes	R2,501 - R6,000 pm	No	none	No	none	.	.	.	.	.	28	No	1.1
15-19	Yes	No	Yes	R501 - R2,500 pm	No	none	No	one	No	Yes	No	25	No	52	No	1.6
15-19	No	No	Yes	R501 - R2,500 pm	No	none	No	none	.	.	.	.	.	42	No	2
15-19	No	No	Yes	R2,501 - R6,000 pm	No	none	No	none	.	.	.	.	.	28	No	1.6
20-24	Yes	No	Yes	R2,501 - R6,000 pm	No	none	No	none	.	.	.	.	.	33	No	1.6
15-19	No	No	Yes	R501 - R2,500 pm	No	none	No	none	.	.	.	.	.	52	No	1.7
15-19	No	No	Yes	R2,501 - R6,000 pm	No	none	No	none	.	.	.	.	.	53	No	1.6
15-19	No	No	Yes	R501 - R2,500 pm	No	none	No	none	.	.	.	.	.	51	No	1.3
15-19	No	No	Yes	R2,501 - R6,000 pm	No	none	No	none	.	.	.	.	.	51	No	1.3
15-19	No	No	Yes	R2,501 - R6,000 pm	No	none	No	one	No	No	No	32	No	13	No	1.5
20-24	Yes	Yes	No	R501 - R2,500 pm	No	none	No	none	.	.	.	.	.	24	No	1.6
15-19	No	No	Yes	R2,501 - R6,000 pm	No	none	No	one	No	Yes	No	22	No	53	No	1.7
15-19	No	No	Yes	greater than R6,000 pm	No	none	No	none	.	.	.	.	.	53	No	1.8
15-19	No	No	Yes	R501 - R2,500 pm	No	none	No	none	.	.	.	.	.	53	No	1.7
15-19	No	No	Yes	R2,501 - R6,000 pm	Yes	none	No	one	Yes	Yes	No	19	No	52	No	1.7
15-19	No	No	Yes	greater than R6,000 pm	No	none	No	none	.	.	.	.	.	42	No	1.1
15-19	No	No	Yes	R501 - R2,500 pm	No	none	No	none	.	.	.	.	.	27	No	1.1
20-24	Yes	No	Yes	R2,501 - R6,000 pm	No	none	No	one	No	No	No	32	No	57	No	1.3
15-19	No	No	Yes	R501 - R2,500 pm	No	none	No	none	.	.	.	.	.	14	No	1.3
15-19	Yes	No	Yes	R501 - R2,500 pm	No	none	No	one	No	No	No	21	No	55	Yes	0.6
15-19	No	No	Yes	R2,501 - R6,000 pm	No	none	No	none	.	.	.	.	.	27	No	1.4
15-19	No	No	Yes	R2,501 - R6,000 pm	Yes	none	No	none	.	.	.	.	.	39	No	1.2
15-19	No	No	Yes	R501 - R2,500 pm	No	none	No	one	No	No	No	18	No	27	No	1.2
15-19	No	No	Yes	R501 - R2,500 pm	No	none	No	none	.	.	.	.	.	52	No	1.3
20-24	No	No	Yes	R501 - R2,500 pm	No	none	No	none	.	.	.	.	.	43	No	1.2
15-19	No	No	Yes	R2,501 - R6,000 pm	No	none	No	none	.	.	.	.	.	37	No	1.3
15-19	No	No	Yes	R2,501 - R6,000 pm	No	none	No	none	.	.	.	.	.	26	No	1.3

15-19	No	No	Yes	R2,501 - R6,000 pm	No	none	No	none	.	.	.	.	.	26	No	1.3
15-19	No	No	Yes	R501 - R2,500 pm	No	none	No	none	.	.	.	.	.	49	No	1.2
15-19	No	No	Yes	R2,501 - R6,000 pm	No	none	No	one	No	Yes	No	21	No	15	No	1.8
20-24	Yes	No	Yes	R2,501 - R6,000 pm	No	none	No	none	.	.	.	.	.	17	No	1.8
15-19	No	No	Yes	R2,501 - R6,000 pm	No	none	No	none	.	.	.	.	.	53	No	1.8
15-19	No	No	Yes	R2,501 - R6,000 pm	No	none	No	none	.	.	.	.	.	44	No	1.8
15-19	Yes	No	Yes	R501 - R2,500 pm	No	none	No	one	No	Yes	No	23	No	21	No	1.1
20-24	Yes	No	Yes	R501 - R2,500 pm	No	none	No	one	No	Yes	No	28	No	44	No	1.1
15-19	No	No	Yes	R2,501 - R6,000 pm	No	none	No	none	.	.	.	.	.	28	No	1.3
15-19	No	No	Yes	greater than R6,000 pm	No	none	No	none	.	.	.	.	.	27	No	1.4
15-19	No	No	Yes	R2,501 - R6,000 pm	No	none	No	none	.	.	.	.	.	18	No	1.4
20-24	No	No	Yes	R2,501 - R6,000 pm	No	none	No	none	.	.	.	.	.	30	No	1.3
15-19	No	No	Yes	R501 - R2,500 pm	No	none	No	none	.	.	.	.	.	9	No	1.1
20-24	Yes	No	Yes	R501 - R2,500 pm	No	none	No	one	No	No	No	25	Yes	25	No	1.6
15-19	No	No	Yes	R501 - R2,500 pm	No	none	No	none	.	.	.	.	.	38	No	1.2
20-24	No	No	Yes	greater than R6,000 pm	No	none	No	none	.	.	.	.	.	42	No	1.1
20-24	Yes	No	Yes	R501 - R2,500 pm	No	none	No	none	.	.	.	.	.	43	No	1.3
15-19	No	No	Yes	R2,501 - R6,000 pm	No	none	No	none	.	.	.	.	.	41	No	1.2
15-19	Yes	No	Yes	R501 - R2,500 pm	No	none	No	none	.	.	.	.	.	23	No	1.6
15-19	No	No	Yes	greater than R6,000 pm	No	none	No	none	.	.	.	.	.	42	No	1.6
15-19	No	No	Yes	R501 - R2,500 pm	No	none	No	none	.	.	.	.	.	32	No	1.7
15-19	No	No	Yes	R501 - R2,500 pm	No	none	No	one	Yes	Yes	No	26	No	21	No	1.6
15-19	No	No	Yes	R501 - R2,500 pm	No	none	No	one	No	.	No	19	No	11	No	1.6
15-19	No	Yes	No	R2,501 - R6,000 pm	Yes	one	Yes	one	Yes	Yes	No	23	No	12	No	1.2
20-24	Yes	Yes	Yes	R501 - R2,500 pm	No	one	No	one	Yes	Yes	No	29	No	11	No	1.6
15-19	No	Yes	Yes	R501 - R2,500 pm	No	one	No	one	Yes	Yes	No	23	No	12	No	2
15-19	Yes	No	Yes	R0 - R500 pm	Yes	one	No	one	No	No	No	26	No	39	No	1.5
15-19	No	No	Yes	R2,501 - R6,000 pm	Yes	one	Yes	one	No	No	No	22	No	28	No	1.3
15-19	Yes	Yes	Yes	R0 - R500 pm	Yes	one	Yes	one	No	No	No	28	No	12	No	2
20-24	Yes	Yes	Yes	R501 - R2,500 pm	Yes	one	Yes	one	No	Yes	No	25	No	28	Yes	1
15-19	No	No	Yes	R501 - R2,500 pm	No	one	Yes	one	No	No	No	23	No	85	No	1.3
20-24	Yes	Yes	Yes	R501 - R2,500 pm	Yes	one	No	one	No	Yes	No	26	No	9	No	2.1
20-24	Yes	Yes	Yes	.	Yes	one	Yes	one	No	No	No	27	Yes	9	No	2.2
15-19	No	Yes	Yes	R501 - R2,500 pm	Yes	one	Yes	one	No	No	No	24	No	14	No	2
15-19	Yes	No	Yes	R2,501 - R6,000 pm	Yes	one	No	one	Yes	No	No	27	No	41	No	1.6
20-24	Yes	Yes	Yes	R501 - R2,500 pm	No	one	Yes	none	.	.	No	22	.	9	No	1.9
20-24	Yes	Yes	Yes	.	Yes	one	Yes	one	No	No	No	29	No	20	No	1.7
15-19	Yes	No	Yes	R501 - R2,500 pm	No	one	No	one	No	No	No	29	No	33	No	2
20-24	Yes	No	Yes	R501 - R2,500 pm	Yes	one	No	one	No	No	No	25	Yes	53	No	1.7
20-24	No	Yes	No	.	Yes	one	Yes	one	No	Yes	No	28	No	11	No	2.6
20-24	Yes	Yes	Yes	greater than R6,000 pm	Yes	one	Yes	one	Yes	No	No	25	No	6	No	1.7
20-24	Yes	No	Yes	R501 - R2,500 pm	Yes	one	.	one	Yes	Yes	No	25	No	91	No	1.2

20-24	Yes	No	Yes	R501 - R2,500 pm	Yes	one	Yes	one	No	No	No	24	No	30	No	2
15-19	Yes	Yes	Yes	R501 - R2,500 pm	Yes	one	Yes	one	No	No	No	23	No	13	No	1.9
20-24	No	No	Yes	R0 - R500 pm	Yes	one	Yes	one	Yes	Yes	Yes	26	No	47	No	1.3
20-24	Yes	Yes	Yes	.	No	one	Yes	one	No	Yes	No	26	No	13	No	2.2
15-19	No	Yes	Yes	R501 - R2,500 pm	No	one	Yes	one	No	No	No	19	No	41	No	1.2
20-24	Yes	Yes	Yes	R2,501 - R6,000 pm	Yes	one	No	one	Yes	No	No	28	No	11	No	1.3
20-24	No	Yes	Yes	R501 - R2,500 pm	Yes	one	Yes	one	No	No	No	25	No	32	No	1.2
20-24	Yes	Yes	Yes	R501 - R2,500 pm	Yes	one	Yes	one	No	Yes	No	25	No	18	No	2.1
20-24	Yes	No	Yes	R2,501 - R6,000 pm	Yes	one	No	one	Yes	No	No	24	No	46	No	1.3
15-19	Yes	Yes	Yes	R501 - R2,500 pm	Yes	one	No	one	No	Yes	No	22	No	21	No	1.8
20-24	No	No	Yes	R501 - R2,500 pm	Yes	one	Yes	one	No	Yes	No	26	No	87	No	1.4
15-19	Yes	No	Yes	R501 - R2,500 pm	No	one	Yes	2 or more	No	Yes	No	22	Yes	67	No	1.5
20-24	Yes	Yes	Yes	R2,501 - R6,000 pm	Yes	one	Yes	one	No	Yes	No	28	No	17	No	1.2
20-24	Yes	Yes	Yes	R501 - R2,500 pm	Yes	one	Yes	one	No	Yes	No	33	No	12	No	2.2
20-24	No	No	Yes	R0 - R500 pm	Yes	one	Yes	one	No	No	No	30	No	20	No	2
20-24	Yes	No	Yes	R2,501 - R6,000 pm	No	one	Yes	one	Yes	Yes	No	24	No	87	No	1.3
20-24	No	Yes	Yes	R501 - R2,500 pm	No	one	No	none	.	.	No	25	.	16	No	1.2
15-19	No	Yes	Yes	R501 - R2,500 pm	Yes	one	No	none	.	No	No	18	.	20	No	1.3
15-19	No	Yes	No	.	Yes	one	Yes	one	No	Yes	No	20	No	15	No	1.2
20-24	Yes	Yes	Yes	.	Yes	one	Yes	one	No	No	Yes	27	No	18	No	2.1
15-19	Yes	Yes	Yes	R501 - R2,500 pm	Yes	one	No	none	.	Yes	No	22	.	11	No	2.3
15-19	No	No	Yes	R501 - R2,500 pm	Yes	one	Yes	none	.	No	No	21	.	26	No	1
20-24	No	Yes	Yes	R2,501 - R6,000 pm	Yes	one	Yes	none	.	.	No	24	.	14	No	1.6
20-24	Yes	Yes	Yes	R2,501 - R6,000 pm	Yes	one	No	one	No	Yes	No	27	No	18	No	2.3
15-19	No	No	Yes	R501 - R2,500 pm	No	one	No	one	No	Yes	No	24	No	42	No	1.7
20-24	Yes	Yes	Yes	R501 - R2,500 pm	Yes	one	No	one	No	No	No	28	Yes	18	No	1.7
15-19	No	No	Yes	R501 - R2,500 pm	No	one	No	none	.	.	.	.	.	81	No	1.5
20-24	No	Yes	No	R0 - R500 pm	Yes	one	Yes	2 or more	No	Yes	No	30	No	6	No	1.8
20-24	Yes	Yes	Yes	greater than R6,000 pm	Yes	one	Yes	one	No	No	No	25	No	26	No	1.2
20-24	No	Yes	Yes	R501 - R2,500 pm	Yes	one	Yes	none	.	Yes	No	18	.	13	No	1.6
20-24	No	Yes	Yes	R501 - R2,500 pm	Yes	one	No	one	No	No	No	31	No	10	No	1.7
20-24	Yes	Yes	No	R501 - R2,500 pm	Yes	one	No	one	No	No	No	27	No	9	No	1.9
15-19	Yes	Yes	Yes	R0 - R500 pm	Yes	one	Yes	one	No	No	No	30	No	10	No	1.6
20-24	Yes	Yes	No	R0 - R500 pm	No	one	Yes	one	Yes	No	No	27	No	27	No	1.4
20-24	Yes	No	Yes	R2,501 - R6,000 pm	Yes	one	Yes	one	No	No	No	32	No	44	No	1.9
20-24	No	Yes	Yes	.	Yes	one	No	one	No	No	No	22	No	19	No	1.9
15-19	No	No	Yes	R501 - R2,500 pm	No	one	Yes	one	No	Yes	No	26	No	22	No	1.3
20-24	Yes	No	Yes	R2,501 - R6,000 pm	No	one	Yes	one	No	Yes	No	30	Yes	12	No	1.5
15-19	Yes	No	No	R501 - R2,500 pm	Yes	one	No	none	.	Yes	No	20	.	62	Yes	0.6
20-24	Yes	No	No	R501 - R2,500 pm	Yes	one	Yes	one	No	No	No	27	No	30	No	2
15-19	Yes	No	Yes	R0 - R500 pm	Yes	one	Yes	one	No	Yes	No	23	No	52	No	1.8
20-24	No	No	Yes	R0 - R500 pm	No	one	No	one	No	.	No	25	No	15	No	1.4
20-24	No	No	No	R0 - R500 pm	Yes	one	Yes	one	No	No	No	28	No	91	No	1.5
15-19	No	Yes	Yes	R501 - R2,500 pm	Yes	one	No	one	No	Yes	No	19	No	10	No	2.1
15-19	Yes	Yes	No	R2,501 - R6,000 pm	Yes	one	No	one	No	No	No	24	No	36	No	1.5

15-19	No	Yes	Yes	R501 - R2,500 pm	Yes	one	No	2 or more	No	Yes	No	21	No	36	Yes	0.9
20-24	Yes	No	Yes	R501 - R2,500 pm	Yes	one	No	one	No	No	No	23	No	40	No	1.4
20-24	Yes	No	Yes	R0 - R500 pm	Yes	one	Yes	one	Yes	No	No	25	No	47	No	1.5
20-24	No	No	Yes	R501 - R2,500 pm	Yes	one	Yes	none	.	No	No	25	.	24	No	1.7
15-19	No	Yes	Yes	R501 - R2,500 pm	Yes	one	No	one	Yes	Yes	No	22	Yes	23	No	2
20-24	Yes	Yes	Yes	R0 - R500 pm	No	one	Yes	one	No	No	No	27	Yes	17	No	1.9
20-24	Yes	Yes	Yes	R2,501 - R6,000 pm	No	one	Yes	2 or more	No	Yes	No	25	No	33	Yes	0.7
15-19	No	Yes	Yes	R0 - R500 pm	No	one	Yes	one	No	No	No	29	No	12	No	1.6
20-24	Yes	Yes	Yes	R2,501 - R6,000 pm	Yes	one	Yes	one	Yes	Yes	No	24	No	27	No	1.3
15-19	Yes	Yes	Yes	R501 - R2,500 pm	No	one	No	one	No	Yes	No	26	No	14	No	1.3
15-19	Yes	Yes	Yes	R501 - R2,500 pm	Yes	one	Yes	one	No	Yes	No	21	No	8	No	1.8
15-19	No	No	Yes	R501 - R2,500 pm	Yes	one	Yes	none	.	Yes	No	18	.	86	No	2.2
15-19	No	Yes	Yes	R501 - R2,500 pm	No	one	No	one	Yes	Yes	No	18	No	23	No	2.2
20-24	No	Yes	Yes	R501 - R2,500 pm	No	one	Yes	one	No	No	No	31	No	24	No	1.4
15-19	Yes	Yes	Yes	R501 - R2,500 pm	No	one	Yes	one	No	No	No	27	No	17	No	2.3
20-24	No	Yes	Yes	R0 - R500 pm	Yes	one	Yes	one	No	No	No	27	Yes	30	No	1.8
20-24	Yes	No	Yes	R501 - R2,500 pm	Yes	one	Yes	one	No	No	No	30	No	23	Yes	0.8
15-19	No	No	Yes	R0 - R500 pm	No	one	No	none	.	No	No	21	.	17	Yes	0.7
20-24	No	No	Yes	R2,501 - R6,000 pm	Yes	one	Yes	none	.	.	.	.	.	47	No	1.2
20-24	Yes	No	Yes	R501 - R2,500 pm	Yes	one	No	none	.	No	No	22	.	43	No	1.9
15-19	Yes	No	Yes	R2,501 - R6,000 pm	No	one	No	one	No	Yes	No	27	No	82	No	1.4
20-24	Yes	Yes	Yes	R501 - R2,500 pm	Yes	one	Yes	2 or more	No	Yes	No	26	No	35	No	1.9
15-19	Yes	Yes	No	R501 - R2,500 pm	Yes	one	Yes	none	.	No	Yes	21	.	27	No	1.8
20-24	Yes	Yes	Yes	greater than R6,000 pm	Yes	one	Yes	one	Yes	Yes	No	26	No	21	No	1.8
15-19	No	Yes	No	.	Yes	one	Yes	one	No	No	No	23	No	13	No	1.7
20-24	No	Yes	Yes	R501 - R2,500 pm	Yes	one	Yes	one	No	No	No	26	No	33	No	2.1
20-24	Yes	Yes	Yes	R0 - R500 pm	Yes	one	No	one	No	No	No	26	No	11	No	1.6
20-24	Yes	Yes	Yes	greater than R6,000 pm	No	one	Yes	one	No	No	No	30	Yes	21	No	1.9
20-24	Yes	Yes	Yes	R2,501 - R6,000 pm	Yes	one	Yes	one	No	No	No	26	No	11	No	1.3
20-24	Yes	Yes	Yes	R501 - R2,500 pm	No	one	No	2 or more	No	Yes	No	28	No	10	No	1.8
15-19	No	No	Yes	R501 - R2,500 pm	No	one	Yes	one	No	Yes	No	22	No	65	No	1.3
20-24	Yes	No	Yes	R501 - R2,500 pm	Yes	one	.	2 or more	.	.	No	22	No	16	No	2.6
20-24	No	No	Yes	R501 - R2,500 pm	No	one	No	none	.	.	No	25	.	30	No	2
15-19	Yes	No	Yes	R501 - R2,500 pm	No	one	Yes	one	No	Yes	No	20	No	7	No	1.2
20-24	No	Yes	No	R501 - R2,500 pm	Yes	one	Yes	one	Yes	Yes	No	29	No	12	No	1.9
15-19	No	Yes	Yes	R501 - R2,500 pm	No	one	Yes	one	No	Yes	No	22	Yes	15	No	1.9
20-24	No	Yes	Yes	R501 - R2,500 pm	No	one	No	one	No	No	No	25	No	81	No	1.3
20-24	Yes	Yes	Yes	greater than R6,000 pm	Yes	one	No	one	No	No	No	27	No	8	No	2
20-24	No	No	Yes	R501 - R2,500 pm	Yes	one	No	one	No	No	No	24	No	45	No	1.3
20-24	Yes	Yes	Yes	R2,501 - R6,000 pm	No	one	No	none	.	.	.	.	.	16	No	2.3
20-24	Yes	Yes	Yes	R0 - R500 pm	Yes	one	No	one	No	Yes	No	25	No	12	No	1.9
15-19	Yes	No	Yes	R501 - R2,500 pm	Yes	one	Yes	one	No	Yes	No	23	Yes	25	No	2.1
20-24	Yes	No	Yes	R501 - R2,500 pm	Yes	one	Yes	one	No	No	No	27	No	26	No	1.2

15-19	Yes	No	Yes	R501 - R2,500 pm	Yes	one	Yes	one	No	No	No	22	No	30	No	1.5
20-24	No	No	Yes	R0 - R500 pm	Yes	one	No	one	No	Yes	No	28	No	51	No	1.3
15-19	No	Yes	Yes	R501 - R2,500 pm	Yes	one	Yes	one	Yes	Yes	No	24	No	20	No	1.3
20-24	Yes	Yes	Yes	R0 - R500 pm	Yes	one	No	one	No	Yes	No	36	No	28	No	1.7
20-24	No	Yes	Yes	R501 - R2,500 pm	Yes	one	Yes	2 or more	No	No	No	23	No	10	No	1.8
20-24	Yes	Yes	Yes	R501 - R2,500 pm	Yes	one	Yes	one	No	Yes	No	27	No	27	No	1.2
20-24	Yes	No	Yes	greater than R6,000 pm	Yes	one	Yes	none	.	Yes	No	31	.	35	No	1.3
15-19	No	No	Yes	R0 - R500 pm	No	one	No	one	No	Yes	No	19	No	37	No	1.4
15-19	No	No	Yes	R2,501 - R6,000 pm	Yes	one	No	one	Yes	No	No	25	No	32	No	2.3
20-24	No	No	Yes	R2,501 - R6,000 pm	Yes	one	No	none	.	Yes	No	28	.	46	No	1.3
15-19	No	Yes	Yes	R501 - R2,500 pm	No	one	Yes	one	No	No	No	22	No	10	No	1.7
15-19	No	Yes	No	R0 - R500 pm	No	one	Yes	one	No	Yes	No	18	No	12	No	1.7
15-19	No	Yes	No	R0 - R500 pm	Yes	one	Yes	one	No	Yes	No	22	No	37	No	1.9
20-24	Yes	No	Yes	R0 - R500 pm	Yes	one	Yes	none	.	Yes	No	26	.	57	No	1.7
20-24	Yes	No	Yes	.	Yes	one	No	none	.	.	.	.	.	30	No	1.8
15-19	Yes	Yes	No	R2,501 - R6,000 pm	Yes	one	No	none	.	Yes	No	21	.	25	No	1.7
20-24	Yes	No	No	R501 - R2,500 pm	Yes	one	Yes	one	No	Yes	No	34	No	26	No	1.4
15-19	No	No	Yes	R501 - R2,500 pm	Yes	one	Yes	one	No	Yes	No	21	No	21	No	1.7
15-19	Yes	No	No	R501 - R2,500 pm	No	one	Yes	one	No	Yes	No	26	No	22	No	1.1
20-24	Yes	No	No	R0 - R500 pm	No	one	Yes	none	.	No	No	33	.	30	No	1.8
20-24	No	Yes	Yes	R0 - R500 pm	No	one	Yes	none	.	Yes	No	28	.	12	No	2.3
20-24	Yes	No	Yes	R501 - R2,500 pm	Yes	one	Yes	one	Yes	Yes	No	23	Yes	30	No	1.8
20-24	Yes	Yes	Yes	R2,501 - R6,000 pm	Yes	one	Yes	one	Yes	Yes	No	24	No	12	No	1.4
20-24	Yes	Yes	Yes	R501 - R2,500 pm	Yes	one	Yes	one	No	No	Yes	32	No	34	No	2
20-24	Yes	Yes	No	R0 - R500 pm	No	one	No	one	No	Yes	No	31	No	10	No	2
15-19	Yes	Yes	Yes	R501 - R2,500 pm	No	one	No	2 or more	No	Yes	No	43	No	28	No	2
20-24	Yes	No	Yes	R501 - R2,500 pm	Yes	one	Yes	one	Yes	No	No	28	Yes	26	No	1.3
20-24	Yes	No	Yes	R2,501 - R6,000 pm	Yes	one	Yes	one	No	Yes	No	23	No	21	No	2
15-19	No	No	Yes	R501 - R2,500 pm	Yes	one	Yes	none	.	No	No	25	.	28	No	1.8
20-24	No	No	Yes	R501 - R2,500 pm	Yes	one	Yes	one	No	Yes	No	27	No	80	No	1.4
15-19	No	Yes	Yes	.	Yes	one	Yes	one	No	No	No	24	No	13	No	1.9
20-24	No	No	Yes	R2,501 - R6,000 pm	Yes	one	Yes	one	No	No	No	28	No	91	No	1.4
20-24	Yes	Yes	Yes	R2,501 - R6,000 pm	No	one	Yes	one	No	Yes	No	23	No	20	No	1.2
20-24	Yes	No	Yes	R501 - R2,500 pm	No	one	No	one	No	Yes	No	26	Yes	15	No	1.2
15-19	No	Yes	Yes	.	Yes	one	Yes	one	No	No	No	20	No	17	No	1.8
20-24	Yes	Yes	Yes	R0 - R500 pm	Yes	one	Yes	one	No	No	No	23	No	11	No	2.3
15-19	Yes	No	Yes	R501 - R2,500 pm	No	one	Yes	one	No	No	No	29	No	49	No	1.4
15-19	No	No	Yes	R2,501 - R6,000 pm	No	one	No	one	No	Yes	No	25	No	30	No	1.8
15-19	Yes	Yes	No	R501 - R2,500 pm	No	one	No	none	.	No	No	18	.	10	Yes	0.8
20-24	Yes	Yes	Yes	R2,501 - R6,000 pm	Yes	one	Yes	one	No	Yes	No	26	No	49	No	1.9
20-24	Yes	Yes	Yes	R2,501 - R6,000 pm	No	one	No	one	No	No	No	24	No	20	No	2.2
15-19	Yes	Yes	Yes	R2,501 - R6,000 pm	Yes	one	No	one	No	Yes	No	25	No	11	Yes	0.9
15-19	No	Yes	Yes	R501 - R2,500 pm	No	one	No	none	.	Yes	No	18	.	34	No	1.6
20-24	Yes	Yes	Yes	R0 - R500 pm	Yes	one	No	one	No	Yes	No	29	No	10	No	1.9
20-24	Yes	No	Yes	R2,501 - R6,000 pm	Yes	one	Yes	none	.	No	No	25	.	76	No	1.6

15-19	No	Yes	Yes	R501 - R2,500 pm	Yes	one	Yes	none	.	Yes	No	21	.	25	No	1.2
20-24	Yes	Yes	Yes	R2,501 - R6,000 pm	No	one	Yes	one	No	Yes	No	26	No	12	No	1.8
15-19	No	Yes	Yes	.	Yes	one	No	one	Yes	Yes	No	18	No	17	No	2
15-19	No	No	Yes	R501 - R2,500 pm	Yes	one	No	one	No	Yes	No	23	No	67	No	2
15-19	Yes	No	Yes	R2,501 - R6,000 pm	Yes	one	Yes	one	No	Yes	No	26	No	35	No	1.3
15-19	No	No	Yes	R501 - R2,500 pm	Yes	one	Yes	one	Yes	Yes	No	23	No	27	No	1.4
15-19	No	Yes	Yes	R0 - R500 pm	No	one	Yes	2 or more	No	Yes	No	24	No	14	No	1.9
15-19	No	No	Yes	R2,501 - R6,000 pm	Yes	one	Yes	2 or more	Yes	No	No	30	No	24	No	1.5
20-24	Yes	Yes	Yes	greater than R6,000 pm	Yes	one	No	one	No	Yes	No	28	No	13	No	2.2
20-24	Yes	Yes	No	R501 - R2,500 pm	Yes	one	Yes	one	No	No	No	27	No	10	No	1.8
20-24	Yes	Yes	Yes	R0 - R500 pm	Yes	one	Yes	one	No	Yes	No	38	No	11	No	1.9
15-19	No	No	Yes	R501 - R2,500 pm	Yes	one	No	one	No	No	No	36	No	7	No	1.3
15-19	No	No	Yes	R2,501 - R6,000 pm	Yes	one	No	one	No	Yes	No	20	No	46	No	1.2
15-19	Yes	Yes	Yes	R501 - R2,500 pm	Yes	one	Yes	one	No	Yes	No	23	No	10	No	1.6
15-19	Yes	No	Yes	R0 - R500 pm	Yes	one	No	one	No	Yes	No	20	No	67	No	1.5
20-24	Yes	No	Yes	R2,501 - R6,000 pm	Yes	one	Yes	one	Yes	Yes	No	26	No	87	No	1.5
20-24	Yes	Yes	Yes	R2,501 - R6,000 pm	Yes	one	No	one	Yes	Yes	No	21	No	41	No	1.7
20-24	No	Yes	Yes	.	Yes	one	Yes	2 or more	.	Yes	No	29	No	35	No	1.9
15-19	No	Yes	Yes	.	No	one	Yes	one	Yes	Yes	No	23	No	8	No	1
20-24	Yes	No	Yes	R501 - R2,500 pm	Yes	one	Yes	one	Yes	No	No	30	No	23	No	1.5
15-19	Yes	Yes	Yes	R2,501 - R6,000 pm	No	one	Yes	one	No	.	No	28	Yes	17	No	1.6
20-24	Yes	No	No	R501 - R2,500 pm	Yes	one	Yes	one	No	No	No	25	No	59	No	1.2
15-19	Yes	No	Yes	R2,501 - R6,000 pm	Yes	one	Yes	one	Yes	No	No	23	No	39	No	1.3
15-19	Yes	No	No	R2,501 - R6,000 pm	Yes	one	Yes	one	No	No	No	30	No	41	No	1.3
20-24	Yes	No	No	R501 - R2,500 pm	Yes	one	Yes	one	No	No	No	24	No	24	No	2.2
20-24	Yes	Yes	Yes	R2,501 - R6,000 pm	No	one	No	2 or more	No	No	No	23	Yes	9	No	2.2
15-19	No	Yes	Yes	R501 - R2,500 pm	Yes	one	No	one	No	Yes	No	20	No	15	No	1.4
15-19	Yes	Yes	Yes	R2,501 - R6,000 pm	Yes	one	No	one	No	No	No	24	No	10	No	1.9
20-24	Yes	Yes	Yes	R2,501 - R6,000 pm	No	one	Yes	one	No	No	No	31	No	23	No	1.6
20-24	No	Yes	Yes	.	Yes	one	Yes	2 or more	No	Yes	No	26	Yes	49	Yes	1.2
20-24	No	No	Yes	R0 - R500 pm	No	one	Yes	one	Yes	No	No	24	No	30	No	1.8
20-24	No	No	Yes	R501 - R2,500 pm	Yes	one	Yes	none	.	.	.	.	.	68	No	1.5
20-24	Yes	Yes	Yes	R2,501 - R6,000 pm	No	one	Yes	none	.	No	No	21	.	10	No	1.6
15-19	No	No	Yes	R501 - R2,500 pm	No	one	Yes	one	Yes	No	No	24	No	42	No	2.1
20-24	Yes	Yes	No	R0 - R500 pm	No	one	Yes	one	No	No	No	25	No	11	Yes	0.8
15-19	No	Yes	Yes	R501 - R2,500 pm	Yes	one	Yes	one	No	No	No	19	Yes	22	No	1.8
15-19	No	Yes	Yes	R501 - R2,500 pm	Yes	one	Yes	one	No	Yes	No	24	No	13	No	1.9
15-19	No	Yes	No	R501 - R2,500 pm	Yes	one	Yes	one	No	No	No	24	No	27	No	1.5
20-24	Yes	Yes	Yes	R2,501 - R6,000 pm	Yes	one	Yes	one	No	Yes	No	30	No	11	No	1.8
20-24	Yes	Yes	Yes	R501 - R2,500 pm	No	one	Yes	one	No	No	No	25	No	9	No	2
20-24	No	No	No	R501 - R2,500 pm	Yes	one	Yes	one	No	No	No	27	No	52	No	1.4
20-24	Yes	Yes	Yes	R2,501 - R6,000 pm	Yes	one	Yes	one	Yes	Yes	No	23	No	15	No	1.2
20-24	No	No	Yes	.	Yes	one	Yes	one	No	No	No	23	No	20	No	1.5
20-24	Yes	No	Yes	R2,501 - R6,000 pm	Yes	one	No	one	No	No	No	34	No	16	No	1.5
20-24	No	No	No	R501 - R2,500 pm	Yes	one	Yes	none	.	Yes	No	25	.	91	No	1.3

20-24	No	Yes	No	R501 - R2,500 pm	No	one	Yes	2 or more	No	No	No	26	No	13	No	1.6
15-19	Yes	Yes	Yes	greater than R6,000 pm	Yes	one	Yes	one	Yes	Yes	No	21	No	54	No	1.8
15-19	Yes	Yes	Yes	greater than R6,000 pm	Yes	one	No	one	Yes	Yes	No	25	No	15	No	1.6
20-24	Yes	No	Yes	R501 - R2,500 pm	Yes	one	No	one	No	Yes	No	21	No	26	No	1.5
20-24	Yes	Yes	Yes	R501 - R2,500 pm	Yes	one	No	one	Yes	Yes	No	33	No	12	No	2
20-24	Yes	Yes	Yes	R501 - R2,500 pm	No	one	Yes	one	No	Yes	No	25	No	9	No	2.1
15-19	No	Yes	Yes	R501 - R2,500 pm	Yes	one	Yes	none	.	No	No	22	.	23	No	1.6
20-24	Yes	No	Yes	R501 - R2,500 pm	No	one	Yes	one	No	No	No	24	No	26	No	2
15-19	No	Yes	Yes	.	No	one	Yes	none	.	Yes	No	18	.	30	No	2
20-24	Yes	Yes	No	R501 - R2,500 pm	No	one	No	one	No	Yes	No	29	No	11	No	1.6
15-19	No	No	Yes	R2,501 - R6,000 pm	No	one	Yes	one	No	No	No	20	No	50	No	1.9
15-19	No	Yes	Yes	R0 - R500 pm	No	one	Yes	one	No	No	No	23	No	9	No	1.9
20-24	No	Yes	Yes	R0 - R500 pm	Yes	one	Yes	one	No	Yes	No	27	No	24	No	1.8
20-24	Yes	No	Yes	R501 - R2,500 pm	Yes	one	Yes	one	Yes	No	No	28	No	21	No	1.5
15-19	No	Yes	Yes	R501 - R2,500 pm	No	one	No	one	Yes	Yes	No	21	No	10	No	1.1
15-19	Yes	Yes	Yes	R501 - R2,500 pm	Yes	one	Yes	one	Yes	No	No	33	No	15	No	1.8
15-19	Yes	No	Yes	R501 - R2,500 pm	No	one	No	one	No	No	No	27	No	13	No	1.3
15-19	Yes	Yes	Yes	R501 - R2,500 pm	No	one	Yes	one	No	No	No	23	No	8	No	2.1
20-24	No	Yes	Yes	R501 - R2,500 pm	Yes	one	Yes	one	No	No	No	28	No	23	No	2
15-19	No	Yes	Yes	R501 - R2,500 pm	No	one	Yes	2 or more	No	No	Yes	25	No	12	No	1.3
15-19	No	Yes	Yes	R2,501 - R6,000 pm	Yes	one	Yes	one	No	No	No	27	No	8	No	1.9
15-19	No	Yes	No	R0 - R500 pm	Yes	one	Yes	2 or more	No	Yes	No	28	No	39	No	1.2
20-24	Yes	Yes	Yes	R2,501 - R6,000 pm	Yes	one	Yes	one	No	Yes	No	25	No	24	No	1.9
15-19	Yes	No	Yes	R501 - R2,500 pm	Yes	one	No	one	No	No	No	23	No	7	No	1.3
20-24	Yes	Yes	Yes	R501 - R2,500 pm	No	one	Yes	one	Yes	Yes	No	28	No	10	No	1.6
15-19	No	No	Yes	R501 - R2,500 pm	Yes	one	Yes	none	.	Yes	No	21	.	65	No	1.3
20-24	Yes	No	Yes	R501 - R2,500 pm	Yes	one	Yes	one	Yes	No	No	30	No	46	No	1.2
20-24	No	Yes	Yes	R501 - R2,500 pm	No	one	No	one	No	No	No	26	Yes	12	No	2.5
15-19	No	Yes	Yes	R501 - R2,500 pm	No	one	No	one	No	No	No	34	No	49	No	2
15-19	No	Yes	Yes	R2,501 - R6,000 pm	No	one	Yes	none	.	.	.	.	.	10	Yes	1
15-19	No	Yes	Yes	greater than R6,000 pm	Yes	one	Yes	one	No	No	No	22	No	27	Yes	0.8
15-19	No	Yes	Yes	R0 - R500 pm	Yes	one	Yes	one	Yes	No	No	21	No	9	No	1.2
20-24	Yes	Yes	No	R0 - R500 pm	No	one	Yes	one	No	No	No	27	No	9	No	1.8
20-24	Yes	Yes	No	R501 - R2,500 pm	Yes	one	Yes	one	No	No	No	28	No	57	No	1.9
20-24	No	Yes	Yes	greater than R6,000 pm	Yes	one	No	none	.	.	.	.	.	20	No	1.2
15-19	Yes	Yes	No	.	No	one	Yes	one	No	Yes	No	24	No	52	No	1.9
20-24	No	Yes	Yes	R501 - R2,500 pm	Yes	one	Yes	one	No	Yes	No	23	No	7	No	1.2
15-19	Yes	No	Yes	R0 - R500 pm	Yes	one	Yes	one	No	Yes	No	26	No	41	No	1.5
15-19	Yes	Yes	Yes	R501 - R2,500 pm	Yes	one	No	one	No	No	No	21	No	21	Yes	0.6
20-24	Yes	Yes	No	R0 - R500 pm	No	one	No	one	No	No	No	24	No	11	No	2.3
15-19	No	Yes	Yes	R2,501 - R6,000 pm	No	one	Yes	none	.	.	.	.	.	11	No	1.4
15-19	No	Yes	Yes	R0 - R500 pm	No	one	Yes	none	.	Yes	No	22	.	11	No	1.2

15-19	No	No	Yes	R501 - R2,500 pm	No	one	Yes	one	Yes	No	No	22	No	31	No	1.4
20-24	Yes	Yes	Yes	R501 - R2,500 pm	No	one	Yes	none	.	.	.	.	.	19	No	1.5
20-24	Yes	No	No	.	Yes	one	Yes	one	No	No	No	31	Yes	27	No	1.7
15-19	Yes	Yes	Yes	R501 - R2,500 pm	Yes	one	No	one	No	Yes	No	25	No	15	No	1.6
15-19	Yes	Yes	Yes	R2,501 - R6,000 pm	No	one	Yes	one	No	No	No	25	No	10	No	1.2
15-19	No	No	Yes	R501 - R2,500 pm	Yes	one	Yes	one	No	Yes	No	25	No	28	No	1.5
20-24	No	No	Yes	R501 - R2,500 pm	Yes	one	Yes	one	Yes	No	No	26	No	26	Yes	0.7
20-24	No	Yes	Yes	R2,501 - R6,000 pm	Yes	one	Yes	one	No	No	No	28	No	20	No	1.3
20-24	No	Yes	Yes	R501 - R2,500 pm	Yes	one	Yes	one	No	Yes	No	28	No	19	No	2
20-24	No	No	Yes	R2,501 - R6,000 pm	Yes	one	Yes	one	No	Yes	No	29	No	17	No	2.2
20-24	Yes	No	Yes	R501 - R2,500 pm	No	one	Yes	none	.	No	No	26	.	26	No	1.5
20-24	Yes	No	No	R501 - R2,500 pm	Yes	one	Yes	one	No	Yes	No	29	No	50	No	1.5
15-19	No	Yes	No	.	Yes	one	No	one	Yes	No	No	24	No	14	No	1.9
15-19	Yes	No	Yes	R501 - R2,500 pm	Yes	one	Yes	one	No	No	No	25	No	33	No	1.4
15-19	No	No	Yes	R501 - R2,500 pm	Yes	one	Yes	one	No	No	No	20	No	90	No	1.5
15-19	Yes	Yes	Yes	.	Yes	one	No	2 or more	No	No	No	23	No	9	No	1.5
15-19	Yes	Yes	Yes	R2,501 - R6,000 pm	Yes	one	Yes	one	No	Yes	No	22	No	10	No	1.8
20-24	Yes	Yes	Yes	greater than R6,000 pm	Yes	one	Yes	one	No	No	No	35	No	18	No	1.3
20-24	Yes	No	Yes	R501 - R2,500 pm	Yes	one	No	one	No	No	No	27	No	16	No	1.8
20-24	Yes	Yes	Yes	R501 - R2,500 pm	Yes	one	Yes	one	Yes	Yes	No	28	No	20	No	2.3
20-24	No	No	Yes	R0 - R500 pm	Yes	one	Yes	one	No	Yes	No	26	No	24	No	1.3
20-24	Yes	No	Yes	R2,501 - R6,000 pm	Yes	one	Yes	one	No	Yes	No	24	No	12	No	1.2
20-24	Yes	No	Yes	R0 - R500 pm	No	one	Yes	2 or more	.	No	No	30	No	30	No	1.5
15-19	No	Yes	Yes	R501 - R2,500 pm	Yes	one	Yes	one	No	No	No	25	Yes	18	Yes	0.6
20-24	Yes	Yes	Yes	R2,501 - R6,000 pm	Yes	one	Yes	one	No	.	No	27	No	27	No	1.4
15-19	No	Yes	Yes	R501 - R2,500 pm	Yes	one	No	one	Yes	Yes	No	21	No	20	No	1.3
20-24	Yes	Yes	Yes	greater than R6,000 pm	No	one	Yes	2 or more	No	Yes	No	25	No	16	Yes	1
15-19	No	Yes	Yes	R0 - R500 pm	Yes	one	Yes	one	No	No	No	22	No	12	No	2
20-24	Yes	Yes	No	R0 - R500 pm	Yes	one	Yes	2 or more	No	No	No	28	No	31	No	1.3
20-24	Yes	No	Yes	R0 - R500 pm	Yes	one	Yes	one	No	No	No	28	No	87	No	1.3
20-24	No	No	Yes	greater than R6,000 pm	Yes	one	Yes	one	No	No	No	24	No	30	No	1.7
15-19	No	No	Yes	R501 - R2,500 pm	No	one	No	none	.	No	No	21	.	23	No	1.3
15-19	Yes	Yes	Yes	R2,501 - R6,000 pm	Yes	one	No	one	No	No	No	24	No	17	No	2.2
15-19	No	Yes	Yes	R501 - R2,500 pm	No	one	No	2 or more	No	Yes	No	23	No	21	Yes	0.8
20-24	No	Yes	No	R501 - R2,500 pm	Yes	one	Yes	one	No	Yes	No	39	No	10	No	2.4
20-24	Yes	No	Yes	R501 - R2,500 pm	Yes	one	.	one	Yes	Yes	No	36	No	16	No	1.8
15-19	Yes	Yes	Yes	R501 - R2,500 pm	Yes	one	No	one	No	.	No	23	No	12	Yes	0.9
20-24	Yes	Yes	No	R0 - R500 pm	Yes	one	Yes	2 or more	.	No	No	29	No	18	No	1.4
20-24	Yes	No	Yes	greater than R6,000 pm	Yes	one	Yes	one	Yes	Yes	No	25	No	47	No	1.6
15-19	Yes	Yes	Yes	greater than R6,000 pm	No	one	No	one	No	Yes	No	25	No	32	No	1.2
20-24	Yes	Yes	Yes	R501 - R2,500 pm	No	one	No	one	No	Yes	No	27	No	11	No	2

20-24	No	Yes	No	.	Yes	one	Yes	one	No	No	No	29	No	14	No	1.7
20-24	Yes	No	No	R2,501 - R6,000 pm	Yes	one	Yes	one	Yes	No	No	24	No	12	No	1.2
20-24	No	No	Yes	greater than R6,000 pm	Yes	one	Yes	one	Yes	Yes	No	25	No	56	No	1.3
15-19	Yes	Yes	Yes	.	Yes	one	No	one	No	No	No	21	No	13	No	1.2
15-19	Yes	No	No	R2,501 - R6,000 pm	No	one	No	one	No	No	No	28	No	25	Yes	0.9
15-19	Yes	Yes	Yes	R501 - R2,500 pm	Yes	one	Yes	one	No	No	No	26	No	12	No	2.2
20-24	Yes	Yes	No	R501 - R2,500 pm	Yes	one	.	one	No	Yes	No	30	No	17	No	1.9
20-24	Yes	No	Yes	R501 - R2,500 pm	Yes	one	Yes	one	No	Yes	No	30	No	59	No	1.4
15-19	No	Yes	Yes	R501 - R2,500 pm	No	one	Yes	2 or more	No	No	No	32	No	12	No	1.3
20-24	No	Yes	Yes	R501 - R2,500 pm	Yes	one	No	one	No	.	No	30	No	9	No	1.8
15-19	No	Yes	Yes	R501 - R2,500 pm	Yes	one	.	one	No	Yes	No	21	No	58	No	1.3
15-19	Yes	Yes	Yes	R2,501 - R6,000 pm	Yes	one	Yes	one	No	No	No	23	No	23	No	1.7
20-24	Yes	Yes	No	greater than R6,000 pm	Yes	one	Yes	none	.	.	No	23	.	14	No	1.2
20-24	Yes	Yes	Yes	R501 - R2,500 pm	Yes	one	Yes	one	Yes	No	No	25	No	12	No	2.3
15-19	Yes	Yes	No	R501 - R2,500 pm	No	one	Yes	one	No	No	No	26	No	32	No	1.9
15-19	No	Yes	Yes	R2,501 - R6,000 pm	No	one	Yes	none	.	Yes	No	18	.	34	No	1.6
20-24	Yes	Yes	Yes	R501 - R2,500 pm	No	one	Yes	one	No	Yes	No	24	No	23	No	1.6
20-24	No	No	Yes	R501 - R2,500 pm	Yes	one	Yes	one	No	Yes	No	32	No	50	No	1.5
15-19	Yes	Yes	Yes	R501 - R2,500 pm	Yes	one	Yes	one	No	No	No	30	No	19	No	1.9
15-19	No	Yes	Yes	R0 - R500 pm	No	one	No	none	.	Yes	No	18	.	30	No	1.7
20-24	Yes	No	No	R2,501 - R6,000 pm	Yes	one	Yes	2 or more	No	No	No	23	Yes	32	No	1.4
20-24	Yes	Yes	No	R501 - R2,500 pm	Yes	one	Yes	one	No	No	No	27	No	28	No	1.9
20-24	No	No	No	R501 - R2,500 pm	Yes	one	Yes	one	No	No	No	25	No	11	No	1.2
20-24	Yes	Yes	No	R501 - R2,500 pm	Yes	one	Yes	one	No	No	No	27	No	14	No	1.7
15-19	No	No	No	R0 - R500 pm	Yes	one	No	none	.	.	No	18	.	53	No	1.3
15-19	No	Yes	Yes	R2,501 - R6,000 pm	No	one	No	one	No	Yes	No	18	Yes	50	No	1.4
20-24	No	No	No	R0 - R500 pm	Yes	one	Yes	one	No	No	No	32	No	17	No	2.3
15-19	Yes	No	Yes	greater than R6,000 pm	Yes	one	Yes	one	No	No	No	23	No	63	No	1.6
15-19	No	Yes	Yes	R501 - R2,500 pm	Yes	one	Yes	one	No	No	No	26	No	23	No	1.7
20-24	No	No	Yes	R501 - R2,500 pm	No	one	Yes	none	.	No	No	25	.	68	No	1.3
20-24	No	Yes	No	R501 - R2,500 pm	Yes	one	Yes	2 or more	Yes	No	Yes	28	No	30	Yes	0.7
20-24	Yes	Yes	Yes	R2,501 - R6,000 pm	Yes	one	Yes	one	No	No	No	26	No	36	No	1.3
15-19	Yes	Yes	Yes	R501 - R2,500 pm	Yes	one	Yes	one	No	Yes	No	22	No	15	No	1.7
15-19	No	Yes	Yes	greater than R6,000 pm	No	one	No	none	.	.	.	.	.	11	No	1.6
20-24	Yes	No	Yes	R501 - R2,500 pm	Yes	one	No	one	Yes	No	No	26	No	91	No	1.5
15-19	No	Yes	No	greater than R6,000 pm	No	one	Yes	one	No	Yes	Yes	28	No	12	No	1.6
15-19	Yes	Yes	No	R0 - R500 pm	No	one	Yes	2 or more	No	No	No	29	No	12	No	1.8
20-24	Yes	No	Yes	R501 - R2,500 pm	Yes	one	Yes	one	Yes	Yes	No	24	No	26	No	1.2
20-24	No	No	Yes	R501 - R2,500 pm	Yes	one	Yes	one	No	No	No	26	Yes	42	No	2
15-19	No	No	Yes	R501 - R2,500 pm	No	one	Yes	one	Yes	Yes	No	29	No	18	No	1.3
15-19	Yes	Yes	Yes	R501 - R2,500 pm	Yes	one	Yes	one	No	No	No	25	No	30	No	1.3

20-24	No	No	Yes	R2,501 - R6,000 pm	No	one	No	none	.	.	No	21	.	25	No	1.3
20-24	Yes	Yes	Yes	R0 - R500 pm	Yes	one	Yes	one	No	No	Yes	25	No	14	Yes	0.9
20-24	Yes	Yes	Yes	R2,501 - R6,000 pm	Yes	one	No	one	Yes	No	No	23	No	10	No	1.7
20-24	Yes	Yes	No	.	No	one	Yes	one	No	Yes	No	26	No	12	No	2.2
15-19	No	Yes	Yes	R501 - R2,500 pm	Yes	one	Yes	one	No	No	No	21	Yes	16	No	1.2
20-24	Yes	No	Yes	R501 - R2,500 pm	Yes	one	Yes	one	No	No	No	35	Yes	23	No	1.4
15-19	No	Yes	Yes	R501 - R2,500 pm	Yes	one	No	one	No	Yes	No	23	No	26	No	1.2
20-24	Yes	Yes	Yes	R501 - R2,500 pm	Yes	one	Yes	one	No	No	No	24	No	21	No	1.2
15-19	No	No	Yes	.	Yes	one	No	one	No	Yes	No	20	No	17	No	1.4
15-19	No	No	Yes	R501 - R2,500 pm	Yes	one	No	none	.	.	.	.	.	106	No	1.5
20-24	No	No	Yes	R2,501 - R6,000 pm	No	one	No	one	No	No	No	29	No	91	No	1.3
20-24	Yes	Yes	Yes	R2,501 - R6,000 pm	Yes	one	No	one	No	No	No	24	Yes	14	No	1.3
20-24	Yes	Yes	No	R501 - R2,500 pm	No	one	No	one	Yes	.	No	31	No	20	No	1.9
20-24	Yes	Yes	No	R0 - R500 pm	Yes	one	Yes	one	No	No	No	26	No	9	No	1.8
15-19	No	No	Yes	R501 - R2,500 pm	No	one	No	none	.	Yes	No	18	.	17	Yes	1
15-19	Yes	Yes	Yes	R501 - R2,500 pm	No	one	Yes	one	No	No	No	25	No	15	No	1.6
20-24	Yes	No	Yes	R2,501 - R6,000 pm	Yes	one	No	one	No	Yes	No	26	No	54	No	1.4
20-24	Yes	No	Yes	R501 - R2,500 pm	Yes	one	Yes	one	No	Yes	No	25	No	22	No	1.3
20-24	Yes	Yes	Yes	R501 - R2,500 pm	Yes	one	Yes	one	Yes	Yes	No	25	No	13	No	1.9
15-19	No	No	Yes	R2,501 - R6,000 pm	No	one	No	one	No	No	No	20	No	48	No	1.3
20-24	No	Yes	Yes	R501 - R2,500 pm	Yes	one	Yes	one	No	No	No	28	No	13	No	2.5
20-24	No	No	Yes	R501 - R2,500 pm	Yes	one	Yes	none	.	No	No	24	.	9	No	1.8
15-19	No	Yes	Yes	R2,501 - R6,000 pm	Yes	one	No	one	No	No	No	19	No	27	No	1.4
20-24	Yes	Yes	Yes	R501 - R2,500 pm	No	one	Yes	one	No	No	No	25	No	7	No	1.9
20-24	Yes	No	Yes	R501 - R2,500 pm	Yes	one	No	one	No	Yes	No	26	No	44	No	1.4
15-19	No	Yes	Yes	R2,501 - R6,000 pm	Yes	one	No	2 or more	No	No	No	22	No	11	No	1.9
20-24	Yes	Yes	Yes	R501 - R2,500 pm	Yes	one	Yes	one	No	Yes	No	25	No	30	No	1.1
20-24	Yes	Yes	Yes	.	No	one	No	one	No	Yes	No	23	No	35	Yes	1
15-19	No	Yes	Yes	R501 - R2,500 pm	No	one	Yes	one	No	Yes	No	26	No	29	No	1.2
20-24	Yes	Yes	Yes	R0 - R500 pm	No	one	No	one	No	Yes	No	28	No	12	No	1.8
15-19	No	No	Yes	R0 - R500 pm	Yes	one	No	none	.	Yes	No	18	.	7	No	1.3
15-19	Yes	Yes	Yes	R2,501 - R6,000 pm	Yes	one	No	one	Yes	No	No	23	No	26	Yes	0.6
20-24	No	Yes	No	R501 - R2,500 pm	Yes	one	Yes	one	No	No	No	26	No	14	No	1.7
20-24	Yes	No	No	.	Yes	one	Yes	one	No	Yes	No	25	No	27	No	1.7
15-19	No	No	Yes	R501 - R2,500 pm	No	one	No	one	No	Yes	No	18	No	33	No	2
20-24	Yes	No	Yes	R2,501 - R6,000 pm	Yes	one	Yes	one	No	Yes	No	30	No	47	No	1.5
20-24	Yes	No	Yes	R501 - R2,500 pm	Yes	one	Yes	one	No	Yes	No	34	No	65	No	1.3
15-19	Yes	Yes	Yes	greater than R6,000 pm	Yes	one	Yes	one	No	Yes	No	23	Yes	65	No	1.3
15-19	No	No	Yes	R0 - R500 pm	No	one	No	one	No	No	No	30	No	101	No	1.8
15-19	Yes	Yes	Yes	greater than R6,000 pm	Yes	one	No	one	No	Yes	No	31	No	26	No	2.2
15-19	No	Yes	Yes	R501 - R2,500 pm	Yes	one	Yes	one	No	No	No	25	No	12	No	1.4
20-24	Yes	Yes	Yes	R0 - R500 pm	No	one	No	one	No	Yes	No	22	Yes	8	No	1.8
15-19	No	Yes	Yes	R501 - R2,500 pm	No	one	Yes	none	.	No	Yes	23	.	11	No	1.8
20-24	Yes	Yes	Yes	R501 - R2,500 pm	Yes	one	Yes	one	No	Yes	No	25	No	10	No	1.9

20-24	Yes	No	Yes	R2,501 - R6,000 pm	No	one	No	one	Yes	Yes	No	29	No	87	No	1.5
20-24	Yes	No	Yes	R501 - R2,500 pm	Yes	one	No	one	No	Yes	No	28	No	88	No	1.5
15-19	Yes	No	Yes	R2,501 - R6,000 pm	No	one	No	one	No	Yes	No	23	No	106	No	1.9
20-24	Yes	No	Yes	greater than R6,000 pm	Yes	one	Yes	one	Yes	No	No	24	No	49	No	1.5
15-19	Yes	Yes	Yes	R2,501 - R6,000 pm	No	one	No	one	No	Yes	No	23	No	9	No	1.9
15-19	Yes	Yes	Yes	R0 - R500 pm	No	one	No	one	Yes	Yes	No	18	No	10	No	1.5
20-24	Yes	No	Yes	R2,501 - R6,000 pm	Yes	one	Yes	none	.	Yes	No	27	.	60	No	1.5
20-24	No	No	Yes	R501 - R2,500 pm	Yes	one	No	one	No	Yes	No	25	No	87	Yes	0.9
15-19	Yes	Yes	Yes	R501 - R2,500 pm	Yes	one	Yes	one	No	No	No	24	No	27	Yes	1.1
15-19	No	No	Yes	R2,501 - R6,000 pm	No	one	No	one	Yes	Yes	No	19	No	17	No	1.8
20-24	Yes	No	Yes	R2,501 - R6,000 pm	No	one	No	one	No	No	No	26	No	78	No	1.3
20-24	No	Yes	Yes	R0 - R500 pm	No	one	Yes	2 or more	No	No	No	31	Yes	12	No	1.7
20-24	Yes	Yes	No	R501 - R2,500 pm	Yes	one	No	one	No	Yes	No	26	No	14	No	2.1
20-24	Yes	Yes	Yes	greater than R6,000 pm	Yes	one	No	one	No	No	No	24	No	40	No	1.2
20-24	No	No	Yes	R2,501 - R6,000 pm	Yes	one	Yes	2 or more	.	No	No	28	Yes	48	No	1.3
20-24	No	Yes	Yes	R2,501 - R6,000 pm	Yes	one	Yes	one	No	No	No	25	No	52	No	1.1
15-19	No	Yes	Yes	R501 - R2,500 pm	Yes	one	Yes	one	No	No	No	27	No	20	No	2.1
20-24	Yes	Yes	Yes	R501 - R2,500 pm	No	one	No	one	Yes	Yes	No	30	No	8	No	2
20-24	Yes	Yes	No	R501 - R2,500 pm	Yes	one	Yes	one	Yes	No	No	29	No	13	No	1.2
20-24	No	Yes	Yes	R501 - R2,500 pm	No	one	Yes	one	No	.	No	28	No	12	No	1.8
15-19	Yes	Yes	Yes	R2,501 - R6,000 pm	Yes	one	No	one	No	Yes	No	22	No	34	No	1.5
15-19	Yes	Yes	Yes	R2,501 - R6,000 pm	Yes	one	No	2 or more	No	Yes	No	23	No	15	No	1.1
20-24	No	No	No	R501 - R2,500 pm	Yes	one	Yes	one	No	Yes	No	25	No	29	No	1.8
20-24	Yes	No	Yes	R2,501 - R6,000 pm	Yes	one	No	one	No	Yes	No	37	No	26	No	1.4
15-19	Yes	No	Yes	R501 - R2,500 pm	Yes	one	Yes	none	.	Yes	No	20	.	47	Yes	0.8
15-19	No	No	Yes	R2,501 - R6,000 pm	Yes	one	Yes	one	No	No	No	23	No	52	No	1.3
15-19	Yes	No	Yes	R501 - R2,500 pm	Yes	one	No	one	No	No	No	25	No	102	Yes	1.3
20-24	Yes	No	Yes	R0 - R500 pm	Yes	one	Yes	one	Yes	Yes	No	22	No	52	No	1.3
20-24	Yes	Yes	Yes	R501 - R2,500 pm	Yes	one	No	one	No	Yes	No	30	No	11	No	1.8
20-24	No	Yes	Yes	R501 - R2,500 pm	Yes	one	Yes	one	No	Yes	No	27	No	30	No	1.3
15-19	Yes	Yes	Yes	.	Yes	one	No	one	No	Yes	No	28	Yes	23	No	1.8
20-24	No	Yes	Yes	R501 - R2,500 pm	Yes	one	Yes	one	No	Yes	No	21	No	24	No	1.4
20-24	Yes	Yes	Yes	R501 - R2,500 pm	No	one	Yes	one	No	Yes	No	34	No	9	No	1.2
20-24	Yes	No	Yes	R0 - R500 pm	Yes	one	Yes	one	No	No	No	24	No	63	No	1.3
20-24	Yes	No	Yes	.	Yes	one	Yes	one	Yes	Yes	No	27	Yes	24	No	1.9
20-24	Yes	Yes	Yes	R2,501 - R6,000 pm	No	one	Yes	none	.	.	.	.	.	8	No	1.9
20-24	Yes	Yes	Yes	.	Yes	one	No	one	Yes	Yes	No	25	No	8	No	1.6
15-19	Yes	No	Yes	R2,501 - R6,000 pm	Yes	one	No	2 or more	No	No	No	22	Yes	71	No	1.3
15-19	No	Yes	Yes	R501 - R2,500 pm	No	one	No	one	No	Yes	No	22	No	23	No	2.2
20-24	No	No	No	R0 - R500 pm	No	one	Yes	one	No	No	No	27	No	51	No	1.3
15-19	No	Yes	Yes	R2,501 - R6,000 pm	No	one	No	one	No	No	No	22	No	11	No	1.8
20-24	Yes	No	Yes	R501 - R2,500 pm	Yes	one	No	one	No	No	No	31	No	45	No	1.7
20-24	Yes	Yes	No	R2,501 - R6,000 pm	Yes	one	Yes	one	Yes	Yes	No	23	No	20	No	1.3
15-19	Yes	No	Yes	R501 - R2,500 pm	No	one	Yes	one	No	Yes	No	20	No	65	No	1.4

20-24	Yes	No	Yes	R501 - R2,500 pm	Yes	one	Yes	one	No	No	No	33	No	25	No	2.3
15-19	Yes	Yes	Yes	R2,501 - R6,000 pm	Yes	one	No	none	.	Yes	No	21	.	49	No	1.7
15-19	No	No	Yes	R501 - R2,500 pm	Yes	one	No	one	No	Yes	No	20	No	49	Yes	0.7
20-24	Yes	Yes	Yes	R501 - R2,500 pm	Yes	one	Yes	one	No	Yes	No	24	No	18	No	1.2
15-19	Yes	Yes	Yes	R501 - R2,500 pm	Yes	one	No	one	No	Yes	No	19	No	47	No	1.4
15-19	No	Yes	Yes	R2,501 - R6,000 pm	No	one	No	one	No	No	No	25	No	26	No	1.9
20-24	No	No	Yes	R501 - R2,500 pm	Yes	one	Yes	one	No	Yes	No	23	No	15	No	1.1
15-19	No	Yes	Yes	R2,501 - R6,000 pm	Yes	one	No	one	No	Yes	No	21	No	11	No	1.5
15-19	Yes	Yes	Yes	R2,501 - R6,000 pm	Yes	one	Yes	one	No	Yes	No	24	No	21	No	1.3
15-19	No	Yes	Yes	greater than R6,000 pm	Yes	one	No	none	.	No	No	18	.	21	Yes	0.7
20-24	Yes	Yes	Yes	R501 - R2,500 pm	Yes	one	Yes	none	.	.	No	24	.	11	No	1.2
15-19	Yes	Yes	Yes	R501 - R2,500 pm	Yes	one	Yes	one	No	Yes	No	25	No	9	No	1.7
15-19	No	Yes	Yes	R2,501 - R6,000 pm	No	one	No	one	Yes	Yes	No	20	Yes	24	No	1.6
20-24	Yes	Yes	No	R2,501 - R6,000 pm	Yes	one	Yes	one	No	Yes	No	35	No	14	No	1.7
15-19	No	Yes	Yes	R501 - R2,500 pm	Yes	one	No	none	.	Yes	No	19	.	24	No	1.3
20-24	Yes	Yes	Yes	R501 - R2,500 pm	No	one	No	one	No	Yes	No	23	No	26	Yes	0.6
20-24	Yes	Yes	Yes	R2,501 - R6,000 pm	Yes	one	Yes	one	No	No	No	31	No	28	No	1.3
15-19	No	Yes	Yes	R2,501 - R6,000 pm	Yes	one	No	one	No	.	No	28	Yes	19	No	2
20-24	No	Yes	No	R501 - R2,500 pm	Yes	one	Yes	one	No	Yes	No	41	No	10	No	1.7
15-19	No	Yes	Yes	R501 - R2,500 pm	Yes	one	Yes	one	Yes	Yes	No	23	No	27	No	1.7
20-24	Yes	Yes	Yes	R2,501 - R6,000 pm	Yes	one	No	one	No	Yes	No	24	No	21	No	2.1
20-24	Yes	Yes	Yes	R2,501 - R6,000 pm	No	one	No	one	No	Yes	No	28	No	49	No	2
20-24	Yes	Yes	Yes	R2,501 - R6,000 pm	Yes	one	No	one	No	Yes	No	27	Yes	12	No	1.5
15-19	Yes	Yes	Yes	R0 - R500 pm	Yes	one	No	one	No	No	No	20	No	10	No	1.5
20-24	No	Yes	No	R0 - R500 pm	Yes	one	Yes	one	No	No	No	24	Yes	15	No	1.3
20-24	Yes	Yes	Yes	R0 - R500 pm	Yes	one	Yes	one	Yes	No	No	23	No	15	No	1.3
15-19	No	Yes	Yes	R2,501 - R6,000 pm	No	one	No	one	No	No	No	24	No	18	No	1.3
15-19	No	Yes	Yes	R501 - R2,500 pm	No	one	No	one	No	No	No	19	No	38	No	1.4
15-19	No	Yes	Yes	R501 - R2,500 pm	Yes	one	Yes	one	Yes	No	No	20	No	25	No	1.3
20-24	Yes	Yes	Yes	R501 - R2,500 pm	Yes	one	Yes	one	No	Yes	No	35	No	20	No	1.7
20-24	No	Yes	No	R0 - R500 pm	Yes	one	Yes	one	No	No	No	30	No	20	No	1.7
15-19	No	Yes	No	R2,501 - R6,000 pm	No	one	No	one	No	Yes	No	19	No	16	No	1.3
15-19	Yes	Yes	Yes	R501 - R2,500 pm	No	one	Yes	one	No	No	No	20	No	31	No	1.3
15-19	No	Yes	Yes	R0 - R500 pm	Yes	one	No	one	Yes	No	No	21	No	20	No	1.5
20-24	No	No	Yes	R501 - R2,500 pm	Yes	one	Yes	one	Yes	Yes	No	33	No	21	No	2.1
20-24	Yes	No	Yes	R2,501 - R6,000 pm	Yes	one	Yes	one	No	Yes	No	23	No	21	No	2.1
15-19	No	No	Yes	R501 - R2,500 pm	No	one	Yes	one	Yes	No	No	20	No	16	No	1.8
15-19	Yes	No	Yes	greater than R6,000 pm	Yes	one	Yes	one	No	Yes	No	29	Yes	24	No	1.8
20-24	Yes	No	Yes	R2,501 - R6,000 pm	Yes	one	No	one	No	Yes	No	22	No	14	No	1.9
15-19	No	No	Yes	R2,501 - R6,000 pm	Yes	one	No	one	No	Yes	No	22	No	28	No	1.7
15-19	No	No	Yes	R2,501 - R6,000 pm	Yes	one	Yes	one	No	Yes	No	26	No	36	No	1.8
20-24	Yes	No	Yes	R501 - R2,500 pm	Yes	one	Yes	2 or more	No	No	No	36	No	20	No	1.7
20-24	Yes	No	Yes	R2,501 - R6,000 pm	No	one	Yes	one	No	No	No	28	No	59	No	1.8
20-24	No	No	Yes	R2,501 - R6,000 pm	Yes	one	Yes	one	No	No	No	36	No	53	No	1.9

20-24	No	No	Yes	R2,501 - R6,000 pm	Yes	one	Yes	one	No	No	No	34	No	53	No	1.8
15-19	No	No	Yes	R2,501 - R6,000 pm	Yes	one	No	none	.	.	No	17	.	29	No	1.3
20-24	Yes	No	Yes	R2,501 - R6,000 pm	Yes	one	Yes	one	No	No	No	25	No	30	No	1.2
15-19	No	No	Yes	R2,501 - R6,000 pm	No	one	No	one	No	No	No	19	No	18	No	1.3
20-24	Yes	No	Yes	R501 - R2,500 pm	Yes	one	Yes	one	No	No	No	26	No	24	No	1.2
20-24	Yes	No	Yes	R2,501 - R6,000 pm	Yes	one	Yes	one	No	No	No	23	No	12	No	1.2
15-19	Yes	No	Yes	R501 - R2,500 pm	No	one	No	one	No	Yes	No	23	No	25	No	1.7
20-24	Yes	No	Yes	R2,501 - R6,000 pm	Yes	one	Yes	one	Yes	Yes	No	27	No	59	No	1.7
15-19	No	No	Yes	R501 - R2,500 pm	No	one	Yes	one	Yes	Yes	Yes	21	No	51	No	1.5
15-19	No	No	No	R501 - R2,500 pm	No	one	Yes	one	No	Yes	No	23	No	17	No	1.2
20-24	Yes	No	Yes	R501 - R2,500 pm	No	one	No	one	No	Yes	No	31	No	36	No	1.2
20-24	No	No	Yes	R2,501 - R6,000 pm	Yes	one	Yes	one	No	No	No	29	No	39	No	1.5
20-24	Yes	No	No	greater than R6,000 pm	Yes	one	Yes	one	No	No	No	32	No	52	No	1.4
20-24	Yes	No	Yes	R2,501 - R6,000 pm	Yes	one	Yes	one	No	Yes	No	25	No	52	No	1.4
20-24	No	No	Yes	R0 - R500 pm	No	one	No	one	No	No	No	26	No	31	No	1.7
20-24	Yes	No	Yes	R501 - R2,500 pm	No	one	Yes	one	No	No	No	30	No	58	No	1.7
20-24	Yes	No	Yes	R2,501 - R6,000 pm	No	one	No	one	No	Yes	No	23	No	44	No	1.7
15-19	No	No	Yes	R2,501 - R6,000 pm	Yes	one	Yes	one	No	Yes	No	25	No	40	No	1.7
20-24	No	No	Yes	R501 - R2,500 pm	Yes	one	Yes	none	.	Yes	No	29	.	44	No	1.7
20-24	Yes	No	Yes	R2,501 - R6,000 pm	No	one	No	one	No	Yes	No	23	No	60	Yes	0.7
15-19	No	No	Yes	R2,501 - R6,000 pm	No	one	Yes	one	No	No	No	23	No	34	No	1.5
20-24	No	No	Yes	R501 - R2,500 pm	Yes	one	Yes	one	No	No	No	37	No	37	No	1.5
20-24	Yes	No	Yes	R2,501 - R6,000 pm	Yes	one	Yes	one	Yes	Yes	No	28	No	13	No	1.8
20-24	Yes	No	Yes	greater than R6,000 pm	Yes	one	Yes	none	.	No	No	26	.	36	No	1.3
15-19	Yes	No	No	R2,501 - R6,000 pm	No	one	No	none	.	No	No	27	.	13	No	1.9
20-24	No	No	No	R501 - R2,500 pm	Yes	one	Yes	one	No	No	No	29	No	12	No	1.7
15-19	Yes	No	Yes	R501 - R2,500 pm	Yes	one	Yes	one	Yes	Yes	No	23	No	32	No	1.7
20-24	Yes	No	No	greater than R6,000 pm	Yes	one	Yes	one	No	No	No	34	No	58	No	1.7
20-24	Yes	No	Yes	greater than R6,000 pm	No	one	No	one	Yes	No	No	28	No	23	No	1.9
15-19	No	No	Yes	R501 - R2,500 pm	No	one	Yes	one	No	No	No	31	No	10	No	1.8
15-19	No	No	Yes	greater than R6,000 pm	Yes	one	Yes	one	No	Yes	No	27	No	20	No	1.8
15-19	No	No	Yes	R501 - R2,500 pm	Yes	one	No	one	No	No	No	22	No	39	No	2.1
20-24	No	No	Yes	R501 - R2,500 pm	Yes	one	Yes	one	No	Yes	No	23	No	43	No	1.5
15-19	No	No	Yes	R501 - R2,500 pm	No	one	Yes	one	Yes	Yes	No	20	No	23	No	1.4
15-19	No	No	Yes	greater than R6,000 pm	Yes	one	No	one	No	Yes	No	21	No	39	No	1.5
20-24	Yes	No	Yes	R2,501 - R6,000 pm	Yes	one	Yes	one	No	No	No	29	No	9	No	1.8
15-19	No	No	Yes	R501 - R2,500 pm	No	one	No	one	No	Yes	No	23	No	33	No	1.8
15-19	Yes	No	Yes	R501 - R2,500 pm	No	one	Yes	one	No	No	No	25	No	17	No	1.8
15-19	No	No	Yes	R501 - R2,500 pm	No	one	No	one	No	No	No	19	No	13	No	1.5
20-24	Yes	No	No	R501 - R2,500 pm	Yes	one	Yes	one	No	No	No	28	No	9	No	1.3

20-24	Yes	No	Yes	R501 - R2,500 pm	No	one	Yes	none	.	.	.	.	.	19	No	1.3
15-19	No	No	Yes	R501 - R2,500 pm	No	one	Yes	one	Yes	Yes	No	19	No	17	No	1.3
20-24	Yes	No	Yes	R2,501 - R6,000 pm	No	one	No	one	No	Yes	No	23	No	37	No	1.3
20-24	Yes	No	Yes	R501 - R2,500 pm	Yes	one	Yes	one	No	Yes	No	27	No	37	No	1.4
15-19	No	Yes	Yes	R2,501 - R6,000 pm	No	one	Yes	none	.	.	No	17	.	29	No	1.6
20-24	Yes	Yes	Yes	R501 - R2,500 pm	Yes	one	Yes	none	.	No	No	23	.	32	No	1.3
20-24	Yes	Yes	Yes	R501 - R2,500 pm	No	one	No	one	No	Yes	No	25	Yes	28	No	1.3
15-19	No	Yes	Yes	R2,501 - R6,000 pm	Yes	one	Yes	one	Yes	Yes	No	21	No	19	No	1.5
15-19	No	Yes	Yes	R2,501 - R6,000 pm	Yes	one	Yes	one	No	.	No	21	No	28	No	1.5
20-24	Yes	Yes	Yes	R2,501 - R6,000 pm	No	one	Yes	one	No	Yes	No	25	No	56	No	1.5
15-19	No	Yes	Yes	R501 - R2,500 pm	Yes	one	Yes	one	No	Yes	No	27	No	32	No	1.3
15-19	No	Yes	Yes	R501 - R2,500 pm	Yes	one	Yes	none	.	.	No	19	.	53	No	1.3
15-19	Yes	Yes	Yes	R2,501 - R6,000 pm	No	one	No	one	No	No	No	26	No	52	No	0.9
20-24	Yes	Yes	Yes	R2,501 - R6,000 pm	No	one	No	one	No	Yes	No	29	No	60	No	1.9
20-24	Yes	Yes	Yes	R501 - R2,500 pm	Yes	one	No	one	No	Yes	No	25	No	25	No	1.3
20-24	Yes	Yes	Yes	R2,501 - R6,000 pm	Yes	one	No	one	No	Yes	No	23	No	23	No	1.3
20-24	No	Yes	Yes	R2,501 - R6,000 pm	Yes	one	Yes	one	No	No	Yes	26	No	12	No	1.3
20-24	Yes	Yes	Yes	R501 - R2,500 pm	Yes	one	Yes	one	Yes	No	No	29	No	27	No	1.3
20-24	No	Yes	Yes	R0 - R500 pm	Yes	one	Yes	one	No	Yes	No	28	No	10	No	1.3
15-19	No	Yes	No	R501 - R2,500 pm	No	one	No	one	No	No	No	23	No	31	No	1.3
20-24	No	Yes	Yes	R501 - R2,500 pm	Yes	one	Yes	one	No	No	No	31	No	24	No	1.3
15-19	Yes	Yes	Yes	R0 - R500 pm	Yes	one	No	one	Yes	Yes	No	25	No	34	No	1.3
15-19	No	Yes	Yes	R2,501 - R6,000 pm	No	one	No	one	No	Yes	No	25	No	23	Yes	0.7
20-24	No	Yes	Yes	R501 - R2,500 pm	No	one	Yes	none	.	Yes	No	30	.	25	No	1.3
15-19	Yes	Yes	Yes	greater than R6,000 pm	Yes	one	Yes	one	No	Yes	No	23	No	43	No	1.3
20-24	Yes	Yes	Yes	R501 - R2,500 pm	No	one	Yes	one	Yes	Yes	No	25	No	10	No	1.3
20-24	Yes	Yes	Yes	R0 - R500 pm	Yes	one	Yes	one	No	Yes	No	28	No	19	No	1.7
20-24	Yes	Yes	Yes	R501 - R2,500 pm	Yes	one	Yes	one	No	Yes	No	35	Yes	27	No	1.9
20-24	Yes	Yes	Yes	R501 - R2,500 pm	No	one	No	none	.	.	.	.	.	10	No	1.3
15-19	Yes	Yes	Yes	R501 - R2,500 pm	Yes	one	Yes	2 or more	No	Yes	No	32	No	9	No	1.3
20-24	No	Yes	Yes	R501 - R2,500 pm	Yes	one	Yes	one	Yes	Yes	No	23	No	27	No	1.3
20-24	Yes	Yes	Yes	R2,501 - R6,000 pm	No	one	No	one	Yes	Yes	No	26	No	60	No	1.4
15-19	No	Yes	Yes	R2,501 - R6,000 pm	No	one	Yes	one	No	Yes	No	25	No	23	No	1.3
15-19	No	Yes	No	R2,501 - R6,000 pm	Yes	one	Yes	one	No	No	No	23	No	32	No	1.3
20-24	Yes	Yes	Yes	greater than R6,000 pm	Yes	one	Yes	one	No	Yes	No	27	No	31	No	1.4
20-24	Yes	Yes	No	R2,501 - R6,000 pm	Yes	one	Yes	one	No	No	No	31	No	11	No	1.2
20-24	Yes	Yes	Yes	R2,501 - R6,000 pm	No	one	Yes	one	Yes	Yes	No	27	No	25	No	1.4
20-24	No	Yes	No	R2,501 - R6,000 pm	Yes	one	No	one	No	Yes	No	22	No	10	No	1.1
15-19	No	Yes	Yes	R501 - R2,500 pm	No	one	Yes	one	Yes	Yes	No	19	No	24	No	1.3
15-19	No	Yes	Yes	R2,501 - R6,000 pm	Yes	one	Yes	none	.	Yes	No	18	.	30	No	1.3
20-24	No	Yes	Yes	R2,501 - R6,000 pm	Yes	one	Yes	one	No	Yes	No	27	No	25	No	1.3
15-19	Yes	Yes	Yes	R2,501 - R6,000 pm	No	one	Yes	one	No	No	No	23	No	37	No	1.3
15-19	Yes	Yes	Yes	R0 - R500 pm	Yes	one	No	one	No	Yes	No	24	No	18	No	1.4
15-19	No	Yes	Yes	R2,501 - R6,000 pm	No	one	No	one	No	No	No	23	No	47	No	1.4

20-24	Yes	Yes	Yes	greater than R6,000 pm	Yes	one	Yes	one	No	Yes	No	25	Yes	60	Yes	0.9
20-24	No	Yes	Yes	R2,501 - R6,000 pm	No	one	Yes	one	No	Yes	No	29	No	12	No	1.6
20-24	Yes	No	Yes	R501 - R2,500 pm	Yes	one	Yes	one	No	Yes	No	28	No	27	No	1.9
15-19	No	No	Yes	R501 - R2,500 pm	Yes	one	No	one	No	Yes	No	21	No	24	No	1.9
20-24	No	No	Yes	R2,501 - R6,000 pm	Yes	one	Yes	one	No	No	No	28	No	28	No	1.9
15-19	Yes	No	Yes	R501 - R2,500 pm	Yes	one	Yes	one	No	No	No	20	No	24	No	1.5
15-19	Yes	No	Yes	R501 - R2,500 pm	No	one	Yes	one	No	No	No	22	No	41	No	1.2
15-19	No	No	Yes	R2,501 - R6,000 pm	Yes	one	No	one	No	Yes	No	21	No	13	No	1.2
20-24	No	Yes	Yes	R501 - R2,500 pm	Yes	one	Yes	one	No	No	No	27	No	46	No	1.4
20-24	No	Yes	No	R2,501 - R6,000 pm	Yes	one	Yes	one	No	Yes	No	35	No	33	No	1.4
20-24	No	Yes	Yes	R501 - R2,500 pm	Yes	one	Yes	one	No	Yes	No	24	No	15	No	1.7
15-19	Yes	Yes	Yes	R2,501 - R6,000 pm	Yes	one	Yes	one	No	Yes	No	22	No	14	No	1.4
20-24	No	Yes	Yes	R2,501 - R6,000 pm	Yes	one	Yes	one	No	No	No	30	Yes	11	No	1.1
15-19	No	Yes	Yes	R2,501 - R6,000 pm	No	one	No	one	No	No	No	23	Yes	11	Yes	0.7
15-19	Yes	Yes	Yes	greater than R6,000 pm	Yes	one	No	one	No	Yes	No	22	Yes	21	No	1.4
15-19	Yes	Yes	Yes	R501 - R2,500 pm	Yes	one	Yes	one	No	No	No	25	Yes	11	No	1.5
20-24	Yes	Yes	Yes	R501 - R2,500 pm	Yes	one	Yes	one	Yes	No	No	26	No	10	No	1.3
20-24	Yes	Yes	Yes	R2,501 - R6,000 pm	Yes	one	Yes	one	No	Yes	No	33	No	21	No	1.4
15-19	Yes	Yes	Yes	R501 - R2,500 pm	No	one	No	2 or more	No	Yes	No	29	No	11	No	1.4
20-24	No	Yes	Yes	R501 - R2,500 pm	Yes	one	Yes	none	.	No	No	28	.	12	No	1.4
20-24	No	Yes	Yes	R2,501 - R6,000 pm	No	one	Yes	one	No	Yes	No	28	No	46	No	1.4
20-24	No	No	Yes	R501 - R2,500 pm	Yes	one	Yes	one	No	Yes	No	22	No	24	Yes	1.2
20-24	Yes	No	Yes	R2,501 - R6,000 pm	Yes	one	Yes	none	.	Yes	No	24	.	34	No	1.2
20-24	Yes	Yes	Yes	R501 - R2,500 pm	Yes	one	Yes	one	No	Yes	No	28	No	58	No	1.4
15-19	No	Yes	Yes	greater than R6,000 pm	No	one	No	one	No	Yes	No	21	No	31	No	1.4
15-19	No	Yes	Yes	R501 - R2,500 pm	No	one	No	none	.	Yes	No	19	.	21	No	1.4
20-24	Yes	Yes	Yes	R501 - R2,500 pm	No	one	No	one	Yes	Yes	No	28	No	12	No	1.4
15-19	No	Yes	Yes	R2,501 - R6,000 pm	No	one	No	one	No	Yes	No	20	No	42	No	1.4
20-24	Yes	Yes	Yes	R501 - R2,500 pm	Yes	one	Yes	one	No	Yes	No	29	No	22	No	1.2
20-24	No	Yes	Yes	R501 - R2,500 pm	Yes	one	Yes	one	No	Yes	No	25	No	22	No	1.2
20-24	Yes	Yes	Yes	R2,501 - R6,000 pm	Yes	one	Yes	one	No	Yes	No	21	Yes	22	No	1.2
20-24	Yes	Yes	Yes	R2,501 - R6,000 pm	Yes	one	Yes	one	No	No	No	28	No	24	No	1.2
20-24	Yes	Yes	Yes	greater than R6,000 pm	Yes	one	No	one	Yes	No	Yes	26	No	34	No	1.2
20-24	No	Yes	Yes	R2,501 - R6,000 pm	Yes	one	Yes	one	Yes	No	No	35	No	12	Yes	0.6
15-19	No	Yes	Yes	R501 - R2,500 pm	No	one	No	one	No	Yes	No	20	No	38	No	1.2
15-19	No	No	Yes	R501 - R2,500 pm	No	one	No	one	No	No	No	20	No	16	No	1.6
20-24	Yes	No	Yes	R2,501 - R6,000 pm	Yes	one	No	one	Yes	Yes	Yes	24	Yes	25	No	1.2
20-24	Yes	No	Yes	R501 - R2,500 pm	No	one	No	one	No	Yes	No	30	No	13	No	1.8
20-24	Yes	No	Yes	R0 - R500 pm	No	one	Yes	none	.	No	No	24	.	50	No	1.7
15-19	No	No	Yes	R501 - R2,500 pm	Yes	one	Yes	one	Yes	No	No	21	No	34	No	1.7
20-24	Yes	No	Yes	R2,501 - R6,000 pm	Yes	one	Yes	none	.	Yes	No	23	.	13	No	1.7

20-24	Yes	No	Yes	greater than R6,000 pm	Yes	one	No	one	Yes	No	No	24	No	49	No	1.3
20-24	No	No	Yes	R501 - R2,500 pm	No	one	No	one	No	No	No	35	No	60	No	1.4
15-19	No	No	Yes	R501 - R2,500 pm	Yes	one	Yes	one	No	No	No	24	No	27	No	1.7
20-24	No	No	Yes	R501 - R2,500 pm	Yes	one	Yes	one	No	No	Yes	30	No	42	Yes	0.8
15-19	No	No	Yes	R501 - R2,500 pm	No	one	Yes	one	Yes	Yes	No	23	No	25	No	1.7
15-19	No	No	Yes	R501 - R2,500 pm	No	one	Yes	one	No	No	No	23	No	25	No	1.7
20-24	Yes	No	Yes	R2,501 - R6,000 pm	Yes	one	Yes	one	No	Yes	No	29	No	56	No	1.7
20-24	No	Yes	Yes	R2,501 - R6,000 pm	No	one	Yes	one	No	Yes	No	26	Yes	12	No	1.2
20-24	Yes	Yes	Yes	R0 - R500 pm	No	one	No	one	No	Yes	No	25	No	10	No	1.3
20-24	Yes	Yes	Yes	R501 - R2,500 pm	Yes	one	Yes	none	.	Yes	No	20	.	29	No	1.3
20-24	Yes	Yes	No	R2,501 - R6,000 pm	No	one	Yes	one	No	Yes	No	25	No	18	No	1.4
20-24	Yes	Yes	Yes	R501 - R2,500 pm	Yes	one	Yes	one	No	Yes	No	24	No	22	No	1.4
15-19	No	Yes	Yes	R501 - R2,500 pm	Yes	one	No	one	No	Yes	No	22	No	21	No	1.4
15-19	No	Yes	Yes	R501 - R2,500 pm	Yes	one	Yes	one	No	Yes	No	23	No	15	No	1.1
20-24	Yes	Yes	Yes	R501 - R2,500 pm	Yes	one	Yes	one	No	No	No	23	No	17	No	1.1
20-24	Yes	Yes	Yes	R501 - R2,500 pm	Yes	one	Yes	one	No	No	No	25	No	32	No	1.2
20-24	Yes	Yes	Yes	R501 - R2,500 pm	Yes	one	Yes	one	Yes	No	No	25	No	35	No	1.3
20-24	Yes	Yes	No	R0 - R500 pm	Yes	one	Yes	one	No	No	No	27	No	12	No	1.3
20-24	Yes	Yes	Yes	R2,501 - R6,000 pm	Yes	one	Yes	one	No	Yes	No	23	No	25	No	1.3
20-24	Yes	Yes	Yes	R2,501 - R6,000 pm	Yes	one	Yes	one	No	No	No	26	No	45	No	1.4
15-19	No	Yes	Yes	R2,501 - R6,000 pm	Yes	one	Yes	one	No	No	No	24	No	21	No	1.4
20-24	Yes	Yes	Yes	R2,501 - R6,000 pm	Yes	one	Yes	one	No	Yes	No	33	No	23	No	1.4
15-19	No	Yes	Yes	R501 - R2,500 pm	No	one	Yes	one	No	Yes	No	21	No	41	No	1.4
20-24	Yes	Yes	Yes	R2,501 - R6,000 pm	Yes	one	No	none	.	Yes	No	20	.	24	No	1.3
20-24	No	Yes	No	R501 - R2,500 pm	No	one	No	one	No	No	No	32	No	12	No	1.3
20-24	No	Yes	Yes	R2,501 - R6,000 pm	Yes	one	Yes	one	No	Yes	No	24	Yes	22	No	1.9
15-19	No	Yes	Yes	R501 - R2,500 pm	No	one	Yes	none	.	Yes	No	21	.	32	No	1.3
15-19	No	Yes	Yes	R501 - R2,500 pm	Yes	one	Yes	one	No	Yes	No	23	No	21	No	1.3
20-24	Yes	Yes	Yes	R501 - R2,500 pm	No	one	Yes	one	No	Yes	No	25	No	23	No	1
20-24	Yes	Yes	Yes	R2,501 - R6,000 pm	Yes	one	Yes	2 or more	.	No	No	28	No	23	No	1.3
15-19	No	Yes	Yes	R2,501 - R6,000 pm	Yes	one	Yes	one	No	Yes	No	24	No	21	No	1.1
15-19	No	Yes	No	R2,501 - R6,000 pm	Yes	one	Yes	one	No	No	No	24	No	24	No	1.4
20-24	No	Yes	No	R2,501 - R6,000 pm	Yes	one	Yes	one	No	Yes	No	32	No	10	No	1.4
20-24	No	Yes	No	greater than R6,000 pm	Yes	one	Yes	one	No	No	No	47	No	10	No	1.4
20-24	Yes	Yes	Yes	greater than R6,000 pm	Yes	one	Yes	none	.	No	No	19	.	10	No	1.1
15-19	No	Yes	Yes	R501 - R2,500 pm	Yes	one	Yes	none	.	Yes	No	24	.	17	No	1.1
20-24	Yes	Yes	No	R501 - R2,500 pm	Yes	one	Yes	one	Yes	Yes	No	28	No	10	No	1.3
20-24	No	Yes	Yes	R2,501 - R6,000 pm	Yes	one	Yes	none	.	No	No	25	.	53	No	1.2
20-24	No	Yes	Yes	R501 - R2,500 pm	Yes	one	Yes	one	No	Yes	No	27	No	29	Yes	0.5
20-24	Yes	Yes	Yes	R2,501 - R6,000 pm	No	one	Yes	one	No	Yes	No	31	No	10	No	1.1
20-24	Yes	Yes	Yes	R501 - R2,500 pm	No	one	No	one	No	Yes	No	29	No	18	No	1.3
20-24	Yes	Yes	Yes	R2,501 - R6,000 pm	Yes	one	Yes	one	Yes	Yes	No	24	No	10	No	1.2

15-19	No	Yes	Yes	greater than R6,000 pm	Yes	one	Yes	one	Yes	No	No	19	No	13	No	1.1
15-19	No	Yes	Yes	R2,501 - R6,000 pm	No	one	No	one	No	Yes	No	22	No	22	No	1.9
20-24	Yes	Yes	Yes	R501 - R2,500 pm	Yes	one	No	one	No	Yes	No	27	No	45	Yes	0.7
20-24	Yes	Yes	Yes	R2,501 - R6,000 pm	Yes	one	Yes	one	No	No	No	28	No	24	Yes	0.8
20-24	Yes	Yes	Yes	R2,501 - R6,000 pm	Yes	one	Yes	one	No	Yes	No	29	No	12	No	1.5
20-24	Yes	Yes	Yes	greater than R6,000 pm	Yes	one	Yes	one	Yes	No	No	30	No	11	No	1.4
15-19	No	Yes	Yes	greater than R6,000 pm	Yes	one	No	none	.	Yes	No	23	.	21	No	1.5
20-24	Yes	Yes	Yes	greater than R6,000 pm	Yes	one	Yes	one	Yes	Yes	No	30	No	50	No	1.9
15-19	No	Yes	Yes	R501 - R2,500 pm	Yes	one	No	one	Yes	Yes	No	23	No	22	No	1.9
20-24	No	Yes	No	R501 - R2,500 pm	No	one	Yes	one	No	No	No	29	No	13	No	1.5
20-24	No	Yes	Yes	R501 - R2,500 pm	Yes	one	Yes	one	No	Yes	No	50	No	13	No	1.5
20-24	Yes	Yes	Yes	greater than R6,000 pm	No	one	Yes	one	No	No	No	27	No	24	No	1.7
15-19	No	Yes	Yes	R2,501 - R6,000 pm	Yes	one	Yes	2 or more	No	No	No	26	No	29	No	1.5
20-24	No	Yes	No	R2,501 - R6,000 pm	Yes	one	Yes	one	No	No	No	27	No	43	No	1.5
15-19	No	Yes	Yes	R2,501 - R6,000 pm	Yes	one	Yes	one	No	Yes	No	23	No	10	No	1.5
20-24	No	Yes	Yes	R501 - R2,500 pm	Yes	one	Yes	none	.	.	.	.	.	32	No	1.5
15-19	Yes	Yes	Yes	R501 - R2,500 pm	No	one	Yes	one	No	No	No	32	No	32	No	1.5
20-24	Yes	Yes	No	R501 - R2,500 pm	Yes	one	Yes	one	No	Yes	No	27	No	19	No	1.5
20-24	Yes	Yes	Yes	R0 - R500 pm	Yes	one	Yes	one	No	Yes	No	34	No	45	No	1.8
15-19	No	Yes	Yes	R501 - R2,500 pm	Yes	one	Yes	2 or more	No	No	No	23	Yes	14	No	1.8
15-19	No	Yes	Yes	R2,501 - R6,000 pm	No	one	Yes	2 or more	No	Yes	No	21	Yes	42	No	1.8
20-24	Yes	No	Yes	R2,501 - R6,000 pm	Yes	one	Yes	one	Yes	No	No	24	No	34	No	1.4
20-24	Yes	No	Yes	R2,501 - R6,000 pm	No	one	No	one	No	Yes	No	23	No	24	No	1.3
20-24	No	No	Yes	R501 - R2,500 pm	No	one	Yes	one	No	No	No	23	No	46	No	1.1
20-24	Yes	No	Yes	R501 - R2,500 pm	No	one	Yes	none	.	Yes	No	24	.	24	No	1.1
15-19	Yes	No	Yes	R501 - R2,500 pm	No	one	Yes	one	No	.	No	21	No	11	No	1.1
15-19	Yes	No	Yes	R501 - R2,500 pm	No	one	No	one	No	No	No	24	No	43	No	1.7
15-19	No	No	Yes	R501 - R2,500 pm	No	one	Yes	one	Yes	Yes	No	23	No	43	No	1.7
20-24	Yes	No	Yes	R501 - R2,500 pm	No	one	No	one	No	No	No	27	Yes	48	No	1.8
20-24	Yes	No	Yes	R501 - R2,500 pm	Yes	one	Yes	none	.	Yes	No	27	.	12	No	1.8
20-24	Yes	No	Yes	R2,501 - R6,000 pm	Yes	one	Yes	one	No	Yes	No	27	No	16	No	1.1
15-19	No	No	No	R2,501 - R6,000 pm	Yes	one	Yes	one	No	No	No	21	No	15	No	1.1
20-24	Yes	No	Yes	R2,501 - R6,000 pm	Yes	one	Yes	one	No	Yes	No	25	No	31	No	1.1
20-24	Yes	No	Yes	R2,501 - R6,000 pm	Yes	one	Yes	none	.	No	No	30	.	12	No	1.2
15-19	No	No	Yes	R2,501 - R6,000 pm	No	one	No	one	Yes	Yes	No	19	No	21	No	1.2
20-24	No	No	Yes	greater than R6,000 pm	Yes	one	Yes	none	.	No	No	23	.	12	No	1.3
15-19	No	No	Yes	R501 - R2,500 pm	No	one	Yes	one	No	Yes	No	21	No	21	Yes	0.6
20-24	No	No	Yes	greater than R6,000 pm	Yes	one	No	none	.	Yes	No	24	.	22	No	1.4
15-19	No	No	Yes	R501 - R2,500 pm	Yes	one	Yes	one	No	No	No	21	No	10	No	1.2

20-24	No	No	Yes	R501 - R2,500 pm	Yes	one	Yes	2 or more	No	Yes	No	26	No	32	No	1.2
20-24	Yes	No	Yes	R501 - R2,500 pm	Yes	one	Yes	one	No	Yes	No	28	Yes	17	No	1.5
20-24	Yes	No	Yes	R2,501 - R6,000 pm	Yes	one	Yes	one	No	No	No	25	No	34	No	1.6
20-24	Yes	No	Yes	R2,501 - R6,000 pm	Yes	one	Yes	one	No	Yes	No	28	No	16	No	1.7
15-19	Yes	No	Yes	R501 - R2,500 pm	Yes	one	Yes	one	No	Yes	No	22	No	43	No	2
20-24	Yes	No	Yes	R501 - R2,500 pm	Yes	one	Yes	none	.	No	No	23	.	17	No	1.6
15-19	No	No	Yes	R501 - R2,500 pm	Yes	one	Yes	one	No	Yes	No	22	No	45	No	1.3
15-19	Yes	No	No	greater than R6,000 pm	Yes	one	No	none	.	Yes	No	25	.	20	No	1.7
20-24	Yes	No	Yes	R2,501 - R6,000 pm	No	one	No	one	Yes	Yes	No	24	No	44	Yes	0.8
15-19	No	No	Yes	R2,501 - R6,000 pm	Yes	one	Yes	one	No	Yes	No	22	No	37	No	1
15-19	No	No	No	R2,501 - R6,000 pm	No	one	No	none	.	Yes	No	20	.	39	No	1.6
20-24	Yes	No	Yes	greater than R6,000 pm	Yes	one	Yes	one	No	No	No	32	No	41	No	1.6
15-19	No	No	Yes	R501 - R2,500 pm	No	one	No	one	Yes	Yes	No	19	No	53	No	1.6
15-19	Yes	No	Yes	R501 - R2,500 pm	No	one	No	one	No	No	No	22	Yes	24	Yes	0.7
15-19	No	No	Yes	R501 - R2,500 pm	No	one	No	one	No	Yes	No	22	Yes	27	No	1.5
20-24	Yes	No	Yes	R501 - R2,500 pm	Yes	one	Yes	one	No	Yes	No	30	No	60	No	1.2
15-19	No	No	Yes	R501 - R2,500 pm	No	one	No	one	No	Yes	No	19	No	52	No	1.2
15-19	Yes	No	Yes	R501 - R2,500 pm	No	one	Yes	one	No	Yes	No	24	No	11	No	1.1
20-24	No	No	Yes	R2,501 - R6,000 pm	Yes	one	Yes	one	No	Yes	No	26	No	12	No	1.2
15-19	No	No	No	R501 - R2,500 pm	Yes	one	No	one	No	Yes	No	21	No	33	No	1.4
15-19	Yes	No	Yes	R2,501 - R6,000 pm	Yes	one	No	one	Yes	Yes	No	22	No	33	No	1.2
20-24	Yes	No	Yes	R2,501 - R6,000 pm	Yes	one	Yes	one	No	Yes	No	26	No	60	No	1.2
20-24	No	No	Yes	R2,501 - R6,000 pm	Yes	one	Yes	one	No	Yes	No	25	No	12	No	1.2
15-19	No	No	Yes	R501 - R2,500 pm	No	one	Yes	none	.	.	.	.	.	34	No	1.2
20-24	Yes	No	Yes	R2,501 - R6,000 pm	Yes	one	No	none	.	Yes	No	25	.	24	No	1.2
20-24	No	No	Yes	R2,501 - R6,000 pm	Yes	one	Yes	one	No	No	No	24	Yes	55	No	1.1
20-24	Yes	No	Yes	greater than R6,000 pm	Yes	one	Yes	one	No	Yes	No	29	Yes	41	No	1.2
20-24	No	No	Yes	R501 - R2,500 pm	No	one	Yes	none	.	Yes	No	25	.	14	No	1.3
20-24	Yes	Yes	Yes	greater than R6,000 pm	Yes	one	Yes	one	No	No	No	25	No	28	No	1
20-24	No	Yes	Yes	R2,501 - R6,000 pm	Yes	one	Yes	one	No	No	No	23	No	15	No	1.3
20-24	Yes	Yes	Yes	greater than R6,000 pm	Yes	one	Yes	one	No	Yes	No	30	Yes	14	No	1.3
15-19	No	Yes	Yes	R2,501 - R6,000 pm	No	one	Yes	one	No	Yes	No	32	No	25	No	1.3
15-19	No	Yes	Yes	R501 - R2,500 pm	Yes	one	No	one	No	No	No	19	No	25	No	1
20-24	Yes	Yes	No	R501 - R2,500 pm	Yes	one	Yes	one	No	No	No	25	No	10	No	1.1
15-19	No	Yes	Yes	R501 - R2,500 pm	No	one	No	one	No	Yes	No	19	Yes	40	No	1.4
15-19	No	Yes	Yes	R501 - R2,500 pm	No	one	No	one	No	Yes	No	23	Yes	40	No	1.4
15-19	No	Yes	Yes	R501 - R2,500 pm	No	one	No	one	Yes	Yes	No	19	No	20	No	1.3
15-19	No	Yes	Yes	R2,501 - R6,000 pm	Yes	one	No	one	Yes	Yes	No	21	No	38	No	1.4
20-24	Yes	Yes	No	R0 - R500 pm	No	one	Yes	one	No	No	No	27	No	64	No	1.4
15-19	Yes	Yes	Yes	R2,501 - R6,000 pm	Yes	one	No	one	No	Yes	No	21	No	24	No	1.1
15-19	No	Yes	Yes	R501 - R2,500 pm	No	one	No	one	No	Yes	No	19	Yes	17	No	1.1

15-19	Yes	Yes	Yes	R2,501 - R6,000 pm	No	one	Yes	one	Yes	No	No	21	Yes	20	No	1.1
20-24	Yes	Yes	Yes	R2,501 - R6,000 pm	Yes	one	Yes	none	.	.	.	.	.	18	No	1.1
20-24	No	Yes	Yes	R501 - R2,500 pm	Yes	one	Yes	one	No	No	No	25	No	12	No	1.3
15-19	Yes	Yes	Yes	R2,501 - R6,000 pm	No	one	No	none	.	.	.	.	.	22	No	1.2
15-19	No	Yes	Yes	R501 - R2,500 pm	Yes	one	Yes	one	Yes	Yes	No	20	No	22	No	1.3
15-19	Yes	Yes	Yes	R2,501 - R6,000 pm	Yes	one	Yes	one	Yes	Yes	No	23	No	21	No	1.2
15-19	No	Yes	Yes	R2,501 - R6,000 pm	No	one	No	one	No	No	No	26	No	26	No	1.1
20-24	Yes	Yes	Yes	R2,501 - R6,000 pm	Yes	one	Yes	one	No	No	No	29	No	30	No	1.3
15-19	No	Yes	Yes	R2,501 - R6,000 pm	No	one	Yes	none	.	.	No	21	.	9	No	1.1
15-19	Yes	Yes	Yes	R2,501 - R6,000 pm	Yes	one	No	one	No	Yes	No	21	Yes	42	No	1.1
15-19	No	Yes	Yes	R501 - R2,500 pm	No	one	Yes	one	No	Yes	No	21	No	9	No	1.1
20-24	Yes	Yes	Yes	R501 - R2,500 pm	No	one	Yes	one	No	No	No	28	No	21	No	1.3
20-24	No	Yes	Yes	R501 - R2,500 pm	No	one	No	none	.	Yes	No	23	.	15	No	1.4
15-19	No	Yes	Yes	R2,501 - R6,000 pm	Yes	one	Yes	one	No	Yes	No	23	No	32	No	1.3
20-24	No	Yes	Yes	R2,501 - R6,000 pm	Yes	one	No	one	No	Yes	No	24	Yes	58	No	1.6
15-19	No	Yes	Yes	R501 - R2,500 pm	No	one	Yes	one	No	No	No	20	No	27	No	1.1
15-19	Yes	Yes	Yes	greater than R6,000 pm	Yes	one	Yes	one	No	Yes	No	24	No	19	No	1.4
20-24	Yes	Yes	Yes	R0 - R500 pm	No	one	No	one	No	Yes	No	30	No	21	No	1.7
15-19	Yes	Yes	Yes	greater than R6,000 pm	No	one	Yes	one	No	Yes	No	24	No	49	No	1.3
15-19	No	Yes	Yes	greater than R6,000 pm	No	one	No	none	.	Yes	No	17	.	33	No	1.5
20-24	Yes	No	Yes	R2,501 - R6,000 pm	Yes	one	Yes	none	.	Yes	No	22	.	20	No	1.4
20-24	Yes	No	No	R2,501 - R6,000 pm	Yes	one	Yes	one	No	Yes	No	25	No	22	No	1.4
20-24	Yes	No	No	R2,501 - R6,000 pm	No	one	Yes	one	No	Yes	No	34	No	31	No	1.9
20-24	Yes	No	Yes	R501 - R2,500 pm	Yes	one	Yes	one	No	Yes	No	25	No	11	No	1.5
20-24	Yes	No	Yes	R2,501 - R6,000 pm	Yes	one	Yes	one	Yes	Yes	No	24	No	20	No	1.5
15-19	Yes	Yes	No	R2,501 - R6,000 pm	No	one	Yes	one	No	No	Yes	24	Yes	23	No	1.8
15-19	Yes	Yes	Yes	R501 - R2,500 pm	Yes	one	Yes	one	Yes	No	No	24	Yes	23	No	1.8
20-24	Yes	Yes	Yes	R501 - R2,500 pm	Yes	one	Yes	one	No	No	No	26	Yes	13	No	1.9
20-24	Yes	Yes	Yes	greater than R6,000 pm	Yes	one	No	one	No	No	No	27	No	22	No	1.2
15-19	No	Yes	Yes	R501 - R2,500 pm	Yes	one	Yes	one	No	Yes	No	28	Yes	22	No	1.8
20-24	Yes	Yes	Yes	R501 - R2,500 pm	Yes	one	Yes	one	No	Yes	No	27	No	13	No	1.3
20-24	Yes	Yes	Yes	R2,501 - R6,000 pm	No	one	No	2 or more	No	Yes	No	28	No	31	No	1.1
20-24	Yes	Yes	Yes	R0 - R500 pm	No	one	No	one	Yes	Yes	No	23	No	22	No	1.2
15-19	No	Yes	Yes	R501 - R2,500 pm	Yes	one	Yes	one	No	No	No	24	No	45	No	1.2
20-24	Yes	Yes	Yes	greater than R6,000 pm	Yes	one	Yes	one	Yes	Yes	No	34	Yes	30	No	1.3
15-19	No	Yes	Yes	R2,501 - R6,000 pm	No	one	No	one	No	Yes	No	18	Yes	36	Yes	0.6
20-24	Yes	Yes	No	R501 - R2,500 pm	Yes	one	No	one	No	Yes	No	28	No	26	No	1
15-19	Yes	Yes	Yes	greater than R6,000 pm	Yes	one	Yes	one	No	Yes	No	23	No	23	No	1
20-24	Yes	Yes	Yes	R501 - R2,500 pm	Yes	one	No	one	No	Yes	No	25	No	36	No	1.2
15-19	Yes	Yes	Yes	R501 - R2,500 pm	Yes	one	Yes	one	No	Yes	No	23	Yes	17	No	1.2

15-19	No	Yes	Yes	greater than R6,000 pm	Yes	one	No	one	Yes	Yes	No	25	No	23	No	1.3
20-24	Yes	Yes	Yes	R2,501 - R6,000 pm	No	one	Yes	one	No	Yes	No	24	No	17	No	1.6
20-24	Yes	Yes	Yes	greater than R6,000 pm	Yes	one	No	one	No	Yes	No	29	No	18	No	1.3
20-24	Yes	Yes	Yes	R2,501 - R6,000 pm	No	one	No	one	No	No	No	32	No	32	No	1.9
15-19	No	Yes	Yes	R501 - R2,500 pm	Yes	one	No	one	Yes	No	No	19	Yes	21	No	1.5
20-24	No	Yes	Yes	R501 - R2,500 pm	Yes	one	Yes	one	No	No	No	27	No	12	No	1.7
20-24	Yes	Yes	Yes	R2,501 - R6,000 pm	Yes	one	Yes	one	No	Yes	No	20	No	28	No	1.2
20-24	Yes	Yes	Yes	greater than R6,000 pm	Yes	one	Yes	one	No	.	No	27	No	32	No	1.6
15-19	No	Yes	Yes	R501 - R2,500 pm	Yes	one	Yes	one	No	No	No	25	No	24	No	1.4
15-19	No	Yes	Yes	R2,501 - R6,000 pm	Yes	one	No	one	No	Yes	No	23	No	24	No	1.4
15-19	No	Yes	Yes	R501 - R2,500 pm	No	one	Yes	one	No	No	No	23	No	52	No	1.4
20-24	Yes	Yes	Yes	R501 - R2,500 pm	Yes	one	No	one	Yes	Yes	No	37	No	21	No	1.7
15-19	Yes	Yes	No	R0 - R500 pm	No	one	Yes	2 or more	No	Yes	No	28	No	18	Yes	0.7
15-19	No	Yes	Yes	R501 - R2,500 pm	No	one	Yes	none	.	Yes	No	21	.	28	No	1.4
20-24	Yes	Yes	Yes	greater than R6,000 pm	No	one	Yes	one	Yes	Yes	No	33	Yes	10	No	1.4
15-19	No	Yes	Yes	R501 - R2,500 pm	No	one	No	one	No	Yes	No	20	No	19	No	1.4
15-19	Yes	Yes	Yes	R2,501 - R6,000 pm	Yes	one	No	one	No	.	No	30	No	10	No	1.4
15-19	No	Yes	Yes	R2,501 - R6,000 pm	Yes	one	No	one	No	Yes	No	27	No	19	No	1.4
15-19	No	Yes	Yes	greater than R6,000 pm	Yes	one	No	one	Yes	Yes	No	19	No	21	No	1.5
20-24	Yes	Yes	Yes	R501 - R2,500 pm	Yes	one	No	one	No	Yes	No	41	No	12	No	1.4
15-19	No	Yes	Yes	R2,501 - R6,000 pm	Yes	one	Yes	one	No	Yes	No	21	No	21	No	1.4
15-19	No	Yes	Yes	greater than R6,000 pm	Yes	one	No	one	Yes	No	No	19	No	32	No	1.5
15-19	No	Yes	Yes	R2,501 - R6,000 pm	No	one	No	one	No	No	No	19	No	32	No	1.5
20-24	Yes	No	Yes	R2,501 - R6,000 pm	Yes	one	Yes	one	No	No	No	28	No	30	No	2
20-24	No	No	Yes	R2,501 - R6,000 pm	Yes	one	Yes	one	No	Yes	No	27	No	20	No	1.7
20-24	Yes	Yes	Yes	greater than R6,000 pm	Yes	one	No	one	No	No	No	30	Yes	28	No	1.4
20-24	Yes	Yes	Yes	R2,501 - R6,000 pm	Yes	one	No	one	Yes	Yes	No	23	No	27	No	1.4
15-19	Yes	Yes	Yes	R501 - R2,500 pm	No	one	Yes	none	.	Yes	No	19	.	48	No	1.4
15-19	No	Yes	Yes	R2,501 - R6,000 pm	Yes	one	No	none	.	Yes	No	17	.	38	No	1.4
20-24	Yes	Yes	Yes	R2,501 - R6,000 pm	No	one	No	one	No	No	No	26	No	38	No	1.8
15-19	No	Yes	Yes	R2,501 - R6,000 pm	No	one	No	one	No	No	No	21	No	24	No	1.1
20-24	No	Yes	Yes	R501 - R2,500 pm	No	one	No	one	No	Yes	No	32	No	37	Yes	0.7
20-24	Yes	Yes	No	R2,501 - R6,000 pm	Yes	one	Yes	one	No	Yes	No	31	No	20	No	1.8
20-24	Yes	Yes	Yes	R501 - R2,500 pm	Yes	one	No	one	No	No	No	27	Yes	18	No	1.3
20-24	Yes	Yes	Yes	R2,501 - R6,000 pm	Yes	one	No	one	No	Yes	No	29	No	10	No	1.3
20-24	Yes	Yes	Yes	R501 - R2,500 pm	Yes	one	Yes	one	Yes	Yes	No	29	No	18	No	1.3
15-19	Yes	Yes	Yes	R2,501 - R6,000 pm	No	one	No	one	Yes	Yes	No	21	No	8	No	1.3
15-19	Yes	Yes	Yes	R0 - R500 pm	Yes	one	No	one	No	Yes	No	27	Yes	11	No	1.4
15-19	No	Yes	Yes	R2,501 - R6,000 pm	Yes	one	Yes	one	No	Yes	No	29	No	21	No	1.9

15-19	No	Yes	Yes	R501 - R2,500 pm	Yes	one	Yes	one	No	Yes	No	20	No	20	No	1.3
20-24	No	Yes	No	R501 - R2,500 pm	Yes	one	Yes	one	No	Yes	No	25	No	12	No	1.6
15-19	No	Yes	Yes	R2,501 - R6,000 pm	No	one	No	one	Yes	Yes	No	20	No	10	No	1.2
15-19	No	Yes	Yes	R501 - R2,500 pm	Yes	one	No	one	Yes	Yes	No	23	No	42	No	1.3
20-24	No	Yes	Yes	R501 - R2,500 pm	Yes	one	Yes	one	No	No	No	26	No	14	No	1.3
20-24	Yes	Yes	Yes	R501 - R2,500 pm	No	one	No	one	No	No	No	22	No	23	No	1.3
15-19	No	Yes	Yes	greater than R6,000 pm	Yes	one	No	one	Yes	No	No	19	No	41	No	1.7
15-19	No	Yes	Yes	R2,501 - R6,000 pm	Yes	one	Yes	one	Yes	Yes	No	21	No	53	No	1
15-19	No	Yes	Yes	R501 - R2,500 pm	Yes	one	Yes	one	Yes	Yes	No	23	Yes	39	No	1.4
15-19	No	Yes	Yes	R501 - R2,500 pm	No	one	No	one	Yes	Yes	No	21	No	27	No	1.4
20-24	Yes	Yes	Yes	R2,501 - R6,000 pm	Yes	one	No	one	No	Yes	No	30	No	36	No	1.1
15-19	No	Yes	Yes	R2,501 - R6,000 pm	Yes	one	No	none	.	.	No	20	.	30	No	1
20-24	Yes	Yes	Yes	R2,501 - R6,000 pm	Yes	one	No	one	Yes	Yes	No	25	No	28	No	1.3
20-24	Yes	Yes	No	R501 - R2,500 pm	Yes	one	No	one	No	No	No	28	No	13	No	1.4
15-19	No	Yes	Yes	R501 - R2,500 pm	Yes	one	Yes	one	No	Yes	No	18	No	9	No	1.4
15-19	No	Yes	Yes	R501 - R2,500 pm	Yes	one	Yes	one	No	Yes	No	24	No	19	No	1.4
20-24	Yes	Yes	Yes	R501 - R2,500 pm	Yes	one	Yes	one	No	Yes	No	31	No	21	No	1.4
20-24	Yes	Yes	Yes	R501 - R2,500 pm	No	one	No	one	No	No	No	23	No	28	No	1.1
15-19	No	No	Yes	R501 - R2,500 pm	Yes	one	Yes	one	Yes	Yes	No	26	No	27	No	1.1
20-24	Yes	No	Yes	greater than R6,000 pm	No	one	No	one	No	No	No	25	No	21	No	1.1
20-24	Yes	No	Yes	R501 - R2,500 pm	Yes	one	Yes	one	No	No	No	26	No	15	No	1.6
15-19	Yes	No	Yes	R2,501 - R6,000 pm	No	one	No	one	No	.	No	23	No	53	No	1.6
20-24	Yes	No	Yes	R2,501 - R6,000 pm	Yes	one	Yes	one	Yes	No	No	24	No	15	No	1.6
15-19	No	No	Yes	R2,501 - R6,000 pm	No	one	No	one	No	Yes	No	21	No	41	No	1.6
20-24	Yes	No	Yes	greater than R6,000 pm	Yes	one	No	one	No	Yes	No	30	No	59	No	1.7
20-24	Yes	No	Yes	greater than R6,000 pm	No	one	Yes	one	Yes	No	No	25	No	60	No	1.7
15-19	No	No	Yes	R501 - R2,500 pm	Yes	one	Yes	one	No	Yes	No	22	No	13	No	1.1
20-24	Yes	No	Yes	R2,501 - R6,000 pm	No	one	Yes	one	No	No	No	27	No	28	No	1.2
20-24	No	No	Yes	R2,501 - R6,000 pm	Yes	one	Yes	one	No	No	No	28	No	28	No	1.3
15-19	No	Yes	Yes	R2,501 - R6,000 pm	Yes	one	Yes	one	No	Yes	No	26	Yes	20	No	1.6
20-24	Yes	Yes	Yes	R2,501 - R6,000 pm	Yes	one	Yes	one	Yes	Yes	No	23	No	33	No	1.6
15-19	No	No	Yes	R501 - R2,500 pm	No	one	No	none	.	Yes	No	17	.	20	No	1.2
20-24	No	No	Yes	R501 - R2,500 pm	Yes	one	Yes	one	No	Yes	No	24	No	11	No	1.2
20-24	Yes	No	Yes	R2,501 - R6,000 pm	Yes	one	Yes	one	No	Yes	No	29	No	31	No	1.7
15-19	Yes	No	Yes	R2,501 - R6,000 pm	No	one	No	one	No	Yes	No	25	No	33	No	1.2
20-24	Yes	No	Yes	R2,501 - R6,000 pm	Yes	one	No	one	No	Yes	No	28	No	36	No	1.2
15-19	No	No	Yes	R2,501 - R6,000 pm	No	one	No	one	No	Yes	No	26	No	33	No	1.2
15-19	Yes	No	Yes	R2,501 - R6,000 pm	Yes	one	Yes	one	No	No	No	28	No	47	No	1.2
20-24	No	No	Yes	R2,501 - R6,000 pm	No	one	Yes	one	No	Yes	No	21	No	15	No	1
20-24	Yes	No	Yes	R2,501 - R6,000 pm	No	one	Yes	one	No	Yes	No	34	Yes	45	No	1
20-24	No	No	Yes	greater than R6,000 pm	Yes	one	Yes	one	No	Yes	No	33	No	29	No	1.1

15-19	Yes	No	Yes	R2,501 - R6,000 pm	No	one	Yes	one	No	Yes	No	21	No	41	No	1
15-19	No	No	Yes	greater than R6,000 pm	Yes	one	Yes	none	.	Yes	No	23	.	27	No	1.1
20-24	No	No	Yes	R2,501 - R6,000 pm	Yes	one	No	none	.	No	No	22	.	19	Yes	0.5
15-19	No	No	Yes	greater than R6,000 pm	No	one	Yes	one	No	Yes	No	19	No	17	No	1.1
15-19	Yes	No	Yes	R501 - R2,500 pm	Yes	one	No	one	Yes	Yes	No	24	No	41	No	1.3
20-24	Yes	No	Yes	R2,501 - R6,000 pm	Yes	one	Yes	one	No	Yes	No	28	No	31	No	1.2
15-19	No	No	Yes	R501 - R2,500 pm	No	one	No	one	No	No	No	19	No	53	No	1.4
15-19	No	No	Yes	R2,501 - R6,000 pm	No	one	Yes	one	No	No	No	25	No	39	No	1.3
15-19	No	No	No	R2,501 - R6,000 pm	Yes	one	No	none	.	No	No	21	.	13	No	1.2
20-24	Yes	No	Yes	R2,501 - R6,000 pm	No	one	Yes	none	.	No	No	22	.	17	No	1.3
15-19	No	No	Yes	R501 - R2,500 pm	Yes	one	Yes	one	No	No	No	23	No	18	No	1.7
20-24	Yes	No	Yes	R2,501 - R6,000 pm	No	one	Yes	one	Yes	Yes	No	25	No	17	No	2
20-24	Yes	No	Yes	R2,501 - R6,000 pm	Yes	one	Yes	one	No	Yes	No	25	No	32	No	1.8
20-24	Yes	No	Yes	R2,501 - R6,000 pm	Yes	one	Yes	one	No	No	No	30	No	32	No	1.8
20-24	Yes	No	No	greater than R6,000 pm	No	one	Yes	one	No	Yes	No	42	No	34	No	1.8
20-24	Yes	No	Yes	R2,501 - R6,000 pm	Yes	one	No	one	No	Yes	No	25	No	22	No	1.1
15-19	No	No	Yes	greater than R6,000 pm	No	one	No	one	No	Yes	No	20	Yes	38	No	1.4
20-24	Yes	No	Yes	R2,501 - R6,000 pm	No	one	No	one	No	Yes	No	22	No	30	No	1.4
20-24	Yes	No	Yes	R2,501 - R6,000 pm	Yes	one	No	one	No	Yes	No	25	No	29	No	1.3
15-19	Yes	No	Yes	greater than R6,000 pm	Yes	one	Yes	none	.	No	No	17	.	32	No	1.7
15-19	Yes	No	Yes	R501 - R2,500 pm	No	one	No	one	No	Yes	No	24	No	23	No	1.6
15-19	No	No	Yes	R2,501 - R6,000 pm	No	04-Feb	No	one	No	.	No	20	No	102	Yes	0.8
20-24	Yes	Yes	Yes	R501 - R2,500 pm	Yes	04-Feb	Yes	none	.	Yes	No	26	.	16	No	1.5
20-24	No	No	Yes	R501 - R2,500 pm	No	04-Feb	Yes	none	.	Yes	No	24	.	56	No	1.5
20-24	Yes	Yes	Yes	greater than R6,000 pm	Yes	04-Feb	No	one	Yes	No	No	24	No	29	No	1.3
20-24	Yes	No	Yes	R0 - R500 pm	Yes	04-Feb	Yes	one	No	Yes	No	30	No	47	No	1.9
20-24	Yes	Yes	Yes	.	Yes	04-Feb	Yes	one	No	No	No	35	No	28	No	2
15-19	Yes	Yes	Yes	R2,501 - R6,000 pm	Yes	04-Feb	No	2 or more	No	Yes	No	18	No	70	No	1.6
20-24	Yes	No	Yes	R501 - R2,500 pm	No	04-Feb	Yes	one	No	Yes	No	26	No	32	No	1.9
20-24	Yes	Yes	Yes	R501 - R2,500 pm	Yes	04-Feb	Yes	one	No	Yes	No	25	No	22	No	2
20-24	Yes	Yes	Yes	R501 - R2,500 pm	Yes	04-Feb	Yes	2 or more	No	No	No	24	No	7	No	1.9
15-19	Yes	Yes	Yes	R2,501 - R6,000 pm	Yes	04-Feb	Yes	one	No	Yes	No	21	No	19	No	1.3
15-19	Yes	No	Yes	greater than R6,000 pm	Yes	04-Feb	No	one	No	Yes	No	30	No	80	No	1.8
20-24	No	No	Yes	R501 - R2,500 pm	No	04-Feb	Yes	one	No	No	No	26	No	59	No	1.4
20-24	Yes	No	Yes	R501 - R2,500 pm	No	04-Feb	Yes	one	No	Yes	No	28	No	28	Yes	0.7
20-24	No	Yes	No	.	Yes	04-Feb	Yes	one	No	Yes	No	34	No	13	No	1.9
20-24	Yes	No	Yes	R2,501 - R6,000 pm	Yes	04-Feb	Yes	one	No	No	No	25	Yes	25	No	1.3
15-19	No	Yes	Yes	greater than R6,000 pm	No	04-Feb	Yes	none	.	.	No	20	.	16	No	1.2

20-24	Yes	Yes	Yes	R501 - R2,500 pm	Yes	04-Feb	Yes	one	No	No	No	28	Yes	7	No	1.9
20-24	Yes	No	Yes	R2,501 - R6,000 pm	Yes	04-Feb	No	one	No	Yes	No	26	No	28	No	1.4
15-19	Yes	No	Yes	R501 - R2,500 pm	Yes	04-Feb	No	2 or more	No	Yes	No	24	No	30	Yes	0.7
20-24	No	Yes	Yes	R0 - R500 pm	No	04-Feb	Yes	one	No	No	No	27	No	10	No	1.6
20-24	Yes	Yes	Yes	R501 - R2,500 pm	Yes	04-Feb	Yes	one	Yes	Yes	No	30	No	12	No	1.6
20-24	Yes	Yes	Yes	R501 - R2,500 pm	Yes	04-Feb	Yes	one	No	No	No	27	Yes	45	No	1.1
15-19	Yes	Yes	Yes	greater than R6,000 pm	Yes	04-Feb	Yes	one	Yes	Yes	No	22	No	41	No	1.8
20-24	No	Yes	Yes	R501 - R2,500 pm	Yes	04-Feb	Yes	none	.	Yes	No	23	.	23	No	1.9
20-24	Yes	No	Yes	R501 - R2,500 pm	No	04-Feb	Yes	one	No	.	No	30	No	26	No	1.2
20-24	Yes	No	Yes	R0 - R500 pm	Yes	04-Feb	Yes	none	.	.	.	.	.	30	No	2
20-24	No	No	No	R501 - R2,500 pm	Yes	04-Feb	No	one	Yes	No	No	30	No	24	No	1.8
20-24	Yes	No	Yes	.	Yes	04-Feb	No	one	No	Yes	No	26	Yes	60	No	1.5
20-24	Yes	Yes	No	R501 - R2,500 pm	No	04-Feb	Yes	one	No	No	No	29	No	11	No	1.9
20-24	Yes	Yes	Yes	R501 - R2,500 pm	No	04-Feb	Yes	one	No	No	No	27	No	22	No	1.9
20-24	Yes	No	Yes	.	Yes	04-Feb	No	none	.	Yes	No	25	.	6	No	1.7
20-24	Yes	No	Yes	R501 - R2,500 pm	Yes	04-Feb	No	one	No	No	No	25	No	46	No	1.5
20-24	No	No	Yes	R501 - R2,500 pm	Yes	04-Feb	Yes	one	No	No	No	30	No	91	No	1.5
20-24	Yes	Yes	Yes	greater than R6,000 pm	Yes	04-Feb	Yes	one	No	Yes	No	26	No	14	Yes	0.6
20-24	No	No	Yes	R501 - R2,500 pm	Yes	04-Feb	Yes	one	Yes	Yes	No	25	No	81	No	1.4
20-24	Yes	Yes	Yes	R501 - R2,500 pm	No	04-Feb	Yes	one	No	Yes	No	28	No	9	No	2.3
20-24	No	Yes	Yes	R501 - R2,500 pm	Yes	04-Feb	Yes	one	No	No	No	24	No	9	No	2
20-24	No	Yes	No	R501 - R2,500 pm	No	04-Feb	Yes	one	No	No	No	27	No	20	Yes	1
20-24	No	No	Yes	R501 - R2,500 pm	Yes	04-Feb	No	one	No	Yes	No	29	No	6	No	1.1
15-19	No	Yes	Yes	R2,501 - R6,000 pm	Yes	04-Feb	Yes	one	No	No	No	23	No	26	Yes	0.6
20-24	No	Yes	Yes	R501 - R2,500 pm	No	04-Feb	No	2 or more	No	No	No	25	No	23	No	1.6
20-24	Yes	No	Yes	R501 - R2,500 pm	No	04-Feb	No	one	Yes	No	No	29	No	16	No	1.2
20-24	Yes	No	Yes	R0 - R500 pm	Yes	04-Feb	Yes	one	No	No	No	26	No	25	No	1.4
20-24	Yes	Yes	Yes	greater than R6,000 pm	Yes	04-Feb	No	none	.	.	.	.	.	13	No	1.4
20-24	No	Yes	Yes	.	No	04-Feb	No	2 or more	No	Yes	No	28	No	19	No	2
20-24	No	Yes	Yes	R0 - R500 pm	Yes	04-Feb	Yes	one	No	Yes	No	30	No	10	No	1.8
20-24	No	Yes	No	R2,501 - R6,000 pm	Yes	04-Feb	Yes	none	.	No	No	31	.	12	No	1.3
20-24	No	Yes	No	R501 - R2,500 pm	No	04-Feb	Yes	2 or more	No	Yes	No	38	No	28	No	1.2
20-24	Yes	Yes	No	R501 - R2,500 pm	No	04-Feb	Yes	one	No	No	No	25	No	22	No	1.8
15-19	Yes	Yes	Yes	R501 - R2,500 pm	No	04-Feb	No	one	No	No	No	26	No	13	No	1.6
20-24	Yes	No	No	R0 - R500 pm	No	04-Feb	Yes	one	No	Yes	No	22	No	30	No	1.8
15-19	Yes	Yes	Yes	R501 - R2,500 pm	Yes	04-Feb	Yes	one	Yes	Yes	No	27	No	11	No	1.7
20-24	Yes	No	Yes	R2,501 - R6,000 pm	Yes	04-Feb	Yes	none	.	.	.	.	.	6	No	1.4
20-24	Yes	No	Yes	R0 - R500 pm	No	04-Feb	Yes	one	No	No	No	29	No	30	No	1.8
20-24	Yes	Yes	Yes	R2,501 - R6,000 pm	Yes	04-Feb	No	one	No	No	No	31	No	8	No	1.9
20-24	No	No	Yes	R2,501 - R6,000 pm	Yes	04-Feb	Yes	one	No	Yes	No	26	No	74	No	1.4
20-24	Yes	No	Yes	R501 - R2,500 pm	Yes	04-Feb	Yes	one	No	Yes	No	25	No	16	No	1.4
15-19	No	No	Yes	R2,501 - R6,000 pm	Yes	04-Feb	Yes	one	No	Yes	No	25	No	78	No	1.5
15-19	Yes	No	Yes	R501 - R2,500 pm	Yes	04-Feb	Yes	one	Yes	No	No	32	No	91	No	1.2

20-24	No	Yes	No	R0 - R500 pm	Yes	04-Feb	Yes	one	No	No	No	36	No	14	No	1.9
20-24	No	Yes	Yes	R501 - R2,500 pm	No	04-Feb	No	one	Yes	Yes	Yes	27	No	11	Yes	1
15-19	No	Yes	Yes	.	No	04-Feb	Yes	one	No	Yes	No	27	No	11	No	1.9
20-24	Yes	Yes	No	R501 - R2,500 pm	Yes	04-Feb	No	one	No	No	No	32	No	11	No	1.2
20-24	Yes	No	Yes	R2,501 - R6,000 pm	No	04-Feb	No	one	No	Yes	No	26	No	91	No	1.4
20-24	Yes	No	Yes	R2,501 - R6,000 pm	Yes	04-Feb	Yes	none	.	.	.	.	.	9	No	1.2
15-19	No	Yes	Yes	R501 - R2,500 pm	Yes	04-Feb	Yes	one	Yes	No	No	22	No	12	No	1.9
15-19	Yes	No	Yes	greater than R6,000 pm	Yes	04-Feb	No	one	No	No	No	30	No	102	No	1.5
15-19	Yes	Yes	Yes	greater than R6,000 pm	No	04-Feb	Yes	one	No	Yes	No	20	No	17	No	1.4
20-24	No	No	Yes	R501 - R2,500 pm	Yes	04-Feb	Yes	one	No	Yes	No	26	No	58	No	1.9
20-24	No	Yes	Yes	R2,501 - R6,000 pm	Yes	04-Feb	Yes	one	No	Yes	No	29	No	60	No	1.2
20-24	No	Yes	No	R501 - R2,500 pm	No	04-Feb	No	one	No	No	No	26	Yes	10	No	1.3
15-19	No	No	No	R501 - R2,500 pm	Yes	04-Feb	Yes	2 or more	No	No	No	47	No	67	No	1.5
20-24	Yes	No	Yes	R501 - R2,500 pm	Yes	04-Feb	Yes	one	No	No	No	28	No	53	No	1.5
20-24	Yes	Yes	Yes	R501 - R2,500 pm	No	04-Feb	Yes	one	No	No	No	35	No	10	No	1.6
20-24	Yes	Yes	No	R501 - R2,500 pm	Yes	04-Feb	Yes	one	No	Yes	No	42	No	10	No	1.9
20-24	No	No	No	R2,501 - R6,000 pm	Yes	04-Feb	Yes	none	.	No	No	31	.	77	No	1.3
20-24	Yes	Yes	Yes	.	Yes	04-Feb	Yes	one	No	No	No	27	No	6	No	1.4
20-24	Yes	Yes	Yes	R501 - R2,500 pm	Yes	04-Feb	Yes	one	No	No	No	27	No	10	No	1.8
20-24	Yes	No	Yes	R501 - R2,500 pm	Yes	04-Feb	Yes	one	Yes	No	No	22	No	69	No	1.2
15-19	Yes	No	No	R501 - R2,500 pm	Yes	04-Feb	Yes	2 or more	No	No	No	29	Yes	62	Yes	0.7
15-19	Yes	No	Yes	R2,501 - R6,000 pm	Yes	04-Feb	Yes	one	No	Yes	No	28	Yes	51	No	1.4
20-24	Yes	Yes	Yes	R2,501 - R6,000 pm	No	04-Feb	Yes	one	Yes	No	Yes	26	No	10	No	1.8
20-24	No	No	Yes	R2,501 - R6,000 pm	Yes	04-Feb	Yes	one	No	Yes	No	27	No	27	No	2
20-24	Yes	Yes	No	R501 - R2,500 pm	Yes	04-Feb	Yes	one	No	No	No	29	No	11	No	1.7
15-19	No	No	Yes	greater than R6,000 pm	No	04-Feb	No	one	Yes	Yes	No	20	No	16	No	1.2
20-24	Yes	Yes	Yes	R0 - R500 pm	No	04-Feb	Yes	one	No	Yes	No	26	No	46	No	2
15-19	No	Yes	Yes	R501 - R2,500 pm	No	04-Feb	Yes	one	No	Yes	No	26	No	24	No	1.6
20-24	Yes	Yes	Yes	R2,501 - R6,000 pm	Yes	04-Feb	Yes	one	No	No	No	30	No	22	No	2.2
20-24	No	No	Yes	R501 - R2,500 pm	No	04-Feb	No	one	No	No	No	28	No	33	No	1.1
20-24	Yes	Yes	Yes	R501 - R2,500 pm	Yes	04-Feb	Yes	one	Yes	Yes	No	27	No	14	No	1.2
20-24	Yes	No	Yes	greater than R6,000 pm	No	04-Feb	Yes	none	.	No	No	29	.	54	No	1.3
20-24	Yes	Yes	Yes	.	Yes	04-Feb	No	one	No	Yes	No	28	No	13	No	2.1
15-19	Yes	No	Yes	.	Yes	04-Feb	No	one	No	.	No	23	No	52	No	1.5
15-19	Yes	Yes	Yes	.	Yes	04-Feb	No	none	.	.	.	.	.	10	No	1.9
20-24	No	No	Yes	R501 - R2,500 pm	Yes	04-Feb	Yes	none	.	Yes	No	24	.	14	No	1.4
20-24	Yes	No	Yes	R0 - R500 pm	No	04-Feb	Yes	one	No	Yes	No	36	No	24	No	1.3
20-24	Yes	Yes	No	R2,501 - R6,000 pm	No	04-Feb	Yes	one	No	Yes	No	50	No	15	No	1.9
20-24	No	Yes	No	R0 - R500 pm	No	04-Feb	No	one	No	Yes	No	33	No	23	No	1.8
15-19	No	Yes	Yes	R501 - R2,500 pm	Yes	04-Feb	Yes	one	No	No	No	19	Yes	15	No	1.8
20-24	Yes	Yes	Yes	.	No	04-Feb	No	one	No	No	No	29	Yes	17	No	1.1
20-24	No	Yes	Yes	R501 - R2,500 pm	Yes	04-Feb	Yes	one	Yes	Yes	No	28	No	11	No	1.8

20-24	Yes	Yes	Yes	R0 - R500 pm	Yes	04-Feb	No	one	Yes	No	No	22	No	16	No	1.8
20-24	Yes	Yes	Yes	R501 - R2,500 pm	Yes	04-Feb	Yes	one	No	Yes	No	26	No	20	No	1.6
15-19	Yes	Yes	Yes	R2,501 - R6,000 pm	Yes	04-Feb	Yes	one	Yes	No	No	30	No	12	No	1.9
15-19	Yes	No	Yes	R0 - R500 pm	Yes	04-Feb	Yes	one	No	Yes	No	30	No	18	Yes	1.2
20-24	Yes	Yes	Yes	R501 - R2,500 pm	No	04-Feb	Yes	one	No	Yes	No	24	No	7	No	1.8
20-24	No	Yes	No	R2,501 - R6,000 pm	Yes	04-Feb	Yes	2 or more	No	Yes	No	27	No	10	No	1.4
20-24	Yes	No	Yes	R501 - R2,500 pm	Yes	04-Feb	Yes	one	Yes	No	No	26	No	11	No	1.3
15-19	No	Yes	Yes	greater than R6,000 pm	No	04-Feb	Yes	none	.	.	.	.	.	32	No	1.5
20-24	No	No	Yes	R501 - R2,500 pm	No	04-Feb	No	one	No	Yes	No	29	No	72	Yes	0.9
20-24	Yes	Yes	Yes	R501 - R2,500 pm	Yes	04-Feb	Yes	one	No	No	No	30	No	33	No	1.8
15-19	No	Yes	Yes	R2,501 - R6,000 pm	Yes	04-Feb	Yes	none	.	.	.	.	.	22	No	1.4
20-24	No	Yes	No	R501 - R2,500 pm	Yes	04-Feb	Yes	one	No	No	No	25	No	9	No	1.3
20-24	Yes	Yes	Yes	R501 - R2,500 pm	No	04-Feb	Yes	one	No	No	No	29	No	7	No	1.9
20-24	No	No	Yes	R501 - R2,500 pm	Yes	04-Feb	Yes	none	.	Yes	No	24	.	13	No	1.2
20-24	Yes	Yes	Yes	greater than R6,000 pm	Yes	04-Feb	Yes	2 or more	No	Yes	No	27	No	9	No	1.6
15-19	No	No	No	R501 - R2,500 pm	Yes	04-Feb	Yes	2 or more	No	Yes	No	28	No	66	No	1.4
20-24	Yes	Yes	Yes	R2,501 - R6,000 pm	Yes	04-Feb	Yes	one	No	Yes	No	31	No	22	No	1.9
15-19	No	No	Yes	R2,501 - R6,000 pm	Yes	04-Feb	Yes	none	.	No	No	22	.	11	No	1.1
20-24	Yes	No	Yes	R2,501 - R6,000 pm	Yes	04-Feb	Yes	one	No	Yes	No	26	No	81	No	1.3
20-24	Yes	Yes	Yes	R501 - R2,500 pm	No	04-Feb	Yes	one	No	Yes	No	26	No	30	No	1.4
20-24	No	No	Yes	R2,501 - R6,000 pm	Yes	04-Feb	Yes	one	No	Yes	No	22	No	51	No	1.4
20-24	Yes	Yes	No	R501 - R2,500 pm	Yes	04-Feb	Yes	one	No	Yes	No	30	No	27	No	1.3
20-24	Yes	Yes	No	R501 - R2,500 pm	Yes	04-Feb	.	none	.	Yes	No	24	.	26	No	1.3
20-24	Yes	No	Yes	R2,501 - R6,000 pm	Yes	04-Feb	No	none	.	.	No	27	.	23	No	1.5
20-24	Yes	Yes	Yes	R2,501 - R6,000 pm	No	04-Feb	Yes	one	No	No	No	28	No	20	No	2.4
20-24	Yes	Yes	Yes	greater than R6,000 pm	Yes	04-Feb	No	one	No	Yes	No	29	No	36	No	1.3
15-19	No	Yes	Yes	R501 - R2,500 pm	Yes	04-Feb	Yes	2 or more	No	Yes	No	22	No	32	No	1.8
20-24	No	No	Yes	R2,501 - R6,000 pm	No	04-Feb	Yes	one	No	Yes	No	28	No	66	No	1.3
20-24	No	Yes	Yes	R2,501 - R6,000 pm	No	04-Feb	Yes	one	No	Yes	No	30	No	8	No	1.8
15-19	No	Yes	Yes	R2,501 - R6,000 pm	No	04-Feb	Yes	one	No	No	No	38	No	20	No	1.4
20-24	Yes	Yes	Yes	R501 - R2,500 pm	Yes	04-Feb	Yes	one	No	No	No	26	No	9	No	1.2
20-24	No	Yes	Yes	.	Yes	04-Feb	No	one	Yes	Yes	No	24	No	14	No	1.6
15-19	Yes	Yes	Yes	greater than R6,000 pm	No	04-Feb	Yes	one	Yes	Yes	No	50	No	11	Yes	1.1
20-24	No	Yes	Yes	R0 - R500 pm	Yes	04-Feb	Yes	2 or more	Yes	No	No	32	No	9	No	1.6
20-24	No	No	Yes	R2,501 - R6,000 pm	Yes	04-Feb	Yes	one	No	No	No	24	No	87	No	1.4
15-19	No	No	Yes	R501 - R2,500 pm	Yes	04-Feb	Yes	one	No	Yes	No	22	No	44	No	1.8
15-19	Yes	Yes	No	R501 - R2,500 pm	No	04-Feb	Yes	one	No	Yes	No	24	No	11	Yes	0.9
15-19	No	Yes	Yes	R501 - R2,500 pm	Yes	04-Feb	Yes	one	No	Yes	No	22	No	16	No	1.6
15-19	No	Yes	Yes	R501 - R2,500 pm	No	04-Feb	Yes	one	No	No	No	25	No	25	Yes	1
15-19	No	No	Yes	R501 - R2,500 pm	Yes	04-Feb	No	one	No	No	No	25	No	30	No	1.5
20-24	Yes	Yes	No	R501 - R2,500 pm	No	04-Feb	No	one	No	Yes	No	25	Yes	12	No	1.8
15-19	Yes	Yes	Yes	.	Yes	04-Feb	No	one	No	Yes	No	29	No	22	Yes	1.1

20-24	Yes	Yes	Yes	R501 - R2,500 pm	Yes	04-Feb	Yes	one	No	Yes	No	35	No	15	No	1.3
20-24	Yes	No	Yes	R2,501 - R6,000 pm	No	04-Feb	No	one	No	No	No	25	No	23	No	1.5
20-24	No	No	Yes	R501 - R2,500 pm	No	04-Feb	Yes	one	No	Yes	No	28	No	44	No	1.3
15-19	No	No	Yes	R501 - R2,500 pm	Yes	04-Feb	Yes	one	No	Yes	No	23	No	106	No	1.7
20-24	No	Yes	Yes	R501 - R2,500 pm	Yes	04-Feb	Yes	one	No	No	No	28	No	11	No	1.8
20-24	No	Yes	Yes	R501 - R2,500 pm	No	04-Feb	Yes	one	No	No	No	28	No	11	No	1.9
20-24	Yes	No	No	.	Yes	04-Feb	No	one	No	Yes	No	36	No	30	No	1.4
20-24	Yes	No	Yes	R2,501 - R6,000 pm	Yes	04-Feb	No	one	Yes	.	No	26	Yes	7	No	1.1
20-24	Yes	No	No	R501 - R2,500 pm	Yes	04-Feb	Yes	one	No	Yes	No	27	Yes	42	Yes	0.8
20-24	No	No	No	R501 - R2,500 pm	No	04-Feb	No	one	No	No	No	24	No	25	No	1.8
20-24	Yes	No	Yes	R2,501 - R6,000 pm	Yes	04-Feb	Yes	none	.	.	.	.	.	80	No	1.4
20-24	No	Yes	Yes	R501 - R2,500 pm	Yes	04-Feb	Yes	one	No	Yes	No	23	No	14	No	1.2
15-19	Yes	Yes	Yes	R2,501 - R6,000 pm	Yes	04-Feb	No	one	Yes	Yes	No	22	No	16	No	1.9
20-24	Yes	No	Yes	R501 - R2,500 pm	Yes	04-Feb	Yes	one	No	No	No	51	No	25	No	2.3
20-24	Yes	No	Yes	R501 - R2,500 pm	Yes	04-Feb	Yes	one	Yes	No	No	26	No	75	No	2.1
15-19	Yes	No	Yes	R501 - R2,500 pm	Yes	04-Feb	No	one	Yes	Yes	No	24	No	12	No	1.2
20-24	Yes	Yes	Yes	.	Yes	04-Feb	Yes	2 or more	No	No	No	26	No	24	No	1.8
20-24	Yes	Yes	Yes	R501 - R2,500 pm	Yes	04-Feb	Yes	one	No	Yes	No	30	No	26	No	1.2
20-24	Yes	No	No	R501 - R2,500 pm	No	04-Feb	Yes	one	No	No	No	28	No	29	No	2.1
15-19	Yes	No	Yes	greater than R6,000 pm	Yes	04-Feb	Yes	one	No	Yes	Yes	24	No	11	No	1.1
15-19	No	No	Yes	R2,501 - R6,000 pm	Yes	04-Feb	Yes	one	No	Yes	No	20	No	22	No	1.3
20-24	Yes	No	Yes	R2,501 - R6,000 pm	Yes	04-Feb	Yes	2 or more	No	Yes	No	21	No	32	No	1.5
15-19	No	Yes	Yes	R0 - R500 pm	Yes	04-Feb	Yes	one	No	No	No	22	No	12	No	2.2
20-24	Yes	Yes	Yes	R501 - R2,500 pm	Yes	04-Feb	Yes	2 or more	No	No	No	29	No	12	Yes	0.9
20-24	Yes	Yes	No	R0 - R500 pm	Yes	04-Feb	Yes	none	.	No	No	24	.	19	Yes	0.8
15-19	No	No	Yes	R2,501 - R6,000 pm	No	04-Feb	Yes	one	Yes	No	No	19	No	106	No	1.3
20-24	Yes	No	No	.	Yes	04-Feb	Yes	one	No	No	No	23	No	28	No	1.7
20-24	No	No	No	R501 - R2,500 pm	Yes	04-Feb	Yes	one	Yes	No	No	24	No	14	No	1.4
20-24	Yes	Yes	Yes	R501 - R2,500 pm	No	04-Feb	Yes	one	No	Yes	No	26	No	12	No	1.4
20-24	No	No	Yes	R501 - R2,500 pm	Yes	04-Feb	Yes	one	Yes	No	No	25	No	46	No	1.4
20-24	Yes	No	Yes	R2,501 - R6,000 pm	Yes	04-Feb	No	one	Yes	No	No	23	No	36	No	1.4
20-24	No	Yes	Yes	R501 - R2,500 pm	Yes	04-Feb	Yes	2 or more	No	No	No	37	No	29	No	1.9
20-24	Yes	Yes	Yes	greater than R6,000 pm	Yes	04-Feb	Yes	2 or more	No	Yes	No	32	No	7	No	1.2
20-24	Yes	Yes	Yes	R0 - R500 pm	Yes	04-Feb	Yes	one	No	Yes	No	28	No	7	No	1.9
15-19	Yes	No	Yes	.	Yes	04-Feb	No	one	No	Yes	No	23	No	65	No	1.4
20-24	Yes	No	Yes	R2,501 - R6,000 pm	Yes	04-Feb	Yes	one	No	Yes	No	31	No	91	No	1.6
20-24	Yes	No	Yes	R501 - R2,500 pm	Yes	04-Feb	No	one	No	No	No	30	No	24	No	1.9
15-19	No	Yes	Yes	R501 - R2,500 pm	Yes	04-Feb	Yes	one	No	Yes	No	22	No	10	No	1.3
20-24	Yes	No	Yes	R2,501 - R6,000 pm	Yes	04-Feb	No	one	No	Yes	No	46	No	69	No	1.2
15-19	No	Yes	No	R501 - R2,500 pm	Yes	04-Feb	Yes	one	No	No	No	27	No	31	No	1.2
20-24	Yes	No	Yes	R0 - R500 pm	Yes	04-Feb	Yes	one	No	Yes	No	30	No	18	No	1.7
20-24	No	Yes	Yes	R501 - R2,500 pm	Yes	04-Feb	Yes	one	No	Yes	No	29	No	8	No	1.9
20-24	Yes	Yes	Yes	R501 - R2,500 pm	No	04-Feb	Yes	one	No	Yes	No	25	No	11	Yes	0.9
20-24	Yes	No	Yes	R2,501 - R6,000 pm	Yes	04-Feb	Yes	one	No	No	No	23	No	78	No	1.4

15-19	Yes	Yes	Yes	R2,501 - R6,000 pm	Yes	04-Feb	No	one	No	Yes	No	27	No	18	Yes	0.8
20-24	Yes	No	Yes	R2,501 - R6,000 pm	Yes	04-Feb	No	one	No	No	No	30	No	63	No	1.3
20-24	Yes	No	Yes	greater than R6,000 pm	No	04-Feb	Yes	one	No	Yes	No	27	No	87	No	1.3
20-24	Yes	Yes	No	R501 - R2,500 pm	Yes	04-Feb	Yes	one	Yes	Yes	No	26	No	12	No	2.4
15-19	Yes	No	Yes	R0 - R500 pm	No	04-Feb	Yes	2 or more	No	No	No	22	No	47	No	1.3
15-19	Yes	Yes	Yes	greater than R6,000 pm	Yes	04-Feb	Yes	one	No	No	No	32	No	12	No	2.2
20-24	No	Yes	Yes	R501 - R2,500 pm	Yes	04-Feb	Yes	one	No	Yes	No	23	No	22	No	1.3
20-24	Yes	Yes	Yes	R0 - R500 pm	Yes	04-Feb	Yes	one	Yes	No	No	38	No	16	No	2.3
20-24	Yes	No	Yes	R501 - R2,500 pm	Yes	04-Feb	No	one	Yes	Yes	No	24	No	61	No	1.2
20-24	Yes	Yes	Yes	R501 - R2,500 pm	Yes	04-Feb	Yes	one	Yes	Yes	No	27	No	23	No	1.9
20-24	Yes	Yes	Yes	R501 - R2,500 pm	Yes	04-Feb	Yes	one	Yes	No	No	32	No	28	No	2
20-24	Yes	Yes	Yes	R2,501 - R6,000 pm	Yes	04-Feb	Yes	one	No	Yes	No	23	Yes	10	No	1.4
20-24	No	Yes	Yes	R2,501 - R6,000 pm	Yes	04-Feb	Yes	one	No	No	No	38	Yes	12	No	1.2
15-19	Yes	No	Yes	.	Yes	04-Feb	No	one	No	Yes	No	23	No	16	No	1.4
20-24	Yes	Yes	No	.	Yes	04-Feb	Yes	one	No	No	No	23	No	20	No	2
20-24	Yes	No	Yes	.	No	04-Feb	Yes	one	Yes	No	No	22	No	44	No	1.6
20-24	Yes	No	Yes	R0 - R500 pm	Yes	04-Feb	Yes	2 or more	No	No	No	32	No	23	No	1.4
15-19	No	No	Yes	greater than R6,000 pm	Yes	04-Feb	Yes	one	No	No	No	20	No	32	No	1.7
20-24	No	Yes	No	R501 - R2,500 pm	No	04-Feb	No	one	No	No	No	26	No	16	No	1.9
15-19	No	No	Yes	R2,501 - R6,000 pm	Yes	04-Feb	Yes	one	No	No	No	23	No	103	No	1.2
20-24	Yes	No	Yes	R2,501 - R6,000 pm	Yes	04-Feb	Yes	one	No	Yes	No	22	No	84	No	1.7
20-24	Yes	No	Yes	greater than R6,000 pm	Yes	04-Feb	Yes	one	No	Yes	No	27	No	21	No	1.6
20-24	No	Yes	Yes	R501 - R2,500 pm	Yes	04-Feb	Yes	one	No	No	No	27	No	11	No	1.3
15-19	Yes	Yes	Yes	R501 - R2,500 pm	Yes	04-Feb	No	one	No	No	No	25	No	13	No	1.6
20-24	Yes	Yes	Yes	R0 - R500 pm	No	04-Feb	Yes	none	.	No	No	24	.	36	No	1.8
20-24	Yes	No	Yes	R501 - R2,500 pm	Yes	04-Feb	Yes	none	.	Yes	No	25	.	57	No	1.6
20-24	Yes	Yes	Yes	greater than R6,000 pm	No	04-Feb	Yes	one	No	.	No	25	No	12	No	1.9
20-24	Yes	Yes	Yes	R0 - R500 pm	No	04-Feb	Yes	one	No	No	No	27	No	8	No	2.1
20-24	No	No	No	R501 - R2,500 pm	Yes	04-Feb	Yes	one	No	No	No	30	No	91	No	2.1
20-24	Yes	No	Yes	greater than R6,000 pm	Yes	04-Feb	Yes	none	.	Yes	No	31	.	76	No	1.2
20-24	No	Yes	Yes	R501 - R2,500 pm	Yes	04-Feb	Yes	one	No	Yes	Yes	25	No	16	No	1.9
20-24	Yes	No	Yes	R501 - R2,500 pm	Yes	04-Feb	Yes	one	Yes	No	No	30	No	47	No	1.5
20-24	Yes	Yes	Yes	R501 - R2,500 pm	No	04-Feb	Yes	one	No	Yes	No	21	No	19	No	1.2
20-24	No	No	Yes	R2,501 - R6,000 pm	Yes	04-Feb	Yes	one	No	Yes	No	23	Yes	65	No	1.2
20-24	Yes	No	Yes	R2,501 - R6,000 pm	Yes	04-Feb	Yes	one	No	Yes	No	26	No	16	No	1.5
20-24	No	Yes	No	R0 - R500 pm	No	04-Feb	Yes	none	.	.	.	.	.	20	Yes	0.9
20-24	Yes	Yes	Yes	R501 - R2,500 pm	No	04-Feb	No	2 or more	No	No	No	23	No	8	No	1.7
20-24	Yes	Yes	Yes	R501 - R2,500 pm	Yes	04-Feb	Yes	one	No	Yes	No	24	No	56	No	1.4
20-24	Yes	Yes	Yes	R501 - R2,500 pm	Yes	04-Feb	No	one	No	Yes	No	28	No	23	No	2.3
20-24	Yes	Yes	Yes	R501 - R2,500 pm	Yes	04-Feb	Yes	none	.	.	.	.	.	9	No	1.9

20-24	No	Yes	Yes	R0 - R500 pm	Yes	04-Feb	Yes	none	.	.	.	.	.	24	No	1.3
20-24	Yes	Yes	Yes	R501 - R2,500 pm	No	04-Feb	No	none	.	.	.	.	.	21	No	1.6
20-24	Yes	Yes	No	R2,501 - R6,000 pm	No	04-Feb	Yes	one	No	Yes	No	31	No	16	No	1.7
15-19	No	No	Yes	R2,501 - R6,000 pm	Yes	04-Feb	Yes	none	.	.	.	.	.	100	No	2.1
20-24	No	Yes	Yes	R501 - R2,500 pm	Yes	04-Feb	Yes	one	No	No	No	27	No	11	No	1.6
20-24	Yes	Yes	No	R0 - R500 pm	Yes	04-Feb	Yes	none	.	No	No	28	.	9	Yes	0.9
15-19	Yes	No	Yes	R501 - R2,500 pm	Yes	04-Feb	Yes	one	No	Yes	No	23	Yes	16	No	1.5
20-24	Yes	No	Yes	R501 - R2,500 pm	Yes	04-Feb	Yes	one	No	No	No	26	No	20	No	1.3
20-24	No	No	Yes	greater than R6,000 pm	Yes	04-Feb	Yes	none	.	Yes	No	21	.	53	Yes	1
15-19	No	Yes	Yes	R2,501 - R6,000 pm	No	04-Feb	Yes	one	No	No	No	25	No	22	No	1.7
20-24	Yes	Yes	Yes	greater than R6,000 pm	No	04-Feb	Yes	none	.	.	.	.	.	27	No	1.3
20-24	Yes	No	Yes	R501 - R2,500 pm	Yes	04-Feb	No	one	Yes	No	No	30	No	43	No	1.9
20-24	Yes	No	Yes	R501 - R2,500 pm	Yes	04-Feb	Yes	one	No	Yes	No	25	No	91	No	1.5
20-24	No	Yes	Yes	R501 - R2,500 pm	No	04-Feb	No	one	No	No	No	41	No	11	No	1.7
20-24	Yes	Yes	Yes	R501 - R2,500 pm	No	04-Feb	Yes	one	No	.	No	23	Yes	9	No	2.1
20-24	Yes	No	Yes	R2,501 - R6,000 pm	Yes	04-Feb	Yes	one	No	Yes	No	27	No	24	No	1.5
20-24	Yes	No	Yes	R0 - R500 pm	Yes	04-Feb	Yes	one	No	No	No	20	No	23	No	1.2
20-24	Yes	No	Yes	R0 - R500 pm	Yes	04-Feb	Yes	none	.	Yes	No	24	.	57	No	1.5
20-24	Yes	No	Yes	R501 - R2,500 pm	Yes	04-Feb	Yes	one	No	Yes	No	21	No	31	No	1.4
20-24	No	Yes	Yes	R0 - R500 pm	Yes	04-Feb	Yes	one	No	No	No	33	No	11	No	1.6
20-24	No	Yes	Yes	R2,501 - R6,000 pm	Yes	04-Feb	Yes	one	No	Yes	No	32	No	11	No	1.4
20-24	Yes	No	Yes	R501 - R2,500 pm	Yes	04-Feb	Yes	one	No	Yes	No	32	No	14	No	2.4
20-24	No	No	Yes	R501 - R2,500 pm	Yes	04-Feb	Yes	one	No	Yes	No	32	Yes	22	No	1.4
15-19	No	Yes	No	R501 - R2,500 pm	Yes	04-Feb	Yes	one	No	No	No	22	No	61	Yes	1
20-24	Yes	No	Yes	R2,501 - R6,000 pm	Yes	04-Feb	Yes	one	No	Yes	No	25	No	50	No	1.3
20-24	No	Yes	No	R501 - R2,500 pm	Yes	04-Feb	Yes	2 or more	No	No	No	40	No	19	No	1.9
20-24	Yes	Yes	Yes	R501 - R2,500 pm	No	04-Feb	Yes	one	No	No	No	25	No	13	No	1.8
20-24	No	Yes	Yes	R2,501 - R6,000 pm	Yes	04-Feb	Yes	one	No	Yes	No	28	Yes	47	No	1.4
20-24	No	Yes	Yes	R501 - R2,500 pm	Yes	04-Feb	Yes	one	No	Yes	No	25	No	18	Yes	0.7
20-24	Yes	Yes	Yes	R501 - R2,500 pm	No	04-Feb	Yes	one	No	No	No	24	No	8	No	1.9
20-24	No	Yes	Yes	R501 - R2,500 pm	No	04-Feb	No	one	No	No	No	29	No	11	Yes	0.8
15-19	No	No	Yes	R0 - R500 pm	No	04-Feb	No	one	Yes	Yes	No	22	No	32	No	2.1
20-24	No	Yes	Yes	R0 - R500 pm	Yes	04-Feb	Yes	one	No	No	No	30	No	13	No	1.8
20-24	No	No	Yes	greater than R6,000 pm	Yes	04-Feb	Yes	one	No	No	No	30	No	23	No	1.8
20-24	Yes	No	Yes	R2,501 - R6,000 pm	Yes	04-Feb	Yes	one	No	Yes	No	40	No	12	No	1.4
20-24	Yes	No	Yes	R501 - R2,500 pm	No	04-Feb	No	one	No	Yes	No	27	No	30	No	1.5
20-24	Yes	Yes	Yes	R0 - R500 pm	Yes	04-Feb	Yes	none	.	Yes	No	23	.	56	No	1.3
20-24	Yes	No	Yes	R501 - R2,500 pm	Yes	04-Feb	No	one	No	No	No	24	No	22	Yes	0.7
15-19	Yes	Yes	Yes	R501 - R2,500 pm	Yes	04-Feb	No	one	No	Yes	No	21	No	11	No	2.1
20-24	No	No	Yes	R501 - R2,500 pm	Yes	04-Feb	Yes	one	No	No	No	24	No	30	No	1.4
20-24	Yes	Yes	Yes	R2,501 - R6,000 pm	Yes	04-Feb	No	one	No	Yes	No	28	Yes	27	No	1.4
20-24	No	Yes	Yes	R0 - R500 pm	Yes	04-Feb	Yes	one	No	Yes	No	26	No	16	No	1.9
20-24	Yes	No	Yes	.	Yes	04-Feb	No	one	No	Yes	No	26	Yes	6	No	1.7

20-24	Yes	Yes	Yes	.	No	04-Feb	No	one	No	Yes	No	29	No	30	No	1.4
15-19	Yes	Yes	No	R501 - R2,500 pm	No	04-Feb	Yes	one	No	Yes	No	24	No	58	No	1.2
20-24	No	Yes	Yes	R501 - R2,500 pm	Yes	04-Feb	Yes	2 or more	No	No	No	27	No	11	No	2
20-24	Yes	No	Yes	greater than R6,000 pm	No	04-Feb	No	one	No	Yes	No	25	No	61	No	1.3
15-19	Yes	No	No	R0 - R500 pm	Yes	04-Feb	Yes	one	No	Yes	No	19	No	47	No	1.5
20-24	Yes	Yes	Yes	R501 - R2,500 pm	Yes	04-Feb	Yes	one	No	Yes	No	29	No	56	No	1.8
20-24	Yes	No	Yes	.	No	04-Feb	Yes	one	No	Yes	No	23	No	24	No	1.9
20-24	Yes	Yes	Yes	R501 - R2,500 pm	Yes	04-Feb	Yes	2 or more	No	No	No	29	No	18	No	1.9
15-19	No	Yes	Yes	R501 - R2,500 pm	Yes	04-Feb	Yes	one	No	No	No	26	No	12	No	1.4
15-19	No	No	Yes	R2,501 - R6,000 pm	No	04-Feb	No	one	No	Yes	No	21	No	53	No	1.3
20-24	Yes	Yes	Yes	R0 - R500 pm	Yes	04-Feb	Yes	one	No	No	No	30	No	9	No	1.6
20-24	Yes	Yes	Yes	.	No	04-Feb	Yes	2 or more	No	Yes	No	25	No	36	No	1.2
15-19	No	No	Yes	R501 - R2,500 pm	Yes	04-Feb	No	one	Yes	No	No	22	No	73	No	1.3
15-19	Yes	No	Yes	R501 - R2,500 pm	Yes	04-Feb	Yes	one	No	No	Yes	27	No	18	Yes	0.9
20-24	No	Yes	No	R0 - R500 pm	No	04-Feb	No	one	Yes	Yes	No	26	No	17	No	2.1
15-19	Yes	No	Yes	R2,501 - R6,000 pm	No	04-Feb	Yes	one	No	Yes	No	29	No	22	No	1.4
20-24	Yes	Yes	Yes	R501 - R2,500 pm	No	04-Feb	Yes	one	No	No	No	26	No	31	Yes	0.7
20-24	Yes	No	Yes	R501 - R2,500 pm	No	04-Feb	No	one	No	Yes	Yes	24	No	30	No	1.8
20-24	Yes	Yes	No	R0 - R500 pm	Yes	04-Feb	Yes	one	No	No	No	29	No	20	No	1.4
20-24	Yes	Yes	Yes	R0 - R500 pm	No	04-Feb	No	one	No	Yes	No	26	No	12	No	1.6
20-24	No	No	Yes	R501 - R2,500 pm	No	04-Feb	Yes	none	.	Yes	No	25	.	24	Yes	0.7
20-24	Yes	No	Yes	R2,501 - R6,000 pm	Yes	04-Feb	Yes	none	.	No	No	25	.	77	No	1.4
20-24	Yes	Yes	Yes	R0 - R500 pm	Yes	04-Feb	Yes	one	No	Yes	No	28	No	8	No	2.2
20-24	No	Yes	Yes	R2,501 - R6,000 pm	Yes	04-Feb	Yes	one	Yes	Yes	No	24	No	27	No	1.3
20-24	Yes	Yes	Yes	greater than R6,000 pm	Yes	04-Feb	No	none	.	Yes	No	26	.	11	No	1.3
15-19	No	No	No	R501 - R2,500 pm	No	04-Feb	Yes	one	No	No	No	25	No	12	No	1.1
20-24	Yes	Yes	No	R501 - R2,500 pm	No	04-Feb	Yes	one	No	No	No	25	No	10	No	2.2
20-24	Yes	Yes	Yes	R0 - R500 pm	Yes	04-Feb	Yes	2 or more	No	Yes	No	28	No	15	No	1.3
20-24	No	Yes	Yes	R0 - R500 pm	Yes	04-Feb	Yes	2 or more	No	No	Yes	30	No	8	No	1.6
20-24	No	No	Yes	R2,501 - R6,000 pm	Yes	04-Feb	Yes	none	.	Yes	No	30	.	53	No	1.4
15-19	Yes	Yes	Yes	R501 - R2,500 pm	No	04-Feb	Yes	2 or more	No	.	No	22	No	43	No	1.3
20-24	Yes	Yes	No	R501 - R2,500 pm	Yes	04-Feb	Yes	one	Yes	No	No	32	No	8	No	1
20-24	Yes	Yes	Yes	.	Yes	04-Feb	No	one	No	No	No	34	No	28	Yes	0.6
20-24	Yes	No	Yes	R501 - R2,500 pm	Yes	04-Feb	No	2 or more	No	No	No	29	No	30	No	1.8
20-24	Yes	Yes	No	R501 - R2,500 pm	Yes	04-Feb	Yes	none	.	.	.	.	.	14	No	1.2
20-24	No	Yes	Yes	R501 - R2,500 pm	No	04-Feb	Yes	one	No	No	No	26	No	17	No	1.3
20-24	No	Yes	Yes	R501 - R2,500 pm	No	04-Feb	Yes	one	No	No	No	33	No	43	No	1.9
20-24	Yes	Yes	Yes	R2,501 - R6,000 pm	No	04-Feb	Yes	one	No	Yes	No	28	No	10	Yes	1.1
15-19	Yes	No	Yes	R501 - R2,500 pm	No	04-Feb	No	one	No	Yes	No	25	No	25	No	2.7
20-24	Yes	Yes	Yes	greater than R6,000 pm	No	04-Feb	Yes	one	No	Yes	No	25	Yes	9	No	1.9
20-24	Yes	Yes	Yes	R2,501 - R6,000 pm	Yes	04-Feb	Yes	one	No	No	No	33	No	43	No	1.4
20-24	Yes	No	Yes	R0 - R500 pm	Yes	04-Feb	Yes	one	Yes	Yes	No	24	No	53	No	1.4
20-24	Yes	Yes	No	R0 - R500 pm	Yes	04-Feb	Yes	one	No	No	No	38	No	10	No	1.5

20-24	Yes	No	Yes	R501 - R2,500 pm	Yes	04-Feb	Yes	one	No	No	No	28	No	55	No	1.4
15-19	No	No	Yes	R501 - R2,500 pm	Yes	04-Feb	Yes	one	No	No	No	21	No	27	No	1.7
15-19	No	Yes	Yes	R2,501 - R6,000 pm	Yes	04-Feb	Yes	none	.	.	.	.	.	10	Yes	0.8
15-19	Yes	Yes	Yes	.	Yes	04-Feb	Yes	one	No	Yes	No	24	No	26	No	1.9
20-24	Yes	Yes	Yes	R2,501 - R6,000 pm	Yes	04-Feb	Yes	one	Yes	Yes	No	32	No	24	No	2.2
20-24	Yes	Yes	No	.	Yes	04-Feb	Yes	one	Yes	No	No	27	Yes	38	No	1.3
15-19	Yes	Yes	Yes	R0 - R500 pm	No	04-Feb	No	one	No	No	No	25	Yes	13	No	2.1
20-24	Yes	Yes	No	R501 - R2,500 pm	Yes	04-Feb	Yes	one	No	Yes	No	27	No	16	No	1.2
20-24	Yes	No	Yes	greater than R6,000 pm	Yes	04-Feb	No	one	No	No	No	25	No	53	No	1.4
20-24	Yes	Yes	Yes	R501 - R2,500 pm	Yes	04-Feb	Yes	one	No	Yes	No	28	No	11	No	1.8
20-24	No	Yes	Yes	R501 - R2,500 pm	Yes	04-Feb	Yes	one	No	No	No	26	No	24	No	1.2
20-24	Yes	Yes	Yes	R501 - R2,500 pm	No	04-Feb	No	2 or more	Yes	No	No	24	Yes	24	No	1.2
15-19	Yes	Yes	Yes	R2,501 - R6,000 pm	No	04-Feb	No	one	No	No	No	25	No	52	No	1.3
15-19	No	Yes	Yes	R2,501 - R6,000 pm	Yes	04-Feb	No	one	No	Yes	No	21	No	31	No	1.3
20-24	No	Yes	Yes	R501 - R2,500 pm	Yes	04-Feb	No	one	Yes	Yes	No	27	No	34	No	1.5
15-19	No	Yes	Yes	R501 - R2,500 pm	Yes	04-Feb	No	one	No	No	No	20	No	16	No	1.3
20-24	Yes	Yes	Yes	R501 - R2,500 pm	Yes	04-Feb	Yes	one	Yes	Yes	No	24	Yes	25	No	1.6
20-24	Yes	Yes	Yes	R2,501 - R6,000 pm	No	04-Feb	Yes	one	No	No	No	29	No	14	No	1.6
15-19	No	Yes	Yes	R2,501 - R6,000 pm	Yes	04-Feb	Yes	one	No	No	No	22	No	36	No	1.1
15-19	Yes	Yes	Yes	R501 - R2,500 pm	Yes	04-Feb	Yes	one	No	Yes	No	21	No	24	No	1.6
20-24	Yes	Yes	Yes	R501 - R2,500 pm	Yes	04-Feb	No	one	Yes	No	No	29	No	59	No	1.3
20-24	No	Yes	Yes	R501 - R2,500 pm	No	04-Feb	No	one	No	Yes	No	23	No	18	No	1.6
15-19	No	Yes	Yes	R2,501 - R6,000 pm	Yes	04-Feb	No	none	.	Yes	No	17	.	33	No	1.6
20-24	No	Yes	No	R501 - R2,500 pm	Yes	04-Feb	Yes	one	No	Yes	No	29	No	18	No	1.6
20-24	Yes	Yes	Yes	R501 - R2,500 pm	No	04-Feb	Yes	one	No	Yes	No	30	No	45	No	1.3
20-24	Yes	Yes	Yes	R0 - R500 pm	Yes	04-Feb	Yes	none	.	Yes	No	22	.	10	No	1.3
15-19	No	Yes	Yes	R501 - R2,500 pm	Yes	04-Feb	Yes	one	Yes	Yes	No	22	No	12	No	1.3
20-24	Yes	Yes	Yes	R2,501 - R6,000 pm	Yes	04-Feb	Yes	one	Yes	Yes	No	26	No	14	No	1.3
20-24	Yes	Yes	Yes	R501 - R2,500 pm	Yes	04-Feb	Yes	none	.	.	.	.	.	21	Yes	1
20-24	No	Yes	Yes	R501 - R2,500 pm	Yes	04-Feb	Yes	one	No	No	No	30	No	11	No	1.6
20-24	Yes	Yes	Yes	R2,501 - R6,000 pm	Yes	04-Feb	Yes	one	No	No	No	27	No	53	No	1.6
20-24	Yes	Yes	Yes	R501 - R2,500 pm	Yes	04-Feb	No	one	No	Yes	No	29	No	11	No	1.3
20-24	Yes	Yes	Yes	R501 - R2,500 pm	Yes	04-Feb	Yes	one	Yes	Yes	No	28	No	20	No	1.7
15-19	No	Yes	Yes	R501 - R2,500 pm	Yes	04-Feb	No	one	No	Yes	No	27	No	28	Yes	0.8
20-24	No	Yes	Yes	R501 - R2,500 pm	Yes	04-Feb	No	one	No	Yes	No	24	No	21	Yes	0.9
20-24	Yes	Yes	No	greater than R6,000 pm	Yes	04-Feb	Yes	one	No	Yes	No	30	No	44	No	1.3
15-19	No	Yes	Yes	R501 - R2,500 pm	Yes	04-Feb	No	one	No	Yes	No	21	Yes	18	Yes	0.8
15-19	No	Yes	Yes	R2,501 - R6,000 pm	Yes	04-Feb	Yes	2 or more	No	No	No	25	Yes	21	No	1.1
20-24	No	Yes	Yes	R501 - R2,500 pm	Yes	04-Feb	Yes	one	Yes	No	No	28	No	29	No	1.3
20-24	Yes	Yes	Yes	R2,501 - R6,000 pm	Yes	04-Feb	Yes	one	No	No	No	29	No	41	No	1.3
20-24	No	Yes	Yes	R2,501 - R6,000 pm	Yes	04-Feb	Yes	one	No	Yes	No	24	No	11	No	1.5
15-19	Yes	Yes	Yes	R501 - R2,500 pm	No	04-Feb	No	2 or more	No	No	No	23	No	9	No	1.4
20-24	Yes	Yes	Yes	greater than R6,000 pm	Yes	04-Feb	Yes	one	No	No	No	30	No	10	No	1.3

20-24	Yes	Yes	Yes	R501 - R2,500 pm	No	04-Feb	Yes	one	No	Yes	No	25	No	29	No	1.3
20-24	Yes	Yes	Yes	R2,501 - R6,000 pm	Yes	04-Feb	Yes	one	No	No	No	30	Yes	10	No	1.3
20-24	Yes	Yes	Yes	R2,501 - R6,000 pm	Yes	04-Feb	No	one	No	Yes	No	25	No	58	No	2
20-24	No	Yes	Yes	R2,501 - R6,000 pm	Yes	04-Feb	Yes	one	No	No	Yes	26	No	10	No	1.2
20-24	Yes	Yes	Yes	R2,501 - R6,000 pm	No	04-Feb	Yes	one	No	Yes	No	27	No	11	No	1.6
20-24	No	No	Yes	R501 - R2,500 pm	No	04-Feb	No	none	.	.	.	.	.	22	No	2.1
20-24	No	No	Yes	R0 - R500 pm	Yes	04-Feb	Yes	one	Yes	Yes	No	25	No	31	No	2.1
15-19	No	No	Yes	R501 - R2,500 pm	Yes	04-Feb	Yes	2 or more	No	No	No	23	No	8	Yes	1
20-24	No	No	No	R501 - R2,500 pm	No	04-Feb	Yes	one	No	Yes	No	25	No	9	No	1.8
20-24	No	No	Yes	R2,501 - R6,000 pm	Yes	04-Feb	No	2 or more	No	No	No	32	No	38	No	1.8
20-24	Yes	No	Yes	R501 - R2,500 pm	Yes	04-Feb	Yes	one	No	Yes	No	30	No	20	Yes	0.9
20-24	Yes	No	Yes	R2,501 - R6,000 pm	Yes	04-Feb	No	one	No	Yes	No	26	No	32	No	1.8
15-19	Yes	No	Yes	R2,501 - R6,000 pm	No	04-Feb	No	one	No	Yes	No	24	No	21	No	1.3
20-24	No	No	No	R0 - R500 pm	Yes	04-Feb	Yes	none	.	No	No	22	.	12	No	1.3
20-24	No	No	No	R501 - R2,500 pm	Yes	04-Feb	Yes	2 or more	No	Yes	No	22	No	39	Yes	0.9
20-24	No	No	Yes	R2,501 - R6,000 pm	Yes	04-Feb	Yes	one	No	No	No	25	No	53	No	1.2
20-24	No	No	Yes	R501 - R2,500 pm	Yes	04-Feb	Yes	one	No	Yes	No	27	No	19	No	1.2
20-24	No	No	Yes	R501 - R2,500 pm	Yes	04-Feb	Yes	one	No	No	No	21	No	18	No	1.2
20-24	No	No	Yes	R2,501 - R6,000 pm	Yes	04-Feb	Yes	one	No	No	Yes	32	No	38	Yes	0.7
15-19	No	No	Yes	R501 - R2,500 pm	Yes	04-Feb	Yes	one	No	No	No	25	No	25	No	1.7
20-24	No	No	Yes	R501 - R2,500 pm	Yes	04-Feb	Yes	one	No	Yes	No	30	No	29	No	1.7
20-24	Yes	No	Yes	R501 - R2,500 pm	Yes	04-Feb	No	one	No	No	No	28	No	62	No	1.7
15-19	No	No	Yes	R2,501 - R6,000 pm	No	04-Feb	Yes	one	No	Yes	No	23	No	13	No	1.7
20-24	Yes	No	Yes	R501 - R2,500 pm	Yes	04-Feb	Yes	one	Yes	Yes	No	26	No	26	No	1.4
20-24	No	No	Yes	R501 - R2,500 pm	No	04-Feb	Yes	one	No	Yes	No	25	No	26	No	1.4
20-24	Yes	No	Yes	R2,501 - R6,000 pm	Yes	04-Feb	Yes	none	.	Yes	No	28	.	25	No	1.2
20-24	No	No	Yes	greater than R6,000 pm	Yes	04-Feb	Yes	one	No	.	No	29	No	13	No	1.2
15-19	Yes	No	Yes	R2,501 - R6,000 pm	No	04-Feb	No	none	.	Yes	No	26	.	21	No	1.7
20-24	No	No	Yes	R0 - R500 pm	No	04-Feb	Yes	one	No	No	No	26	No	30	No	1.7
15-19	No	No	No	R2,501 - R6,000 pm	Yes	04-Feb	No	one	No	Yes	No	21	No	45	No	1.9
15-19	No	No	Yes	R2,501 - R6,000 pm	No	04-Feb	Yes	one	No	No	No	23	No	29	No	1.9
20-24	No	No	No	R501 - R2,500 pm	Yes	04-Feb	Yes	one	Yes	Yes	No	30	No	20	No	1.7
20-24	Yes	No	No	R501 - R2,500 pm	Yes	04-Feb	Yes	one	No	No	No	28	No	43	No	1.8
15-19	No	No	Yes	R2,501 - R6,000 pm	Yes	04-Feb	Yes	one	No	Yes	No	21	No	53	No	1.8
20-24	Yes	No	Yes	R501 - R2,500 pm	Yes	04-Feb	Yes	one	No	Yes	No	27	No	26	No	1.7
20-24	No	No	Yes	R501 - R2,500 pm	No	04-Feb	Yes	none	.	No	No	18	.	26	No	1.5
20-24	Yes	No	Yes	greater than R6,000 pm	Yes	04-Feb	Yes	one	No	No	No	24	No	10	No	1.5
15-19	No	No	Yes	R501 - R2,500 pm	Yes	04-Feb	Yes	one	Yes	Yes	No	22	No	9	No	1.8
20-24	No	No	Yes	R2,501 - R6,000 pm	Yes	04-Feb	Yes	one	No	Yes	No	27	No	20	No	1.8
20-24	No	No	Yes	R501 - R2,500 pm	No	04-Feb	Yes	one	No	No	No	35	No	19	Yes	0.9
20-24	Yes	No	Yes	R501 - R2,500 pm	No	04-Feb	Yes	one	Yes	Yes	No	22	No	27	No	1.8
20-24	Yes	No	Yes	R2,501 - R6,000 pm	Yes	04-Feb	No	one	No	Yes	No	28	No	27	No	1.5
15-19	No	No	Yes	greater than R6,000 pm	Yes	04-Feb	No	one	No	Yes	No	21	No	37	No	1.5

20-24	Yes	Yes	No	R2,501 - R6,000 pm	No	04-Feb	No	one	Yes	Yes	No	30	No	17	No	1.2
15-19	Yes	Yes	Yes	greater than R6,000 pm	Yes	04-Feb	Yes	one	No	Yes	No	24	No	53	No	1.3
15-19	No	Yes	Yes	R2,501 - R6,000 pm	No	04-Feb	No	one	No	No	No	24	No	53	No	1.3
20-24	Yes	Yes	Yes	R501 - R2,500 pm	Yes	04-Feb	Yes	one	No	No	No	34	No	48	No	1.3
20-24	Yes	Yes	Yes	R501 - R2,500 pm	No	04-Feb	Yes	one	No	Yes	No	29	No	45	No	1.7
20-24	Yes	Yes	Yes	R501 - R2,500 pm	Yes	04-Feb	Yes	none	.	No	No	26	.	15	No	1.6
20-24	No	Yes	Yes	R501 - R2,500 pm	No	04-Feb	Yes	one	No	No	No	25	No	21	No	1.9
20-24	No	Yes	Yes	R2,501 - R6,000 pm	Yes	04-Feb	No	one	No	Yes	No	26	No	35	No	1.3
20-24	Yes	Yes	No	R501 - R2,500 pm	Yes	04-Feb	Yes	none	.	No	No	31	.	46	No	1.3
20-24	Yes	Yes	No	R501 - R2,500 pm	Yes	04-Feb	Yes	one	No	No	No	25	No	12	Yes	0.7
20-24	No	Yes	Yes	R501 - R2,500 pm	No	04-Feb	Yes	one	No	No	No	37	No	12	No	1.9
15-19	No	Yes	Yes	R2,501 - R6,000 pm	Yes	04-Feb	Yes	one	No	No	No	25	No	12	No	1.3
15-19	No	Yes	Yes	R501 - R2,500 pm	No	04-Feb	No	one	No	Yes	No	23	No	34	No	1.4
15-19	No	Yes	Yes	R2,501 - R6,000 pm	No	04-Feb	No	none	.	.	.	.	.	23	Yes	0.7
20-24	Yes	Yes	Yes	R501 - R2,500 pm	Yes	04-Feb	Yes	one	Yes	Yes	No	37	No	24	No	1.4
20-24	Yes	Yes	Yes	R501 - R2,500 pm	No	04-Feb	No	one	No	Yes	No	28	No	35	No	1.3
15-19	No	Yes	Yes	R2,501 - R6,000 pm	Yes	04-Feb	No	none	.	.	No	17	.	20	No	1.3
20-24	No	Yes	Yes	R0 - R500 pm	Yes	04-Feb	Yes	none	.	No	No	27	.	18	No	1.3
20-24	No	Yes	Yes	R2,501 - R6,000 pm	Yes	04-Feb	Yes	one	Yes	No	No	23	No	17	Yes	0.7
20-24	Yes	Yes	Yes	R501 - R2,500 pm	Yes	04-Feb	No	none	.	Yes	Yes	28	.	10	No	1.4
20-24	Yes	Yes	Yes	R2,501 - R6,000 pm	Yes	04-Feb	Yes	one	Yes	Yes	No	33	No	13	No	1.3
15-19	No	Yes	Yes	R501 - R2,500 pm	Yes	04-Feb	No	none	.	Yes	No	16	.	33	No	1.3
20-24	Yes	Yes	Yes	R2,501 - R6,000 pm	Yes	04-Feb	Yes	one	No	Yes	No	27	No	25	No	1.3
20-24	Yes	Yes	Yes	R2,501 - R6,000 pm	Yes	04-Feb	No	one	No	No	No	28	No	15	No	1.3
20-24	No	Yes	Yes	R501 - R2,500 pm	No	04-Feb	Yes	one	No	Yes	No	24	No	48	No	1.3
20-24	Yes	Yes	Yes	R2,501 - R6,000 pm	Yes	04-Feb	Yes	one	No	No	No	30	No	25	No	1.3
20-24	No	Yes	Yes	R501 - R2,500 pm	No	04-Feb	Yes	one	Yes	No	Yes	30	No	36	No	1.4
20-24	Yes	Yes	Yes	R501 - R2,500 pm	Yes	04-Feb	Yes	one	No	No	No	31	Yes	19	No	1.4
20-24	No	Yes	Yes	R501 - R2,500 pm	Yes	04-Feb	Yes	none	.	Yes	No	30	.	46	No	1.4
20-24	No	Yes	Yes	R501 - R2,500 pm	Yes	04-Feb	Yes	one	No	Yes	No	25	No	27	No	1.4
20-24	No	Yes	Yes	R501 - R2,500 pm	Yes	04-Feb	Yes	one	No	Yes	No	28	No	10	No	1.8
20-24	No	Yes	Yes	R0 - R500 pm	Yes	04-Feb	Yes	one	No	Yes	No	29	No	12	No	1.6
15-19	Yes	Yes	Yes	R501 - R2,500 pm	No	04-Feb	Yes	one	Yes	No	No	23	No	17	No	1.3
20-24	No	Yes	Yes	R2,501 - R6,000 pm	No	04-Feb	No	one	No	No	No	20	Yes	60	No	1.8
20-24	No	Yes	Yes	R501 - R2,500 pm	No	04-Feb	No	one	Yes	No	No	34	No	13	No	1.9
20-24	Yes	Yes	Yes	R501 - R2,500 pm	Yes	04-Feb	Yes	one	No	Yes	No	25	Yes	13	No	1.4
20-24	Yes	Yes	Yes	R2,501 - R6,000 pm	Yes	04-Feb	Yes	one	Yes	Yes	No	25	Yes	10	No	1.4
20-24	No	Yes	Yes	greater than R6,000 pm	Yes	04-Feb	Yes	one	Yes	Yes	No	32	No	31	No	1.4
20-24	Yes	Yes	Yes	greater than R6,000 pm	Yes	04-Feb	Yes	one	No	No	No	24	No	21	No	1.4
15-19	No	Yes	Yes	R501 - R2,500 pm	Yes	04-Feb	No	one	No	No	No	22	No	20	No	1.2
20-24	Yes	Yes	No	R0 - R500 pm	Yes	04-Feb	Yes	one	No	Yes	No	28	No	18	No	1.4
20-24	No	Yes	No	R501 - R2,500 pm	Yes	04-Feb	Yes	one	Yes	Yes	No	23	No	10	No	1.3
15-19	No	Yes	Yes	R2,501 - R6,000 pm	Yes	04-Feb	Yes	one	No	Yes	No	31	No	15	No	1.3

20-24	Yes	Yes	Yes	R501 - R2,500 pm	Yes	04-Feb	No	one	Yes	.	No	28	No	18	Yes	0.7
20-24	No	Yes	No	R2,501 - R6,000 pm	Yes	04-Feb	No	one	No	Yes	No	28	Yes	18	No	1.7
15-19	No	Yes	Yes	R0 - R500 pm	No	04-Feb	Yes	2 or more	.	No	No	22	No	27	No	1.4
20-24	No	Yes	Yes	R501 - R2,500 pm	Yes	04-Feb	Yes	one	No	Yes	No	30	No	55	No	1.4
20-24	Yes	Yes	Yes	greater than R6,000 pm	Yes	04-Feb	Yes	one	No	Yes	No	30	Yes	26	Yes	0.8
15-19	No	Yes	Yes	R2,501 - R6,000 pm	Yes	04-Feb	Yes	one	No	Yes	No	29	No	40	No	1.3
20-24	Yes	Yes	Yes	R0 - R500 pm	No	04-Feb	Yes	one	No	Yes	No	25	Yes	14	No	1.7
20-24	Yes	Yes	Yes	R2,501 - R6,000 pm	Yes	04-Feb	Yes	one	No	Yes	No	29	Yes	23	No	1.3
20-24	Yes	Yes	Yes	R2,501 - R6,000 pm	Yes	04-Feb	No	none	.	No	No	23	.	35	No	1.5
20-24	Yes	Yes	Yes	R501 - R2,500 pm	Yes	04-Feb	Yes	none	.	.	.	.	.	44	No	1.6
20-24	Yes	Yes	Yes	R2,501 - R6,000 pm	Yes	04-Feb	Yes	2 or more	No	Yes	No	25	No	22	No	2
20-24	No	No	Yes	R2,501 - R6,000 pm	Yes	04-Feb	Yes	none	.	.	.	.	.	60	No	1.5
20-24	Yes	No	Yes	R501 - R2,500 pm	Yes	04-Feb	Yes	one	Yes	No	No	28	No	53	No	1.9
20-24	Yes	No	Yes	R2,501 - R6,000 pm	Yes	04-Feb	Yes	one	No	.	No	35	No	53	No	1.5
15-19	No	No	Yes	R501 - R2,500 pm	No	04-Feb	Yes	one	No	Yes	Yes	24	No	38	Yes	0.7
15-19	No	No	Yes	R2,501 - R6,000 pm	Yes	04-Feb	Yes	one	No	No	No	28	No	53	No	1.9
15-19	No	No	Yes	R501 - R2,500 pm	No	04-Feb	Yes	one	No	Yes	No	26	No	12	No	1.5
20-24	Yes	No	Yes	greater than R6,000 pm	Yes	04-Feb	Yes	none	.	.	.	.	.	13	No	1.5
20-24	Yes	No	Yes	R2,501 - R6,000 pm	No	04-Feb	No	one	No	No	No	30	No	29	No	1.2
20-24	Yes	No	Yes	R501 - R2,500 pm	Yes	04-Feb	Yes	one	No	No	No	27	No	27	No	1.5
20-24	Yes	No	Yes	R501 - R2,500 pm	No	04-Feb	Yes	one	No	No	No	22	No	55	No	1.5
20-24	Yes	Yes	Yes	R501 - R2,500 pm	No	04-Feb	Yes	one	No	Yes	No	29	No	15	No	1.8
20-24	Yes	Yes	Yes	R501 - R2,500 pm	No	04-Feb	Yes	none	.	Yes	No	25	.	20	No	1.6
20-24	Yes	Yes	Yes	R2,501 - R6,000 pm	No	04-Feb	Yes	one	No	Yes	No	25	Yes	10	No	1.8
20-24	No	No	No	R501 - R2,500 pm	Yes	04-Feb	Yes	one	Yes	No	No	28	No	12	No	1.3
20-24	Yes	Yes	Yes	R501 - R2,500 pm	Yes	04-Feb	Yes	one	Yes	No	No	27	No	23	No	2
20-24	Yes	Yes	Yes	greater than R6,000 pm	Yes	04-Feb	No	one	Yes	No	No	25	No	46	No	1.5
15-19	Yes	Yes	Yes	R2,501 - R6,000 pm	No	04-Feb	Yes	one	No	No	No	21	No	52	No	1.4
20-24	Yes	Yes	Yes	R501 - R2,500 pm	Yes	04-Feb	Yes	one	No	No	No	39	No	24	No	1.4
15-19	No	Yes	No	R501 - R2,500 pm	Yes	04-Feb	No	one	No	No	No	23	No	42	No	1.4
20-24	No	Yes	Yes	R0 - R500 pm	Yes	04-Feb	Yes	one	Yes	Yes	No	26	No	30	No	1.4
20-24	Yes	Yes	Yes	R2,501 - R6,000 pm	Yes	04-Feb	Yes	one	No	Yes	No	26	Yes	11	No	1.2
20-24	Yes	Yes	No	R0 - R500 pm	Yes	04-Feb	Yes	one	No	No	No	24	No	56	No	1.8
20-24	Yes	Yes	Yes	R501 - R2,500 pm	No	04-Feb	Yes	none	.	Yes	No	28	.	42	No	1.2
20-24	Yes	Yes	Yes	R0 - R500 pm	Yes	04-Feb	Yes	one	No	No	No	30	No	30	No	1.2
15-19	Yes	Yes	Yes	R501 - R2,500 pm	Yes	04-Feb	No	one	No	No	No	22	No	13	No	1.3
15-19	Yes	Yes	Yes	R501 - R2,500 pm	Yes	04-Feb	Yes	one	No	Yes	No	25	No	51	No	1.2
20-24	No	Yes	Yes	R501 - R2,500 pm	Yes	04-Feb	Yes	none	.	No	No	28	.	14	Yes	0.6
20-24	No	Yes	Yes	R501 - R2,500 pm	Yes	04-Feb	Yes	one	No	Yes	No	33	No	14	No	1.2
20-24	Yes	No	Yes	R0 - R500 pm	No	04-Feb	No	one	No	No	No	29	Yes	17	Yes	0.7
20-24	Yes	No	Yes	R0 - R500 pm	Yes	04-Feb	Yes	one	No	Yes	No	26	Yes	35	No	1.6
20-24	No	No	Yes	R501 - R2,500 pm	Yes	04-Feb	Yes	one	No	Yes	No	34	Yes	17	No	1.2
20-24	No	No	Yes	R501 - R2,500 pm	Yes	04-Feb	Yes	one	No	No	No	28	No	13	No	1.7

20-24	Yes	No	No	R2,501 - R6,000 pm	Yes	04-Feb	No	none	.	No	No	25	.	38	No	1.7
20-24	Yes	No	No	R501 - R2,500 pm	Yes	04-Feb	No	none	.	No	No	25	.	60	No	1.7
15-19	Yes	No	Yes	R501 - R2,500 pm	No	04-Feb	No	one	No	Yes	No	27	No	12	No	1.9
20-24	Yes	No	Yes	R2,501 - R6,000 pm	No	04-Feb	No	none	.	Yes	No	21	.	59	No	1.7
15-19	Yes	No	Yes	R0 - R500 pm	Yes	04-Feb	Yes	one	No	No	No	23	No	27	No	1.7
15-19	Yes	No	Yes	R501 - R2,500 pm	No	04-Feb	No	none	.	No	No	21	.	13	No	1.7
20-24	Yes	No	Yes	R2,501 - R6,000 pm	Yes	04-Feb	Yes	one	No	Yes	No	28	No	56	No	1.7
20-24	No	No	Yes	R0 - R500 pm	No	04-Feb	Yes	one	No	No	No	31	No	14	No	1.7
20-24	Yes	No	Yes	greater than R6,000 pm	Yes	04-Feb	Yes	none	.	.	.	.	.	59	Yes	0.8
20-24	Yes	No	Yes	R501 - R2,500 pm	Yes	04-Feb	Yes	one	No	Yes	No	29	No	29	No	1.7
20-24	Yes	Yes	Yes	R2,501 - R6,000 pm	No	04-Feb	Yes	one	No	Yes	No	27	No	13	No	1.1
15-19	No	Yes	Yes	R2,501 - R6,000 pm	Yes	04-Feb	No	one	No	Yes	No	21	No	21	No	1.4
20-24	Yes	Yes	Yes	R2,501 - R6,000 pm	Yes	04-Feb	Yes	one	No	No	No	31	No	12	No	1.4
20-24	No	Yes	Yes	R0 - R500 pm	No	04-Feb	Yes	one	No	No	No	32	No	17	No	1.1
20-24	Yes	Yes	Yes	R0 - R500 pm	Yes	04-Feb	No	one	No	No	No	24	No	37	No	1.9
20-24	Yes	Yes	Yes	R501 - R2,500 pm	Yes	04-Feb	Yes	one	No	No	No	43	No	37	No	1.3
20-24	No	Yes	Yes	greater than R6,000 pm	Yes	04-Feb	Yes	one	No	Yes	No	21	No	46	No	1.9
20-24	No	Yes	No	R501 - R2,500 pm	No	04-Feb	Yes	one	No	No	No	40	No	34	No	1.4
20-24	Yes	Yes	Yes	greater than R6,000 pm	Yes	04-Feb	Yes	none	.	Yes	No	23	.	23	No	1.4
15-19	No	Yes	Yes	R501 - R2,500 pm	Yes	04-Feb	No	one	No	Yes	No	23	No	43	No	1.4
20-24	Yes	Yes	Yes	R501 - R2,500 pm	Yes	04-Feb	No	one	No	Yes	No	29	No	16	No	1.4
15-19	No	Yes	Yes	greater than R6,000 pm	Yes	04-Feb	Yes	none	.	.	No	19	.	28	No	1.4
15-19	Yes	Yes	Yes	R2,501 - R6,000 pm	Yes	04-Feb	No	one	No	Yes	No	24	Yes	33	No	1.2
20-24	Yes	Yes	Yes	R2,501 - R6,000 pm	No	04-Feb	No	2 or more	No	Yes	No	24	Yes	12	No	1.2
15-19	No	Yes	Yes	R501 - R2,500 pm	Yes	04-Feb	Yes	one	No	No	No	22	No	42	No	1.1
15-19	Yes	Yes	Yes	greater than R6,000 pm	Yes	04-Feb	Yes	one	No	No	No	22	No	16	No	1.3
20-24	Yes	Yes	Yes	R2,501 - R6,000 pm	Yes	04-Feb	Yes	one	No	Yes	No	28	No	19	No	1.2
20-24	Yes	Yes	Yes	greater than R6,000 pm	Yes	04-Feb	Yes	one	Yes	Yes	No	34	No	20	No	1.1
20-24	Yes	Yes	Yes	R2,501 - R6,000 pm	Yes	04-Feb	Yes	one	No	No	No	26	No	18	No	1.3
20-24	No	Yes	Yes	R501 - R2,500 pm	Yes	04-Feb	Yes	one	No	Yes	No	26	No	27	No	1.3
15-19	No	Yes	Yes	R501 - R2,500 pm	Yes	04-Feb	No	one	No	.	No	24	No	16	No	1.3
20-24	No	Yes	Yes	R2,501 - R6,000 pm	Yes	04-Feb	Yes	2 or more	No	Yes	No	20	No	59	No	1.3
15-19	No	Yes	Yes	R501 - R2,500 pm	No	04-Feb	No	one	No	No	No	27	No	9	No	1.1
15-19	Yes	Yes	Yes	R501 - R2,500 pm	Yes	04-Feb	Yes	one	No	Yes	No	24	No	26	No	1.1
15-19	No	Yes	Yes	R2,501 - R6,000 pm	No	04-Feb	No	one	No	Yes	No	24	No	24	No	1.3
15-19	No	Yes	Yes	R501 - R2,500 pm	No	04-Feb	No	one	No	Yes	No	23	No	9	No	1.7
20-24	Yes	Yes	Yes	R501 - R2,500 pm	No	04-Feb	No	one	No	No	No	27	No	12	No	1.9
15-19	No	Yes	Yes	R501 - R2,500 pm	Yes	04-Feb	No	one	Yes	.	No	24	No	16	No	1.3
20-24	Yes	Yes	Yes	greater than R6,000 pm	Yes	04-Feb	No	one	Yes	Yes	No	23	No	30	No	1.3

15-19	No	Yes	No	R2,501 - R6,000 pm	Yes	04-Feb	Yes	one	No	Yes	No	24	Yes	9	No	1.2
20-24	Yes	Yes	Yes	R501 - R2,500 pm	Yes	04-Feb	Yes	one	Yes	Yes	No	29	No	19	No	1.2
20-24	Yes	Yes	Yes	R2,501 - R6,000 pm	No	04-Feb	No	one	Yes	Yes	No	25	No	27	No	1.2
15-19	No	Yes	Yes	greater than R6,000 pm	Yes	04-Feb	Yes	one	No	Yes	No	23	No	25	No	1.2
15-19	Yes	Yes	Yes	greater than R6,000 pm	Yes	04-Feb	No	one	No	Yes	No	26	No	32	No	1.5
20-24	Yes	Yes	Yes	greater than R6,000 pm	Yes	04-Feb	Yes	one	No	Yes	No	25	No	59	No	1.5
15-19	No	Yes	Yes	R501 - R2,500 pm	No	04-Feb	No	one	No	Yes	No	19	No	11	No	1.5
20-24	Yes	Yes	No	R0 - R500 pm	Yes	04-Feb	Yes	one	Yes	Yes	No	23	Yes	22	No	1.4
20-24	Yes	Yes	Yes	greater than R6,000 pm	No	04-Feb	No	one	Yes	Yes	No	26	Yes	52	No	1.3
20-24	No	Yes	Yes	R501 - R2,500 pm	No	04-Feb	Yes	one	No	No	No	23	No	11	No	1.4
15-19	No	Yes	Yes	R0 - R500 pm	Yes	04-Feb	Yes	none	.	Yes	No	20	.	42	No	1.6
20-24	Yes	Yes	Yes	R501 - R2,500 pm	Yes	04-Feb	Yes	one	No	Yes	No	27	No	23	No	1.5
15-19	No	Yes	Yes	R501 - R2,500 pm	Yes	04-Feb	Yes	none	.	Yes	No	21	.	16	No	1.5
20-24	No	Yes	Yes	R501 - R2,500 pm	Yes	04-Feb	Yes	one	No	No	No	22	No	30	No	1.8
15-19	No	Yes	Yes	R501 - R2,500 pm	No	04-Feb	Yes	one	No	Yes	No	25	No	11	Yes	0.9
15-19	No	No	Yes	R501 - R2,500 pm	Yes	04-Feb	Yes	2 or more	No	Yes	No	26	No	29	No	1.2
15-19	No	No	Yes	R0 - R500 pm	Yes	04-Feb	Yes	one	No	No	No	25	No	20	No	1.2
15-19	No	No	Yes	R2,501 - R6,000 pm	No	04-Feb	No	one	No	No	No	19	No	29	No	1.2
15-19	No	No	Yes	R2,501 - R6,000 pm	Yes	04-Feb	Yes	one	No	Yes	No	23	No	21	No	1.3
20-24	No	No	No	R501 - R2,500 pm	Yes	04-Feb	Yes	one	Yes	Yes	No	25	Yes	24	No	1.7
20-24	No	No	Yes	R501 - R2,500 pm	No	04-Feb	Yes	one	Yes	No	No	30	No	13	No	1.7
20-24	No	No	Yes	R501 - R2,500 pm	Yes	04-Feb	Yes	one	No	Yes	No	34	No	48	Yes	0.9
20-24	No	No	Yes	R501 - R2,500 pm	No	04-Feb	Yes	one	No	No	No	24	No	33	No	1.2
20-24	Yes	No	Yes	R501 - R2,500 pm	Yes	04-Feb	No	2 or more	No	No	No	29	No	25	No	1.2
20-24	No	No	No	R501 - R2,500 pm	Yes	04-Feb	Yes	one	No	No	No	25	No	35	No	1.2
20-24	Yes	No	Yes	R2,501 - R6,000 pm	No	04-Feb	Yes	one	Yes	Yes	No	31	No	35	No	1.3
20-24	Yes	Yes	Yes	R501 - R2,500 pm	Yes	04-Feb	Yes	one	No	Yes	No	25	No	10	No	1
20-24	No	No	Yes	R2,501 - R6,000 pm	Yes	04-Feb	Yes	one	No	No	No	27	No	43	No	1.2
20-24	Yes	No	Yes	R501 - R2,500 pm	Yes	04-Feb	Yes	one	No	Yes	No	31	No	22	No	1.2
20-24	Yes	No	No	R501 - R2,500 pm	Yes	04-Feb	No	one	No	No	No	29	No	59	No	1.2
20-24	Yes	No	No	R2,501 - R6,000 pm	No	04-Feb	Yes	one	No	No	No	25	No	22	No	1.4
20-24	Yes	No	Yes	R2,501 - R6,000 pm	Yes	04-Feb	Yes	one	No	No	No	22	No	33	No	1.2
20-24	No	No	No	R0 - R500 pm	No	04-Feb	Yes	none	.	Yes	No	27	.	12	No	1.2
20-24	No	No	Yes	R2,501 - R6,000 pm	Yes	04-Feb	Yes	one	No	No	No	29	No	22	No	1.2
20-24	Yes	No	Yes	R2,501 - R6,000 pm	Yes	04-Feb	Yes	one	No	Yes	No	30	No	17	No	1.9
20-24	No	No	Yes	R2,501 - R6,000 pm	Yes	04-Feb	Yes	one	No	Yes	No	21	No	16	No	1.6
20-24	Yes	No	Yes	greater than R6,000 pm	Yes	04-Feb	Yes	none	.	Yes	No	22	.	17	No	1.6
15-19	Yes	No	Yes	R2,501 - R6,000 pm	Yes	04-Feb	Yes	2 or more	No	Yes	Yes	21	No	30	No	1.5
20-24	Yes	No	No	R501 - R2,500 pm	No	04-Feb	Yes	2 or more	No	No	No	26	No	59	No	1.2
20-24	Yes	No	Yes	R501 - R2,500 pm	Yes	04-Feb	Yes	one	Yes	No	No	30	No	17	No	1.3
20-24	Yes	No	Yes	R2,501 - R6,000 pm	Yes	04-Feb	Yes	one	No	Yes	No	25	No	60	No	1.6

15-19	No	No	Yes	R2,501 - R6,000 pm	No	04-Feb	Yes	2 or more	No	No	No	26	No	53	No	1.7
20-24	Yes	No	Yes	R501 - R2,500 pm	Yes	04-Feb	Yes	one	No	No	No	32	No	22	No	1.6
20-24	Yes	No	Yes	R2,501 - R6,000 pm	Yes	04-Feb	Yes	none	.	No	No	30	.	39	No	1.5
20-24	No	No	Yes	R501 - R2,500 pm	No	04-Feb	No	one	No	No	No	34	Yes	29	No	1.4
20-24	Yes	No	Yes	R501 - R2,500 pm	Yes	04-Feb	No	none	.	Yes	No	25	.	15	No	1.5
15-19	No	No	Yes	R501 - R2,500 pm	Yes	04-Feb	No	one	No	Yes	No	27	Yes	27	No	1.4
20-24	Yes	No	Yes	R501 - R2,500 pm	Yes	04-Feb	Yes	one	No	Yes	No	24	No	44	No	1.2
20-24	Yes	No	Yes	R501 - R2,500 pm	Yes	04-Feb	Yes	one	No	Yes	No	23	No	24	No	1.5
20-24	No	No	Yes	R501 - R2,500 pm	No	04-Feb	Yes	none	.	.	No	22	.	50	No	1.1
20-24	No	No	No	R501 - R2,500 pm	Yes	04-Feb	Yes	one	No	No	No	27	No	13	Yes	0.6
20-24	Yes	No	Yes	R501 - R2,500 pm	No	04-Feb	Yes	none	.	Yes	No	22	.	26	No	1.2
20-24	Yes	No	Yes	R2,501 - R6,000 pm	Yes	04-Feb	No	one	No	Yes	No	29	No	44	No	1.3
20-24	Yes	No	Yes	R2,501 - R6,000 pm	No	04-Feb	Yes	one	No	No	No	24	Yes	41	No	1.2
20-24	No	Yes	Yes	R2,501 - R6,000 pm	Yes	04-Feb	No	none	.	.	.	.	.	44	No	1.1
20-24	Yes	Yes	Yes	greater than R6,000 pm	No	04-Feb	Yes	one	No	Yes	No	33	No	29	No	1.1
20-24	Yes	Yes	Yes	R501 - R2,500 pm	No	04-Feb	No	one	No	Yes	No	27	No	15	No	1.1
20-24	No	Yes	Yes	R501 - R2,500 pm	Yes	04-Feb	Yes	one	No	No	No	28	No	10	No	1.1
20-24	Yes	Yes	Yes	greater than R6,000 pm	Yes	04-Feb	Yes	none	.	Yes	No	27	.	18	No	1.1
15-19	No	Yes	Yes	greater than R6,000 pm	Yes	04-Feb	Yes	one	Yes	No	No	28	No	17	Yes	0.5
20-24	Yes	Yes	No	R2,501 - R6,000 pm	Yes	04-Feb	Yes	one	No	No	No	27	No	22	Yes	0.7
20-24	Yes	Yes	Yes	R0 - R500 pm	Yes	04-Feb	No	one	No	Yes	No	23	No	22	No	1.4
15-19	Yes	Yes	Yes	R501 - R2,500 pm	Yes	04-Feb	Yes	2 or more	.	Yes	No	20	No	27	No	1.2
15-19	No	Yes	Yes	R501 - R2,500 pm	No	04-Feb	No	none	.	.	.	.	.	26	No	1.3
20-24	No	Yes	Yes	R501 - R2,500 pm	Yes	04-Feb	Yes	one	Yes	Yes	Yes	21	No	27	No	1.3
20-24	Yes	Yes	No	R501 - R2,500 pm	Yes	04-Feb	Yes	one	Yes	Yes	No	28	No	28	No	1.3
15-19	Yes	Yes	Yes	R501 - R2,500 pm	Yes	04-Feb	No	one	No	Yes	No	21	No	18	No	1.3
20-24	Yes	Yes	Yes	R2,501 - R6,000 pm	Yes	04-Feb	Yes	one	No	Yes	No	29	Yes	10	No	1.4
15-19	No	Yes	Yes	R2,501 - R6,000 pm	No	04-Feb	Yes	one	No	No	No	24	No	17	No	1.1
20-24	No	Yes	Yes	R2,501 - R6,000 pm	Yes	04-Feb	Yes	one	No	Yes	No	27	Yes	20	Yes	0.9
15-19	Yes	Yes	Yes	R501 - R2,500 pm	Yes	04-Feb	Yes	none	.	.	.	.	.	53	No	1.3
20-24	Yes	Yes	Yes	greater than R6,000 pm	Yes	04-Feb	Yes	one	No	No	No	25	No	42	Yes	0.7
15-19	Yes	Yes	No	R2,501 - R6,000 pm	Yes	04-Feb	Yes	one	Yes	Yes	No	23	No	38	Yes	0.9
20-24	Yes	Yes	No	R2,501 - R6,000 pm	Yes	04-Feb	No	one	No	Yes	No	27	No	10	No	1.4
20-24	Yes	Yes	No	R2,501 - R6,000 pm	Yes	04-Feb	Yes	one	No	Yes	No	29	Yes	18	No	1.4
20-24	No	Yes	Yes	R501 - R2,500 pm	Yes	04-Feb	Yes	one	No	No	No	27	No	25	No	1.4
20-24	Yes	Yes	Yes	R501 - R2,500 pm	No	04-Feb	No	one	No	Yes	No	22	Yes	24	No	1.3
20-24	Yes	Yes	Yes	R2,501 - R6,000 pm	Yes	04-Feb	Yes	one	No	Yes	No	25	Yes	36	No	1.7
20-24	Yes	Yes	Yes	R501 - R2,500 pm	Yes	04-Feb	Yes	one	Yes	Yes	No	34	No	32	No	1.6
15-19	No	Yes	Yes	R2,501 - R6,000 pm	Yes	04-Feb	No	none	.	.	.	.	.	47	No	1.6
15-19	No	Yes	Yes	R501 - R2,500 pm	No	04-Feb	No	one	No	Yes	No	24	No	38	No	1.8
20-24	Yes	Yes	Yes	greater than R6,000 pm	Yes	04-Feb	Yes	one	No	Yes	No	37	No	52	No	1.6

20-24	Yes	Yes	Yes	R2,501 - R6,000 pm	Yes	04-Feb	Yes	one	No	Yes	No	28	No	32	No	1.9
20-24	Yes	Yes	Yes	R501 - R2,500 pm	No	04-Feb	No	one	No	Yes	No	26	No	49	No	1.4
20-24	Yes	Yes	Yes	greater than R6,000 pm	Yes	04-Feb	Yes	one	No	Yes	No	24	No	21	No	1.4
20-24	Yes	Yes	Yes	greater than R6,000 pm	Yes	04-Feb	Yes	one	Yes	Yes	No	27	No	36	No	1.3
20-24	Yes	Yes	No	R2,501 - R6,000 pm	Yes	04-Feb	Yes	one	No	Yes	No	28	No	36	No	1.1
20-24	Yes	Yes	Yes	greater than R6,000 pm	Yes	04-Feb	Yes	one	Yes	Yes	No	29	No	17	No	1.1
20-24	Yes	Yes	Yes	greater than R6,000 pm	Yes	04-Feb	Yes	one	No	Yes	No	25	No	36	No	1.1
15-19	Yes	Yes	Yes	greater than R6,000 pm	No	04-Feb	No	one	No	No	No	26	Yes	32	No	1.4
15-19	Yes	Yes	Yes	R2,501 - R6,000 pm	No	04-Feb	No	one	No	Yes	No	23	No	53	No	1.4
15-19	No	No	No	R501 - R2,500 pm	Yes	04-Feb	Yes	one	No	No	No	25	No	19	No	1.5
15-19	No	No	Yes	R501 - R2,500 pm	Yes	04-Feb	No	one	Yes	No	No	21	No	28	No	1.4
15-19	No	No	Yes	R2,501 - R6,000 pm	Yes	04-Feb	Yes	one	No	Yes	No	24	No	18	No	1.4
20-24	Yes	Yes	Yes	R501 - R2,500 pm	Yes	04-Feb	Yes	one	Yes	Yes	No	30	Yes	13	No	1.8
15-19	No	Yes	Yes	R501 - R2,500 pm	No	04-Feb	No	one	Yes	Yes	No	28	Yes	23	No	1.8
15-19	No	Yes	Yes	R501 - R2,500 pm	No	04-Feb	Yes	one	Yes	Yes	No	21	No	10	No	1.2
20-24	No	Yes	Yes	R501 - R2,500 pm	Yes	04-Feb	Yes	one	Yes	Yes	No	27	No	22	No	1.2
15-19	Yes	Yes	Yes	R501 - R2,500 pm	Yes	04-Feb	Yes	one	No	Yes	No	24	No	9	No	1.2
20-24	Yes	Yes	Yes	R501 - R2,500 pm	Yes	04-Feb	Yes	one	No	Yes	No	25	No	10	No	1.2
20-24	Yes	Yes	Yes	R501 - R2,500 pm	Yes	04-Feb	No	one	Yes	Yes	No	27	No	13	No	1.3
20-24	Yes	Yes	Yes	R0 - R500 pm	Yes	04-Feb	No	one	Yes	Yes	No	28	No	15	No	1.3
20-24	No	Yes	Yes	R2,501 - R6,000 pm	Yes	04-Feb	Yes	one	No	Yes	No	23	No	25	No	1.3
20-24	No	Yes	Yes	R501 - R2,500 pm	Yes	04-Feb	Yes	one	No	Yes	No	39	No	22	No	1.3
15-19	No	Yes	Yes	R501 - R2,500 pm	Yes	04-Feb	Yes	one	No	No	No	18	No	19	No	1.3
20-24	Yes	Yes	Yes	R501 - R2,500 pm	Yes	04-Feb	No	one	Yes	No	No	25	No	27	No	1.3
20-24	No	Yes	Yes	R501 - R2,500 pm	Yes	04-Feb	Yes	none	.	.	.	.	.	10	No	1.1
20-24	No	Yes	Yes	R501 - R2,500 pm	Yes	04-Feb	No	2 or more	.	Yes	Yes	26	No	27	No	1.4
15-19	No	Yes	Yes	R501 - R2,500 pm	Yes	04-Feb	Yes	one	No	No	No	29	No	9	No	1.4
20-24	Yes	Yes	Yes	R501 - R2,500 pm	Yes	04-Feb	Yes	one	Yes	No	No	29	No	10	Yes	0.7
20-24	Yes	Yes	Yes	R501 - R2,500 pm	No	04-Feb	Yes	one	Yes	Yes	No	28	No	11	No	1.2
20-24	Yes	Yes	Yes	R0 - R500 pm	No	04-Feb	Yes	one	No	No	No	29	No	13	No	1.1
20-24	No	Yes	Yes	R501 - R2,500 pm	Yes	04-Feb	Yes	one	Yes	Yes	No	21	No	20	No	1.1
20-24	Yes	Yes	Yes	R501 - R2,500 pm	No	04-Feb	Yes	one	No	Yes	No	28	No	22	No	1.2
15-19	No	Yes	Yes	greater than R6,000 pm	Yes	04-Feb	Yes	one	No	Yes	No	25	No	28	No	1.3
20-24	Yes	Yes	Yes	R501 - R2,500 pm	No	04-Feb	Yes	one	No	No	No	29	Yes	13	No	1
20-24	Yes	Yes	No	R2,501 - R6,000 pm	Yes	04-Feb	Yes	one	Yes	Yes	Yes	26	No	18	No	1.3
20-24	Yes	Yes	No	R501 - R2,500 pm	No	04-Feb	Yes	one	No	Yes	No	29	No	12	Yes	0.6
15-19	No	Yes	Yes	R501 - R2,500 pm	No	04-Feb	Yes	one	Yes	Yes	Yes	24	No	30	No	1.3
20-24	No	Yes	Yes	R501 - R2,500 pm	No	04-Feb	No	one	No	Yes	No	27	No	10	No	1.2
20-24	Yes	Yes	Yes	greater than R6,000 pm	No	04-Feb	Yes	one	Yes	Yes	No	26	No	17	No	1.3

20-24	Yes	Yes	Yes	R2,501 - R6,000 pm	Yes	04-Feb	Yes	one	No	No	No	28	No	32	No	1.5
20-24	Yes	Yes	Yes	R2,501 - R6,000 pm	Yes	04-Feb	Yes	one	Yes	.	No	23	No	45	No	1.4
20-24	Yes	Yes	No	R501 - R2,500 pm	No	04-Feb	Yes	none	.	No	No	24	.	12	No	1.5
15-19	No	Yes	Yes	R501 - R2,500 pm	No	04-Feb	No	one	No	No	No	21	Yes	20	No	1.5
20-24	Yes	Yes	Yes	R2,501 - R6,000 pm	No	04-Feb	Yes	one	No	Yes	No	25	No	12	No	1.2
20-24	Yes	Yes	Yes	R2,501 - R6,000 pm	No	04-Feb	Yes	one	No	Yes	No	29	No	46	No	1.6
15-19	No	Yes	Yes	greater than R6,000 pm	No	04-Feb	Yes	one	No	No	No	20	No	28	No	1.2
20-24	No	Yes	Yes	R2,501 - R6,000 pm	Yes	04-Feb	Yes	2 or more	No	No	No	30	No	31	No	1.3
20-24	No	Yes	Yes	R501 - R2,500 pm	Yes	04-Feb	Yes	one	Yes	No	No	23	No	59	No	1
15-19	Yes	Yes	Yes	greater than R6,000 pm	Yes	04-Feb	No	one	No	Yes	No	24	No	53	No	1.1
20-24	Yes	Yes	Yes	greater than R6,000 pm	Yes	04-Feb	Yes	one	No	Yes	No	22	No	30	No	1.6
20-24	Yes	Yes	Yes	R501 - R2,500 pm	Yes	04-Feb	No	one	Yes	Yes	No	23	No	11	No	1.3
20-24	Yes	Yes	Yes	R501 - R2,500 pm	Yes	04-Feb	No	one	Yes	No	No	31	No	21	No	1.7
20-24	Yes	Yes	Yes	R2,501 - R6,000 pm	Yes	04-Feb	Yes	one	No	.	No	29	No	21	No	1.4
20-24	Yes	Yes	Yes	R2,501 - R6,000 pm	No	04-Feb	Yes	one	No	Yes	No	32	No	21	No	1.5
20-24	Yes	Yes	Yes	R2,501 - R6,000 pm	Yes	04-Feb	No	one	No	No	No	25	Yes	29	No	1.5
20-24	Yes	Yes	Yes	R2,501 - R6,000 pm	Yes	04-Feb	No	one	No	No	No	24	No	11	No	1.4
20-24	Yes	Yes	Yes	R501 - R2,500 pm	No	04-Feb	No	2 or more	No	Yes	No	25	No	22	No	1.4
20-24	Yes	Yes	Yes	greater than R6,000 pm	Yes	04-Feb	Yes	one	No	Yes	No	38	No	43	No	1.5
15-19	Yes	Yes	Yes	greater than R6,000 pm	Yes	04-Feb	No	one	No	Yes	No	25	No	23	No	1.5
15-19	Yes	Yes	No	R501 - R2,500 pm	No	04-Feb	No	one	No	Yes	No	20	No	32	No	1.5
20-24	Yes	No	Yes	R2,501 - R6,000 pm	Yes	04-Feb	Yes	one	No	No	No	28	No	20	Yes	0.8
20-24	Yes	No	Yes	R501 - R2,500 pm	Yes	04-Feb	Yes	one	Yes	No	No	27	No	21	No	1.9
15-19	Yes	No	Yes	R501 - R2,500 pm	No	04-Feb	Yes	one	No	Yes	No	20	No	9	No	1.7
20-24	Yes	No	Yes	R2,501 - R6,000 pm	Yes	04-Feb	Yes	one	No	Yes	No	27	No	40	No	1.7
15-19	No	No	Yes	R2,501 - R6,000 pm	No	04-Feb	No	2 or more	No	Yes	No	25	No	18	No	2
20-24	Yes	Yes	Yes	R501 - R2,500 pm	No	04-Feb	Yes	one	Yes	Yes	No	26	No	40	No	1.4
15-19	No	Yes	No	R0 - R500 pm	Yes	04-Feb	Yes	none	.	No	No	26	.	15	No	1.1
20-24	No	Yes	No	R501 - R2,500 pm	Yes	04-Feb	Yes	one	No	No	No	30	No	33	No	1.1
20-24	Yes	Yes	Yes	R2,501 - R6,000 pm	Yes	04-Feb	Yes	one	No	No	No	24	No	18	No	1.4
15-19	No	Yes	No	R0 - R500 pm	Yes	04-Feb	Yes	one	No	No	No	22	No	10	No	1.8
20-24	Yes	Yes	Yes	R2,501 - R6,000 pm	Yes	04-Feb	Yes	one	No	Yes	No	29	No	10	Yes	0.7
20-24	No	Yes	Yes	R501 - R2,500 pm	No	04-Feb	No	one	No	Yes	No	24	No	10	No	1.3
20-24	Yes	Yes	Yes	R2,501 - R6,000 pm	No	04-Feb	No	one	No	Yes	No	28	No	25	No	1.4
20-24	No	Yes	Yes	R501 - R2,500 pm	Yes	04-Feb	Yes	one	No	No	No	25	No	47	No	1.4
20-24	No	Yes	No	R0 - R500 pm	Yes	04-Feb	Yes	one	No	No	No	23	No	12	No	1.4
20-24	Yes	Yes	Yes	R0 - R500 pm	Yes	04-Feb	No	one	No	No	No	32	Yes	15	No	1.4
20-24	Yes	Yes	Yes	R2,501 - R6,000 pm	Yes	04-Feb	Yes	none	.	.	.	.	.	22	No	1.3
15-19	Yes	Yes	Yes	R2,501 - R6,000 pm	No	04-Feb	No	one	No	Yes	No	25	No	30	No	1.3
15-19	No	Yes	No	R2,501 - R6,000 pm	Yes	04-Feb	Yes	none	.	Yes	No	19	.	30	No	1.3
20-24	Yes	Yes	Yes	R2,501 - R6,000 pm	No	04-Feb	Yes	one	No	.	No	26	No	11	No	1.3

20-24	Yes	Yes	Yes	R2,501 - R6,000 pm	No	04-Feb	No	one	No	Yes	No	23	No	32	No	1.3
20-24	Yes	Yes	Yes	R501 - R2,500 pm	Yes	04-Feb	Yes	one	No	Yes	No	29	No	60	No	1.2
20-24	Yes	Yes	Yes	R501 - R2,500 pm	Yes	04-Feb	Yes	one	No	Yes	No	33	Yes	40	No	1
20-24	Yes	Yes	Yes	R2,501 - R6,000 pm	Yes	04-Feb	Yes	one	No	No	No	26	No	21	No	1.1
20-24	No	Yes	Yes	R2,501 - R6,000 pm	No	04-Feb	Yes	one	No	No	No	34	No	43	No	1
15-19	No	Yes	Yes	R501 - R2,500 pm	No	04-Feb	No	none	.	Yes	No	20	.	52	No	1.4
20-24	Yes	Yes	Yes	R501 - R2,500 pm	No	04-Feb	Yes	one	No	No	No	24	No	14	No	1.4
20-24	Yes	Yes	No	R501 - R2,500 pm	Yes	04-Feb	Yes	one	No	No	No	27	No	17	No	1.1
20-24	Yes	Yes	Yes	R2,501 - R6,000 pm	No	04-Feb	No	one	No	Yes	No	26	No	9	No	1.1
20-24	Yes	Yes	Yes	R501 - R2,500 pm	Yes	04-Feb	Yes	one	No	No	No	27	No	34	No	1.1
15-19	No	Yes	Yes	R2,501 - R6,000 pm	No	04-Feb	No	one	Yes	Yes	No	22	No	28	No	1.4
20-24	No	Yes	Yes	R501 - R2,500 pm	No	04-Feb	Yes	none	.	Yes	No	24	.	20	No	1.4
15-19	No	Yes	Yes	R2,501 - R6,000 pm	Yes	04-Feb	Yes	one	No	No	No	21	No	35	No	1.4
20-24	No	Yes	Yes	R501 - R2,500 pm	Yes	04-Feb	Yes	one	No	No	No	36	No	19	No	1.3
20-24	Yes	No	Yes	greater than R6,000 pm	Yes	04-Feb	Yes	none	.	Yes	No	28	.	11	No	1.1
15-19	No	No	Yes	R2,501 - R6,000 pm	Yes	04-Feb	Yes	one	No	Yes	No	19	Yes	9	No	1.1
20-24	No	No	Yes	greater than R6,000 pm	Yes	04-Feb	Yes	one	No	Yes	No	28	Yes	40	No	1.4
20-24	No	No	Yes	R501 - R2,500 pm	Yes	04-Feb	Yes	one	No	Yes	No	26	No	16	Yes	0.8
20-24	Yes	No	Yes	R501 - R2,500 pm	Yes	04-Feb	Yes	one	No	Yes	No	24	No	30	No	1.6
15-19	No	No	Yes	R501 - R2,500 pm	Yes	04-Feb	Yes	one	No	No	No	28	No	14	No	1.6
20-24	No	No	No	R501 - R2,500 pm	Yes	04-Feb	Yes	one	No	Yes	No	32	No	33	No	2.1
20-24	Yes	No	Yes	R2,501 - R6,000 pm	Yes	04-Feb	No	none	.	Yes	No	25	.	56	No	1.7
20-24	Yes	Yes	Yes	R2,501 - R6,000 pm	No	04-Feb	Yes	one	No	Yes	No	28	Yes	14	No	1.6
20-24	No	Yes	Yes	R501 - R2,500 pm	No	04-Feb	No	one	Yes	Yes	No	27	No	22	No	1.5
15-19	No	Yes	Yes	R2,501 - R6,000 pm	Yes	04-Feb	No	one	Yes	No	Yes	25	No	40	Yes	1
15-19	No	Yes	Yes	greater than R6,000 pm	Yes	04-Feb	Yes	one	No	Yes	No	28	No	32	No	1.9
20-24	Yes	Yes	No	greater than R6,000 pm	Yes	04-Feb	No	none	.	Yes	No	23	.	22	No	1.9
20-24	Yes	Yes	Yes	R2,501 - R6,000 pm	No	04-Feb	No	one	No	No	No	35	No	24	No	1.5
20-24	No	Yes	Yes	R501 - R2,500 pm	Yes	04-Feb	Yes	one	Yes	No	No	30	No	22	No	1.2
20-24	Yes	No	Yes	R2,501 - R6,000 pm	Yes	04-Feb	Yes	2 or more	No	Yes	No	30	No	31	No	1.9
20-24	No	No	No	R501 - R2,500 pm	Yes	04-Feb	Yes	one	No	Yes	No	25	No	16	No	1.7
20-24	No	No	Yes	R501 - R2,500 pm	Yes	04-Feb	Yes	one	No	Yes	No	31	No	36	No	1.4
20-24	No	No	Yes	R501 - R2,500 pm	Yes	04-Feb	No	one	No	Yes	No	25	No	36	No	1.4
20-24	Yes	No	Yes	R2,501 - R6,000 pm	Yes	04-Feb	Yes	one	No	Yes	No	26	No	52	No	1.4
20-24	Yes	No	Yes	R2,501 - R6,000 pm	Yes	04-Feb	Yes	one	Yes	Yes	No	21	No	32	No	1.1
20-24	Yes	No	Yes	R0 - R500 pm	Yes	04-Feb	Yes	none	.	No	No	31	.	16	Yes	0.7
20-24	Yes	No	Yes	R2,501 - R6,000 pm	Yes	04-Feb	Yes	one	No	Yes	No	27	No	20	No	1.4
20-24	Yes	No	Yes	R2,501 - R6,000 pm	Yes	04-Feb	Yes	one	No	Yes	No	26	No	19	No	1.2
15-19	Yes	No	Yes	R501 - R2,500 pm	Yes	04-Feb	No	2 or more	No	No	No	19	No	26	No	1.2
20-24	Yes	No	Yes	R2,501 - R6,000 pm	No	04-Feb	No	none	.	No	No	20	.	31	No	1.2
20-24	Yes	No	Yes	R501 - R2,500 pm	No	04-Feb	Yes	one	Yes	No	No	27	No	53	No	1.6
15-19	No	No	Yes	R2,501 - R6,000 pm	Yes	04-Feb	Yes	one	No	Yes	No	29	No	37	No	1.6

20-24	Yes	No	Yes	R2,501 - R6,000 pm	Yes	04-Feb	Yes	one	Yes	Yes	No	27	No	49	No	1.8
15-19	No	No	Yes	R2,501 - R6,000 pm	Yes	04-Feb	Yes	one	No	Yes	No	18	No	30	No	1.1
20-24	Yes	No	Yes	R501 - R2,500 pm	Yes	04-Feb	Yes	one	No	No	No	27	No	20	No	1.4
15-19	No	No	No	R501 - R2,500 pm	Yes	04-Feb	Yes	one	No	Yes	No	21	No	27	No	1.2
20-24	No	No	Yes	R501 - R2,500 pm	Yes	04-Feb	Yes	one	No	No	No	27	No	29	No	1.3
20-24	Yes	No	Yes	R501 - R2,500 pm	No	04-Feb	Yes	one	Yes	Yes	No	26	No	15	No	1.3
20-24	Yes	No	No	R501 - R2,500 pm	Yes	04-Feb	No	one	No	No	No	30	No	29	No	1.2
20-24	Yes	No	Yes	R2,501 - R6,000 pm	Yes	04-Feb	Yes	one	No	No	No	30	No	43	No	1.4
20-24	Yes	No	No	R501 - R2,500 pm	Yes	04-Feb	Yes	one	No	Yes	No	25	No	29	No	1.4
15-19	No	No	Yes	R501 - R2,500 pm	No	04-Feb	Yes	none	.	Yes	No	22	.	32	No	1.7
20-24	Yes	No	Yes	greater than R6,000 pm	Yes	5 or more	Yes	one	No	Yes	No	26	No	11	No	1.3
20-24	Yes	Yes	Yes	.	Yes	5 or more	Yes	one	Yes	Yes	No	27	Yes	9	No	1.8
20-24	No	No	No	R501 - R2,500 pm	Yes	5 or more	Yes	one	Yes	No	No	31	Yes	11	No	1.2
20-24	Yes	Yes	Yes	R501 - R2,500 pm	Yes	5 or more	Yes	one	No	Yes	No	29	No	7	No	1.3
20-24	Yes	Yes	Yes	greater than R6,000 pm	Yes	5 or more	Yes	one	No	No	No	27	No	48	No	1.5
15-19	Yes	Yes	Yes	R501 - R2,500 pm	No	5 or more	Yes	one	No	No	No	26	Yes	38	No	1.4
20-24	Yes	Yes	Yes	R501 - R2,500 pm	Yes	5 or more	No	one	No	Yes	Yes	32	No	10	No	1.6
15-19	No	Yes	Yes	R501 - R2,500 pm	Yes	5 or more	Yes	one	No	No	No	24	No	11	No	1.6
20-24	No	Yes	Yes	R0 - R500 pm	Yes	5 or more	Yes	2 or more	No	Yes	No	29	No	12	Yes	0.6
20-24	Yes	No	Yes	greater than R6,000 pm	No	5 or more	No	one	No	No	No	29	No	24	No	1.4
20-24	No	Yes	No	R501 - R2,500 pm	No	5 or more	.	one	No	.	No	19	No	24	No	1.8
20-24	No	No	Yes	R501 - R2,500 pm	Yes	5 or more	Yes	one	No	No	No	25	No	23	No	1.8
15-19	No	No	Yes	R2,501 - R6,000 pm	Yes	5 or more	No	one	No	Yes	No	28	Yes	80	No	1.8
15-19	No	No	Yes	R2,501 - R6,000 pm	No	5 or more	No	one	No	.	No	20	No	14	Yes	1
20-24	Yes	No	Yes	R501 - R2,500 pm	Yes	5 or more	Yes	2 or more	No	No	Yes	37	No	17	Yes	1.1
20-24	Yes	Yes	No	R0 - R500 pm	Yes	5 or more	Yes	one	No	Yes	No	26	No	11	No	2.3
20-24	No	Yes	No	R0 - R500 pm	No	5 or more	Yes	one	No	No	No	32	No	60	No	2
20-24	No	Yes	No	R0 - R500 pm	No	5 or more	Yes	one	No	Yes	No	35	No	11	No	2.4
20-24	No	Yes	Yes	R501 - R2,500 pm	No	5 or more	Yes	one	Yes	No	No	30	No	7	No	1.9
20-24	Yes	No	Yes	.	Yes	5 or more	Yes	one	No	No	No	30	No	55	No	1.4
20-24	Yes	Yes	Yes	.	No	5 or more	Yes	none	.	.	.	.	.	8	No	1.9
20-24	Yes	Yes	Yes	R2,501 - R6,000 pm	Yes	5 or more	No	2 or more	No	Yes	No	30	No	29	No	1.9
15-19	No	No	Yes	R0 - R500 pm	Yes	5 or more	No	one	No	Yes	No	27	Yes	25	No	1.8
20-24	No	Yes	Yes	R501 - R2,500 pm	No	5 or more	No	one	No	Yes	No	28	No	14	No	1.6
15-19	No	No	Yes	R501 - R2,500 pm	No	5 or more	Yes	one	No	Yes	No	27	No	24	Yes	0.9
20-24	No	Yes	Yes	R501 - R2,500 pm	Yes	5 or more	Yes	one	No	Yes	No	26	No	14	No	1.6
20-24	Yes	No	Yes	R0 - R500 pm	No	5 or more	No	one	Yes	No	No	27	No	28	No	1.8
15-19	Yes	No	Yes	R501 - R2,500 pm	Yes	5 or more	Yes	one	Yes	Yes	No	30	No	18	No	1.7
20-24	Yes	Yes	Yes	R0 - R500 pm	No	5 or more	Yes	one	No	No	No	26	No	10	No	1.9
15-19	No	Yes	Yes	.	No	5 or more	Yes	one	No	No	No	24	No	15	Yes	0.8
20-24	Yes	Yes	Yes	.	No	5 or more	Yes	2 or more	No	No	No	38	No	16	No	2.1
20-24	Yes	Yes	Yes	R0 - R500 pm	Yes	5 or more	Yes	one	No	Yes	Yes	37	No	10	No	1.1
20-24	No	Yes	Yes	R501 - R2,500 pm	No	5 or more	Yes	one	No	No	No	24	No	11	No	1.6

20-24	Yes	Yes	Yes	R0 - R500 pm	Yes	5 or more	Yes	2 or more	No	No	No	24	No	13	No	1.7
20-24	No	Yes	Yes	R0 - R500 pm	Yes	5 or more	Yes	one	No	No	No	26	No	11	No	1.6
20-24	Yes	No	Yes	greater than R6,000 pm	Yes	5 or more	Yes	one	No	.	No	23	No	9	No	1.2
15-19	No	Yes	Yes	R2,501 - R6,000 pm	Yes	5 or more	Yes	none	.	.	No	23	.	11	No	2
20-24	Yes	No	Yes	R501 - R2,500 pm	No	5 or more	No	2 or more	No	Yes	No	28	No	25	No	1.2
15-19	Yes	Yes	Yes	greater than R6,000 pm	Yes	5 or more	Yes	one	No	Yes	No	25	No	21	Yes	0.7
15-19	No	Yes	Yes	R2,501 - R6,000 pm	Yes	5 or more	Yes	one	No	No	No	20	No	26	Yes	0.6
15-19	No	Yes	Yes	R2,501 - R6,000 pm	No	5 or more	Yes	one	No	Yes	No	23	No	17	No	1.2
20-24	Yes	Yes	Yes	R2,501 - R6,000 pm	Yes	5 or more	Yes	one	Yes	Yes	No	32	No	28	No	1.3
20-24	No	No	Yes	greater than R6,000 pm	No	5 or more	Yes	one	No	Yes	Yes	29	No	36	No	1.5
20-24	No	No	Yes	greater than R6,000 pm	Yes	5 or more	Yes	one	No	No	No	23	No	20	No	1.8
15-19	No	No	No	R501 - R2,500 pm	Yes	5 or more	Yes	one	No	Yes	No	28	No	25	No	1.5
20-24	No	Yes	Yes	R501 - R2,500 pm	No	5 or more	Yes	one	No	Yes	No	29	No	34	No	1.9
15-19	Yes	Yes	Yes	R501 - R2,500 pm	Yes	5 or more	No	one	No	Yes	No	30	No	30	No	1.3
20-24	Yes	Yes	Yes	R2,501 - R6,000 pm	Yes	5 or more	Yes	one	No	No	No	29	No	25	No	1.4
20-24	No	Yes	Yes	R2,501 - R6,000 pm	No	5 or more	Yes	2 or more	No	No	No	28	No	13	No	1.4
20-24	Yes	Yes	Yes	R2,501 - R6,000 pm	Yes	5 or more	Yes	none	.	Yes	No	20	.	22	No	1.2
15-19	No	Yes	Yes	R501 - R2,500 pm	Yes	5 or more	No	2 or more	No	Yes	No	27	No	16	No	1.4
20-24	Yes	Yes	Yes	R501 - R2,500 pm	Yes	5 or more	Yes	one	No	No	No	32	No	11	No	1.4
20-24	No	Yes	No	R0 - R500 pm	No	5 or more	Yes	2 or more	.	Yes	Yes	24	No	13	No	1.5
15-19	Yes	Yes	Yes	R501 - R2,500 pm	Yes	5 or more	No	one	No	Yes	No	24	Yes	18	No	1.1
20-24	No	Yes	No	R501 - R2,500 pm	No	5 or more	Yes	one	No	No	Yes	25	No	19	No	1.3
20-24	No	No	Yes	R501 - R2,500 pm	Yes	5 or more	Yes	one	No	Yes	No	33	No	22	No	1.1
20-24	No	Yes	No	R0 - R500 pm	Yes	5 or more	Yes	one	No	No	Yes	44	Yes	15	Yes	0.6
15-19	No	No	Yes	R501 - R2,500 pm	Yes	5 or more	No	one	No	No	No	19	Yes	45	No	1.7
20-24	Yes	No	Yes	R2,501 - R6,000 pm	Yes	5 or more	Yes	one	No	No	No	34	No	38	Yes	0.8
20-24	Yes	Yes	Yes	R2,501 - R6,000 pm	Yes	5 or more	Yes	2 or more	No	Yes	No	27	No	48	No	1.1
20-24	Yes	Yes	No	R501 - R2,500 pm	No	5 or more	Yes	one	No	No	No	29	No	9	No	1.3
20-24	No	Yes	Yes	R2,501 - R6,000 pm	Yes	5 or more	Yes	one	No	No	No	25	No	59	No	1.3
20-24	Yes	Yes	Yes	greater than R6,000 pm	Yes	5 or more	Yes	2 or more	No	Yes	No	26	Yes	10	No	1.3
20-24	Yes	Yes	Yes	greater than R6,000 pm	Yes	5 or more	Yes	2 or more	No	No	No	28	No	11	No	1.4
20-24	No	Yes	Yes	R501 - R2,500 pm	No	5 or more	Yes	one	Yes	No	Yes	27	No	22	No	1.5
15-19	No	Yes	Yes	R501 - R2,500 pm	Yes	5 or more	Yes	one	No	Yes	No	21	No	30	No	1.7
20-24	Yes	No	Yes	.	No	5 or more	No	one	No	Yes	No	33	No	48	No	1.9
20-24	Yes	No	Yes	R501 - R2,500 pm	Yes	5 or more	Yes	one	No	Yes	No	27	No	24	Yes	0.6
20-24	No	Yes	Yes	R501 - R2,500 pm	Yes	5 or more	Yes	2 or more	No	Yes	No	31	No	15	Yes	0.6
20-24	No	Yes	Yes	R501 - R2,500 pm	No	5 or more	Yes	one	No	Yes	No	26	No	14	No	1.3
20-24	Yes	Yes	Yes	R2,501 - R6,000 pm	Yes	5 or more	Yes	one	Yes	Yes	Yes	28	No	27	No	1.7
15-19	Yes	Yes	Yes	R501 - R2,500 pm	No	5 or more	No	one	Yes	Yes	No	26	No	38	No	1.6

20-24	Yes	Yes	Yes	greater than R6,000 pm	Yes	5 or more	Yes	one	No	Yes	No	31	No	21	No	1.5
20-24	Yes	Yes	Yes	greater than R6,000 pm	No	5 or more	Yes	one	No	Yes	No	25	No	29	No	1.4
20-24	Yes	Yes	Yes	R501 - R2,500 pm	No	5 or more	No	2 or more	No	Yes	No	28	No	19	No	1.2
15-19	Yes	No	No	R2,501 - R6,000 pm	Yes	5 or more	Yes	one	No	No	No	22	No	19	No	1.8
15-19	No	No	Yes	R2,501 - R6,000 pm	Yes	5 or more	Yes	none	.	Yes	No	24	.	53	No	1.4
20-24	No	Yes	No	R2,501 - R6,000 pm	No	5 or more	Yes	one	No	No	No	28	Yes	25	No	1.9
15-19	No	Yes	Yes	greater than R6,000 pm	No	5 or more	No	one	No	No	No	30	No	21	No	1.3
20-24	Yes	Yes	Yes	R2,501 - R6,000 pm	No	5 or more	Yes	one	No	No	No	35	No	27	No	1.3
15-19	Yes	Yes	Yes	R2,501 - R6,000 pm	Yes	5 or more	Yes	one	No	Yes	No	36	No	14	No	1.3
20-24	Yes	Yes	Yes	R2,501 - R6,000 pm	Yes	5 or more	Yes	one	No	Yes	No	34	No	21	No	1.4
20-24	No	Yes	Yes	R501 - R2,500 pm	No	5 or more	No	.	No	No	No	32	No	51	No	1.4
20-24	Yes	Yes	Yes	R2,501 - R6,000 pm	Yes	5 or more	Yes	one	No	No	No	29	No	10	No	1.8
20-24	Yes	Yes	Yes	R2,501 - R6,000 pm	Yes	5 or more	No	one	No	No	No	27	No	20	No	1.5
20-24	Yes	Yes	Yes	greater than R6,000 pm	Yes	5 or more	Yes	one	No	Yes	No	26	No	13	No	1.5
20-24	No	No	No	R2,501 - R6,000 pm	Yes	5 or more	Yes	one	No	No	No	31	No	21	No	1.7
20-24	No	Yes	No	R501 - R2,500 pm	Yes	5 or more	Yes	one	No	Yes	No	25	No	28	No	1.3
20-24	Yes	Yes	Yes	R501 - R2,500 pm	No	5 or more	Yes	one	No	Yes	No	30	No	12	No	1.3
15-19	No	Yes	No	R2,501 - R6,000 pm	Yes	5 or more	Yes	one	No	Yes	Yes	27	No	15	No	1.1
20-24	Yes	Yes	Yes	greater than R6,000 pm	No	5 or more	Yes	one	Yes	No	No	26	No	52	No	1.1
20-24	Yes	Yes	Yes	R2,501 - R6,000 pm	No	5 or more	No	one	No	No	No	31	No	13	No	1.4
20-24	No	Yes	Yes	R2,501 - R6,000 pm	Yes	5 or more	Yes	one	No	Yes	No	28	No	36	No	1.3
20-24	Yes	No	Yes	R501 - R2,500 pm	No	5 or more	Yes	one	No	No	No	22	No	20	No	1.1
20-24	No	No	Yes	R501 - R2,500 pm	Yes	5 or more	Yes	one	No	No	No	26	No	39	No	1.5
20-24	Yes	No	Yes	R2,501 - R6,000 pm	Yes	5 or more	No	one	No	Yes	No	22	No	10	No	1.3
20-24	Yes	No	Yes	R2,501 - R6,000 pm	Yes	5 or more	Yes	one	No	Yes	No	26	No	59	No	1.1
20-24	Yes	No	Yes	greater than R6,000 pm	Yes	5 or more	Yes	one	No	No	No	28	No	22	No	1.1
20-24	Yes	No	Yes	R501 - R2,500 pm	No	5 or more	Yes	one	No	Yes	No	28	No	30	No	1.4
