# Supplementary material for: HIV incidence and associated risk factors in adolescent girls and young women in South Africa: A population-based cohort study
Source: PLoS One. 2022 Dec 21;17(12):e0279289. doi: 10.1371/journal.pone.0279289 (PMC9770356; doi:10.1371/journal.pone.0279289)
Supplement: Attachment — Submitted filename: Response to Reviewers PONE-D-22-19202.docx [file pone.0279289.s002.docx]

2 September 2022

Dear Dr Sharifi,

**Response to review of manuscript PONE-D-22-19202**

Thank you for the feedback from the PLOS ONE editorial team and reviewers on our manuscript entitled “HIV incidence and associated risk factors in adolescent girls and young women in South Africa: A population-based cohort study”. We appreciate the opportunity to revise this manuscript. Below we provide a point-by-point response to each of the editor/reviewer comments, with reference to parts of the manuscript that have been updated. We look forward to hearing back from you.

Regards

Lara Lewis

CAPRISA, University of KwaZulu-Natal

1. Editorial comments
   1.1 Journal Requirements:

Please ensure that your manuscript meets PLOS ONE's style requirements, including those for file naming. The PLOS ONE style templates can be found at 
[https://journals.plos.org/plosone/s/file?id=wjVg/PLOSOne_formatting_sample_main_body.pdf](https://protect-za.mimecast.com/s/Es4VC8qYNRHxL5ECM6Zza?domain=journals.plos.org) and 
[https://journals.plos.org/plosone/s/file?id=ba62/PLOSOne_formatting_sample_title_authors_affiliations.pdf](https://protect-za.mimecast.com/s/tMPSC98XNVsP9WDsPEUm_?domain=journals.plos.org)

*Response – We have made the following changes to the manuscript:*

- *Reformatted title page to closely align with guidelines i.e., increased the size of the title, removed abbreviations for organisation names, and added additional affiliations that have arisen since the original submission of this manuscript*
- *Reformatted manuscript text page to closely align with guidelines i.e., ensured there is a space before a citation in the text, reformatted the tables to closely align with guidelines, ensured first line of paragraph was indented, rename “Methods” section to “Materials and methods” and removed the section entitled “Role of Funder/Sponsor”.*
- *Revised the layout of some references*
- *Added a name for supporting information file “S1. Sample dataset”*
  1.2 You indicated that you had ethical approval for your study. In your Methods section, please ensure you have also stated whether you obtained consent from parents or guardians of the minors included in the study or whether the research ethics committee or IRB specifically waived the need for their consent.

*Response - The following text was added to the Methods section: “Written informed consent was obtained from participants 18 years and older and parental/guardian /caregiver consent (South Africans Children’s Act of 2007) for participants 15 to <18 years of age and individual assent from participants 15 to <18 years of age for study participation.”*

1.3 In the ethics statement in the Methods and online submission information, please ensure that you have specified (1) whether consent was informed and (2) what type you obtained (for instance, written or verbal, and if verbal, how it was documented and witnessed). If your study included minors, state whether you obtained consent from parents or guardians. If the need for consent was waived by the ethics committee, please include this information. If you are reporting a retrospective study of medical records or archived samples, please ensure that you have discussed whether all data were fully anonymized before you accessed them and/or whether the IRB or ethics committee waived the requirement for informed consent. If patients provided informed written consent to have data from their medical records used in research, please include this information.

*Response - The following text was added to the Methods and online submission information: “Written informed consent was obtained from participants 18 years and older and parental/guardian /caregiver consent for participants 15 to <18 years of age and individual assent from participants 15 to <18 years of age for study participation. A separate written informed consent was obtained for long term sample storage for confirmation of any discrepant or uncertain results and for future testing if indicated. Each participant was assigned a unique study participant identification number so that their personal data or laboratory results could not be linked to any personal identifiers such as name.”*

1.4 We note that you have included the phrase “data not shown” in your manuscript. Unfortunately, this does not meet our data sharing requirements. PLOS does not permit references to inaccessible data. We require that authors provide all relevant data within the paper, Supporting Information files, or in an acceptable, public repository. Please add a citation to support this phrase or upload the data that corresponds with these findings to a stable repository (such as Figshare or Dryad) and provide and URLs, DOIs, or accession numbers that may be used to access these data. Or, if the data are not a core part of the research being presented in your study, we ask that you remove the phrase that refers to these data.

*Response – this phrase referred to results that were not core to the manuscript so it has been removed.*

2. Reviewer 1 comments

This is a cohort study aiming to identify determinants of HIV incidence in adolescent girls and young women in a hyperendemic setting in South Africa. It is a very interesting study; however, it presents some gaps, especially in the methodology, and must be improved.

2.1 I missed the sample size estimation in the Methods section. Is your sample size statistically significant, and the findings of this study can be generalized?

*Response - Thank you for this observation. We have added a reference to the study protocol in the Methods section as follows: “Further details of the study have been previously published [**26**,* *5**].” The HIPSS protocol paper outlines the sample size calculations used in the study. In short, the HIPSS study was powered to detect a difference in the HIV incidence in the two study cohorts. The sample size provided 84 % power to detect a 30 % reduction in HIV incidence rate at a 5 % significance level, given an HIV prevalence of 20 %, loss-to-follow-up of 15 % per annum and an initial HIV incidence rate of 3 per 100 person years. This particular study was based on the results from a sub-analysis of HIPSS data and does not look at the change in incidence but rather predictors of incidence. In total there were 163 HIV seroconversions – a considerably high number given the limited age range of the sample – and these events allowed us to perform an extensive analysis of potential predictors of HIV.*

2.2 The inclusion and exclusion criteria are not clearly stated. They should be presented in the Study design and setting section.

*Response - Criteria for inclusion into the cohort were based on age, HIV status and willingness to provide blood samples for laboratory testing. Only one person was selected per household and this person was selected at random among the eligible individuals in a household. We have outlined these criteria in the following lines in the Methods section:*

*“One individual per household was selected at random and enrolled in the survey on condition they were aged between 15 and 49 years and provided peripheral blood samples for laboratory HIV and pregnancy testing.”*

*“Individuals were enrolled in the cohorts if they were HIV negative at survey enrolment and aged between 15 and 35 years.”*

2.3 An important piece of information not available in the paper is how the two cohorts were linked.

*Response – Thank you for identifying this important gap. We have amended the Methods section to clarify this by including the following text: “HIPSS comprised of two serial cross-sectional household surveys, with two embedded HIV-negative cohorts comprising of a single follow-up visit. The first survey was conducted between June 2014 and June 2015 and the second between June 2015 and June 2016, and the follow-up visits were completed by January 2017 and August 2017 respectively. Fingerprint biometrics were used to confirm the identity of eligible participants for the follow-up visit. Individuals could be included in both surveys and cohorts if selected, however the overlap was minimal [**5**].”*

2.4 How did you analyze the associated factors by statistical or theoretical criteria? In fact, the authors didn’t focus on how this analysis was done in the Statistical analysis section, although this is one of the paper's objectives.

*Response - Factors included in analysis were selected based on their availability in the HIPSS survey and evidence of association in past literature, as stated in the text: “Underlying and proximate determinants comprised socio-demographic, behavioural and biological factors that were measured in the HIPSS survey and ones that have been determined as influencing HIV incidence in literature [**20**,* *27**,* *28**].”*

*The analysis was structured using a proximate determinants framework as outlined in the section entitled “Conceptual approach”. Cox proportional hazards modelling was then used to model the association between potential risk factors and HIV incidence. We performed a univariable regression followed by a multivariable and we have amended the statistical section to make this clearer: “The association between identified underlying and proximate determinants and HIV incidence was estimated using Cox proportional hazards models. Since data on orphan status and STI testing data was only collected for one of the two cohorts, orphan and STI status were excluded from the Cox regression although included in descriptive analysis. Univariable regression was first performed followed by multivariable regression. All variables included in the univariable were included in the multivariable regression as all variables were hypothesized to be associated with HIV incidence.”*

*A significance level of 0.05 was used to guide whether associations were considered to be strong or not. The direction of the association – whether it was positive or negative – was determined by whether the hazard ratio was above or below 1.*

2.5 Why did you use logistic regression to estimate the association between the underlying and proximate determinants significantly associated with HIV incidence?

*Response - The proximate determinants found to be significantly associated with HIV incidence were: number of partners during follow-up (2 or more vs 1), partner(s) reported HIV and ART status (at least one positive and not on ART vs not), partner circumcision status (all circumcised vs not) and condom use (inconsistent or no condom use vs consistent condom use). As these variables are all binary, a logistic regression was the appropriate choice of model for the analysis when analysing the association between these variables and underlying determinants.*

2.6 Role of the Funder/Sponsor: This section can be stated at the end of the paper, after the conclusions.

*Response - The PLOS ONE's style requirements (found here:* [*https://journals.plos.org/plosone/s/file?id=wjVg/PLOSOne_formatting_sample_main_body.pdf*](https://journals.plos.org/plosone/s/file?id=wjVg/PLOSOne_formatting_sample_main_body.pdf) *) did not indicate that we could put this section after the conclusions section. We have removed this section as it will be covered in the financial disclosure section if successfully published.*

2.7 Association between underlying and proximate determinants: This analysis is very confusing! I think a hierarchical analysis model would be better in this context, splitting by underlying and proximate determinants. In this way, the authors could express the analysis in HR instead of OR.

*Response - The association between underlying and proximate determinants and HIV incidence was measured using multivariable Cox regression with results reported in Table 3. This analysis looked at the association between a factor and HIV incidence after adjusting for all other measured factors. Following this analysis, we looked at the association between underlying determinants and proximate determinants as presented in table 4. This analysis was designed to: “explore how underlying determinants may influence proximate determinants of HIV incidence”. We hypothesize- based on the proximate determinants framework - that underlying determinants “operate through proximate determinants to influence the likelihood of being exposed to HIV”. Thus, to test this hypothesis we analyse the association between underlying and proximate determinants with the proximate determinants as the outcome variable. As the proximate determinants variables are binary in nature and have no time element, we cannot use a time-to-event analysis like Cox proportional hazards modelling for this analysis. Logistic regression is a suitable chose for analyses with a binary outcome variable.*

2.8 Minor issues: It is necessary to add a space between some words and references throughout the text.

*Response - Thank you, this has been addressed.*

3. Reviewer 2 comments

This is an important study in adolescent and young females at risk of acquiring HIV with a detailed assessment of socioeconomic factors associated. Few comments:

3.1 I would add in the introduction the access of the population studied to preventive tools (testing, attesting, condoms and prep) if that is the case.

*Response - Thank you. We have added this sentence to the Study design and setting section: “Contraceptive services and HIV testing and treatment are freely available through primary health care clinics and, since 2016, oral pre-exposure prophylaxis (PrEP) has been made available to people at substantial risk of HIV infection.” To illustrate that PrEP usage was low in the cohort, we have included frequency of PrEP usage to table 1.*

3.2 In the result section I would change the column of baseline characteristics of 15-24 yo to "Total of participants", to make it more clear for readers.

*Response - Thank you. We have amended this column to have the heading “Total”.*

3.3 The 27% of participants not completing the follow-up, could you provide the characteristics as well in the text? It might be that those have several differences in their social determinants that may have drive them to stop the follow up and I think that is important to address.

*Response - Thank you. 23% were not accessible for follow-up. We have added the following text to the results section to provide some insight into this group: “Compared to those with follow-up data, HIV-negative AGYW who were not available for follow-up (n=808, 23.0%) were more likely to be aged between 20 and 24 (48.2% versus 54.7%) and reside in a rural area (51.7% versus 76.6%). However, the two groups were comparable with respect to education levels, household income, number of lifetime sexual partnerships and STI status at baseline.”*

3.4 Regarding the missingness of many variables, I think it would be fair to discuss this more in detail in the discussion section, although it is established as a limitation, I think it deserves more potential explanation to that.

*Response - Thank you for identifying this. We have added the following text to the discussion section: “Missing data can lead to a loss of statistical power or introduce an unexpected selection bias. However, in this study the number of missing responses to questions in the baseline survey and cohort follow-up visit were generally low, and a comparison of characteristics of those lost to follow-up to those who were followed did not suggest that the two groups were inherently different.”*

4. Reviewer 3 comments

   Lewis and colleagues studied socio-demographic, behavioural and biological determinants of HIV incidence among adolescent girls and young women (AGYW) in South Africa. They used a proximate determinants framework to define pathways through which HIV infections could occur in this vulnerable group. The authors concluded that retaining AGYW in school, and having their partners access voluntary medical male circumcision, utilize condoms and be on HIV treatment as prevention all reduce risks of their acquisition of HIV. They also concluded that family support reduces AGYW risky sexual behaviour and HIV acquisition. The authors therefore highlighted the importance of designing and implementing community-based HIV combination prevention programs that effectively address the identified structural, biological and behavioural HIV risk factors in their design. The research findings are fully consistent with the existing literature and add value to the body of evidence in regard to the causal pathways of HIV acquisition. The manuscript has a number of strengths. It measures the relationships between both proximate and underlying determinants with HIV incidence and elucidates how underlying determinants influence proximate determinants. It also elucidates causal pathways for the HIV acquisition. Finally, it measures HIV incidence levels in sub-groups of AGYW in this area. One study weakness is that there may be social desirability bias which could have led to underreporting of risky sexual behaviour. The authors however acknowledge this weakness to be taken into account in the interpretation of the study findings. My overall recommendation is that this is a technically sound manuscript that is well written. It should be accepted with very minor revisions.

*Response - Thank you for reviewing our manuscript and for your encouraging feedback!*

4.1 In the introduction, line 62, you could consider specifying to what extent the risk in this sub-group has declined, and to what extent the risk of HIV remains substantial.

*Response –While all referenced manuscripts suggest that incidence is declining, the magnitude of the reported declines differs by methodology and location of research. As such, we have decided not to include this detail. The extent of the risk of HIV for AGYW in this region is summarised in the line “approximately 4,200 AGYW became infected with HIV every week in 2020 [**2**].”*

4.2 In line 65 also in the introduction, you could consider replacing “campaigns” with another word, like “efforts”.

*Response- We have changed this to ‘programmes’.*

4.3 Line 223-225 in the results section refers to AGYW 15-19 years old who received support. The subsequent sentence (line 225-226) gives the incidence among AGYW who did not receive support. Though the incidence among AGYW who received support in included in Table 1, for easier comparison, it could help to include the incidence among those who received support in the text.

*Response - We have added this detail: “Among those that did not receive this support, HIV incidence was 10.40 (95% CI: 5.67-19.09) per 100 person-years compared to 3.17 (95% CI: 2.38-4.21) among those who did.”*

4.4 For line 229-231 also in the results section, it may be important to report what proportion of AGYW who reported knowingly having sex with individuals who were HIV positive and not on ART did not use protection.

*Response - Only 5(10%) of the 48 reported consistent condom use in the baseline and follow-up questionnaires. We have not added this detail as it could not be added to Table 1 and hence reference to the result may have confused the reader.*
