# Supplementary material for: HIV incidence and associated risk factors in adolescent girls and young women in South Africa: A population-based cohort study
Source: PLoS One. 2022 Dec 21;17(12):e0279289. doi: 10.1371/journal.pone.0279289 (PMC9770356; doi:10.1371/journal.pone.0279289)
Supplement: Attachment — Submitted filename: Response to Reviewer.docx [file pone.0279289.s003.docx]

13 November 2022

Dear Dr Sharifi,

**Response to review of manuscript PONE-D-22-19202**

Thank you for the feedback from the PLOS ONE editorial team and reviewers on our manuscript entitled “HIV incidence and associated risk factors in adolescent girls and young women in South Africa: A population-based cohort study”. We appreciate the opportunity to revise this manuscript. Below we provide a response to each of the editor/reviewer comments, with reference to parts of the manuscript that have been updated. We look forward to hearing back from you.

Regards

Lara Lewis

CAPRISA, University of KwaZulu-Natal

1. Journal Requirements:

Please review your reference list to ensure that it is complete and correct. If you have cited papers that have been retracted, please include the rationale for doing so in the manuscript text, or remove these references and replace them with relevant current references. Any changes to the reference list should be mentioned in the rebuttal letter that accompanies your revised manuscript. If you need to cite a retracted article, indicate the article’s retracted status in the References list and also include a citation and full reference for the retraction notice.

We have reviewed all references and updated accordingly.

2. Reviewer 2 comments

This is an important Study in a specific population (female adolescents) at high risk of acquiring HIV and their findings could help to design future interventions.
Some comments and editions:

Abstract: In results authors do not describe the overall results, they only describe the risk factors in 2 groups: those 15-19 and 20-24 years. It should be describen first the overall results and then perhaps divided according to range of ages. Additionally, division of ranges of ages, are not pre-specified in the methods sections. Authors, should describe all findings in the group of 15-19 years, and then all the results of population of 20-24 years in order to read it better.

Thank you. We have re-worded the results so that the 15-19-related results appear first followed by those for the 20-24 year-olds. We have added to the methods that separate models were built for the two age groups. In the body of the manuscript, we explain why we took the approach to split the age groups: “Analyses were performed separately for 15-19-year-olds and 20-24-year olds as we hypothesized that the factors affecting young girls who are still of school going age are likely to be different from those affecting women who are out of secondary-school, possibly in tertiary education or seeking employment.” We provide overall incidence results in the abstract, but since separate models were used for measuring the associations for the two age groups, we cannot combine these results to one overall estimate.

Intro: I could suggest to add something about the fact that adolescent population have proven in clinical trials to be a very challenging population to enroll, adhere and retain in treatment and prevention of HIV... therefore, understand the characteristics of those acquiring HIV would help to design better interventions. Methods: Here are my main concerns about this study... What if through the multistage sampling (only one household enrolled) you are missing those who are at most risk...? Additionally, only 77% completed. I would analyze the population that completed vs those who did not, in order to show if those are different population. If they are, then results should be interpret with more caution. On the other hand, is there a national registry in which you could compare your incident cases with those registered... It might be that the incidence is different from the reality, just because the sampling could miss those more at risk...?

Thank you for raising this important concern. The survey enrolled one randomly selected person per household (not one household). Our incidence is higher than that reported based on the national estimates for 15-24 year olds (South African National HIV Prevalence, Incidence, Behaviour and Communication Survey, 2017). This is expected given the high HIV prevalence in the region in which the survey was conducted but also provides us with reassurance that our selection – while random – were not unduly biased by refusal rates. The original version of this manuscript was updated with this text in the manuscript: “Compared to those with follow-up data, HIV-negative AGYW who were not available for follow-up (n=808, 23.0%) were more likely to be aged between 20 and 24 (48.2% versus 54.7%) and reside in a rural area (51.7% versus 76.6%). However, the two groups were comparable with respect to education levels, household income, number of lifetime sexual partnerships and STI status at baseline.”

Additionally, in methods you should pre specify the way you are going to analyze and stratify your population. In results you present different groups: 15-19 years, 20-24 years, then the analysis of those with less education level, also those without sexual history, etc. All groups should be described in methods and the rationale of those divisions.

The reason for splitting the age groups is provided in the methods: “Analyses were performed separately for 15-19-year-olds and 20-24-year olds as we hypothesized that the factors affecting young girls who are still of school going age are likely to be different from those affecting women who are out of secondary-school, possibly in tertiary education or seeking employment.”

We outline where we exclude those without sexual history and why here in the methods: “Unless otherwise stated, all AGYW, regardless of whether they reported having sex before study enrolment, were included in the analysis. However, models that incorporated sexual behaviour variables that were based on follow-up data excluded, by necessity, AGYW who reported not being sexually active in the 12 months preceding follow-up.”

The analysis of the association between education and other sexual behavioral variables is explained here in the methods: “The association between the underlying determinants and proximate determinants found to be significantly associated with HIV incidence was measured using logistic regression.”

Results: Comparison with their peers male is presented, not explained in methods how this population was analyzed or picked... It is a very interesting comparison, but it has to be explained in methods in order to see that the comparison is fair and reliable.

We are uncertain which part of the manuscript this comment relates to. We think this comment relates to an earlier version of the manuscript where an incidence of 0.82 was reported for male peers. This line was removed in the first revision of the manuscript for the reasons the reviewer provides.

Finally, the data were collected from 2014-2017... prevention programs have changed in the last decade. I would like to know why is the data so old and discuss this issue in the discussion section and as a limitation.

The efforts of the authors writing the manuscript were redirected to COVID-19 work during 2020 and 2021. We have now highlighted the age of the data in the limitations section. While HIV prevention programmes have changed in the last decade, determinants of risk in young women appear to remain quite consistent – education, family support age-disparate partnerships and high STI prevalence repeatedly present themselves as related factors.
